# The Localization and Action of Topoisomerase IV in *Escherichia coli* Chromosome Segregation Is Coordinated by the SMC Complex, MukBEF

**DOI:** 10.1016/j.celrep.2015.11.034

**Published:** 2015-12-10

**Authors:** Pawel Zawadzki, Mathew Stracy, Katarzyna Ginda, Katarzyna Zawadzka, Christian Lesterlin, Achillefs N. Kapanidis, David J. Sherratt

**Affiliations:** 1 Department of Biochemistry, University of Oxford, South Parks Road, Oxford OX1 3QU, UK; 2 Biological Physics Research Group, Clarendon Laboratory, Department of Physics, University of Oxford, Parks Road, Oxford OX1 3PU, UK; 3 Bases Moléculaires et Structurales des Systèmes Infectieux, UMR 5086, Centre National de la Recherche Scientifique, University of Lyon, 69367 Lyon, France

## Abstract

The type II topoisomerase TopoIV, which has an essential role in *Escherichia coli* chromosome decatenation, interacts with MukBEF, an SMC (structural maintenance of chromosomes) complex that acts in chromosome segregation. We have characterized the intracellular dynamics of individual TopoIV molecules and the consequences of their interaction with MukBEF clusters by using photoactivated-localization microscopy. We show that ∼15 TopoIV molecules per cell are associated with MukBEF clusters that are preferentially localized to the replication origin region (*ori*), close to the long axis of the cell. A replication-dependent increase in the fraction of immobile molecules, together with a proposed catalytic cycle of ∼1.8 s, is consistent with the majority of active TopoIV molecules catalyzing decatenation, with a minority maintaining steady-state DNA supercoiling. Finally, we show that the MukB-ParC interaction is crucial for timely decatenation and segregation of newly replicated *ori* DNA.

## Highlights

• Individual molecules of topoisomerase IV (TopoIV) were tracked in live *E. coli* cells
• TopoIV was monitored in cellular space and in time throughout the cell cycle
• The interaction of TopoIV and MukBEF directs TopoIV to its sites of action
• The TopoIV-MukBEF interaction promotes timely segregation of newly replicated DNA

## Graphical Abstract

**Figure:**
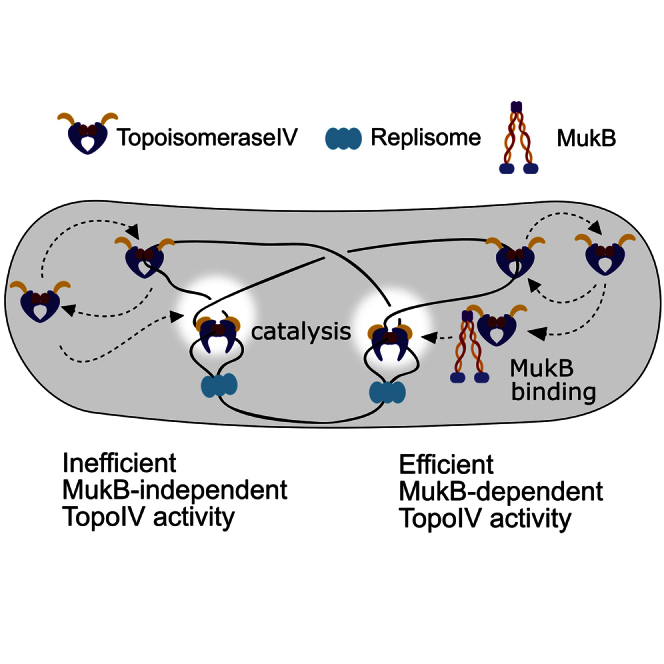

## Teaser

Zawadzki et al. use quantitative single-molecule imaging to examine the behavior of topoisomerase IV molecules in their native environment inside *Escherichia coli* cells. The work reveals how the functional interaction between topoisomerase IV and the SMC complex, MukBEF, aids chromosome segregation.

## Introduction

Segregation of newly replicated chromosomes must be completed before cell division can occur. Two classes of proteins play important roles in DNA segregation: topoisomerases and structural maintenance of chromosome (SMC) complexes.

DNA replication introduces positive (+) supercoiling ahead of the replication fork, and rotation of the forks leads to interlinking of the two sister chromosomes, generating (pre)catenanes behind the replisomes throughout the chromosome. In *Escherichia coli*, ∼225,000 catalytic events by the type II topoisomerases DNA gyrase and topoisomerase IV (TopoIV) are required for each generation to unlink the 4.6-Mb chromosome. DNA gyrase acts preferentially ahead of the replication fork to remove (+) supercoiling (Koster et al., 2010, Vos et al., 2011). TopoIV removes the majority of links behind the replication forks (Joshi et al., 2013, Wang et al., 2008), whereas the type I topoisomerase TopoIII is able to remove links in single-stranded DNA regions (Koster et al., 2010, Vos et al., 2011) and FtsK-dependent XerCD recombination at *dif* is able to remove catenation links in *ter* (Grainge et al., 2007).

Heterotetrameric TopoIV consists of dimers of ParC (the DNA binding and catalytic subunit) and ParE (the regulatory ATPase). It changes DNA topology by introducing a double-stranded break in DNA and passing a second duplex segment of DNA through the break before resealing it. TopoIV acts on topologically different substrates including (+) and negative (−) supercoiled DNA and knotted and catenated DNA (Koster et al., 2010, Postow et al., 2001, Vos et al., 2011). Its essential cellular role is in decatenation of newly replicated DNA (Joshi et al., 2013, Wang et al., 2008). The mechanism of how TopoIV recognizes and discriminates its substrates and which substrate is preferred in vivo is not fully understood (Lee et al., 2013, Vos et al., 2013a).

A second class of proteins, SMC complexes, play an equally important role in faithful DNA segregation (Hirano, 2006). Despite sharing little primary amino acid sequence homology with other SMC complexes, the *E. coli* complex MukBEF retains much of the distinctive SMC architecture (Nolivos and Sherratt, 2014, Woo et al., 2009), forming dimers joined at a hinge domain located at one end of an ∼50-nm-long intramolecular coiled coil with an ATPase head domain at the other end of the coiled coil. Inactivation of the MukB protein or either of the two accessory proteins, MukE and MukF, results in abnormal chromosome organization and segregation (Danilova et al., 2007, Nolivos and Sherratt, 2014). The MukB dimerization hinge has been shown to physically interact in vitro with ParC, which stimulates TopoIV-mediated relaxation of (−) supercoils (Hayama and Marians, 2010, Li et al., 2010). An enrichment of ParC/E molecules in the vicinity of *ori*-associated MukBEF clusters was observed in widefield imaging (Nicolas et al., 2014).

Here, we used super-resolution microscopy to characterize the behavior of single molecules of TopoIV in live *E. coli*. Moreover, by perturbing the action of TopoIV molecules using genetics, an inhibitor, and overexpression of competing protein domains, we are able to provide mechanistic insight into the function of TopoIV and its interaction with MukBEF clusters. Using photoactivated-localization microscopy (PALM) combined with single-particle tracking (Manley et al., 2008), we show that ∼60 molecules of TopoIV were present at any time, although sufficient ParC and ParE subunits were present for ∼105 TopoIV molecules. Impairing the interaction between functional TopoIV and MukBEF, by overexpressing a competing but non-functional ParC C-terminal domain, resulted in an ∼2-fold reduction in the number of immobile TopoIV molecules, consistent with the interaction between TopoIV and MukBEF directing the location and catalytic action of TopoIV molecules toward *ori*-associated MukBEF clusters. We identified two populations of immobile TopoIV molecules; we propose that one with a dwell time of ∼1.8 s identifies catalytically active molecules, while the other, with a dwell time of ∼30–70 ms, identifies molecules bound to MukBEF clusters. Wide-field, PALM, and 3D-structured illumination microscopy (3D-SIM) (Allen et al., 2014) demonstrated that MukBEF clusters were enriched along the long axis of the cell. Furthermore, we found that the MukB-ParC interaction, although not essential for TopoIV function, is crucial for timely segregation of newly replicated *ori* DNA. Impairing this interaction caused delayed segregation of newly replicated sister *ori*s, consistent with the MukBEF-ParC interaction enhancing decatenation of newly replicated DNA.

## Results

### A Fraction of TopoIV Subunits Are in TopoIV Heterotetramers

**Figure 1:**
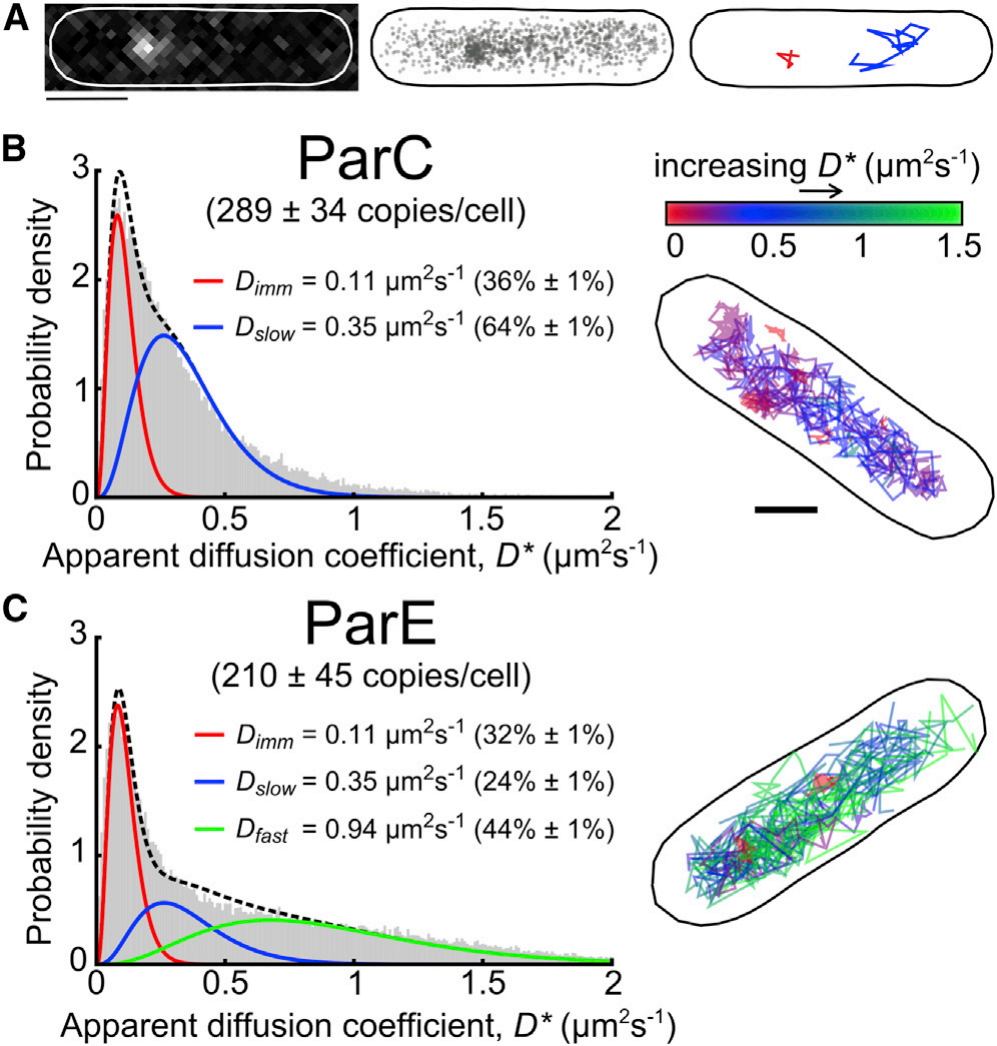
Tracking PALM of *E. coli* ParC/E Molecules (A) Example image of a single ParC-PAmCherry molecule at 15 ms exposure (left), super-resolved localizations derived from all frames and for all molecules detected in this cell (middle), and example tracks of individual slow ParC (blue) and immobile ParC (red) molecules (right). Scale bar, 1 μm. (B) Distribution of apparent diffusion coefficients (*D^∗^*) of 73,020 tracked ParC molecules, fitted with a two-species model. Ranges indicate 95% confidence interval. Example cell with individual trajectories colored according to their *D^∗^* value. (C) Distribution of *D^∗^* values for 64,551 ParE molecules fitted with a three-species model. Copy numbers of ParC and ParE subunits, normalized for cells 2.5 μm long, were determined by sequentially photoactivating and tracking all available molecules.

To characterize the copy number and behavior of TopoIV heterotetramers in live *E. coli* cells, we labeled the ParC or ParE subunits by replacing the endogenous genes with functional C-terminal fusions to the photoactivable fluorophore, PAmCherry. The fusions were fully functional in in vivo assays (Supplemental Experimental Procedures; Figure S1A; Table S3). Cells were imaged with a PALM microscope and individual molecules localized in each frame. Linking consecutive localizations into trajectories allowed us to follow the movement of individual ParC/E molecules at 15-ms intervals until photobleaching (Figure 1A) (Manley et al., 2008, Uphoff et al., 2013). 289 ± 34 photoactivatable molecules of ParC and 210 ± 46 photoactivatable molecules of ParE, normalized to a 2.5-μm-long cell, were counted. Since the photactivation efficiency of PAmCherry was determined to be ∼50% in vivo, the actual copy numbers are likely to be approximately two times higher than these values (Supplemental Experimental Procedures).

To measure the mobility of ParC/E, we calculated an apparent diffusion coefficient (*D^∗^*) for each molecule from the one-step mean squared displacement (*MSD*) of its trajectory using *D^∗^* = MSD/(4 Δt), where Δt is the frame time of 15 ms. The different diffusing populations, which could not be described by a single diffusing species (Figure S1B), were defined by fitting an analytical expression to the distribution of experimental *D^∗^* values (Stracy et al., 2015). We first established the mean *D^∗^* of immobile molecules. Based on a localization error of ∼40 nm, we estimated mean *D^∗^* of immobile molecules to be ∼0.1 μm^2^s^−1^. This was confirmed by fitting to the distribution *D^∗^* values for the previously characterized protein DNA polymerase 1 (where the immobile population was clearly resolvable), showing that *D*_imm_ = 0.11 ± 0.01 μm^2^s^−1^ (Uphoff et al., 2013, Stracy et al., 2015; Figure S1C).

The ParC *D^∗^* distribution fitted well to a two-species model (Figure 1B): an immobile population (36% ± 1%; constrained at *D*_imm_ = 0.11 μm^2^s^−1^) and a second, unconstrained *D* distribution, corresponding to a slowly diffusing population (64% ± 1%; *D*_slow_ = 0.35 ± 0.01 μm^2^s^−1^). Molecules in the slow-diffusing population had a lower mobility than expected for free 3D diffusion, consistent with them undergoing transient interactions with DNA, which ParC does (Corbett et al., 2005). The spatial distribution of slowly diffusing ParC molecules showed that they were associated with the nucleoid, consistent with them being transiently associated with DNA (Figures S1G and S3A). In contrast, we propose that the immobile ParC molecules are relatively stably bound to DNA or DNA-bound proteins.

The ParE *D^∗^* distribution showed a third population of molecules with higher mobility in addition to the two populations similar to those observed for ParC. As ParE does not bind DNA (Lee et al., 2013), we propose that the fast-diffusing molecules represent free ParE subunits, whereas the immobile and slow-diffusing molecules were in TopoIV heterotetramers. To test this, we imaged ParE-PAmCherry molecules in cells in which unlabeled ParE was overexpressed, outcompeting labeled ParE in TopoIV heterotetramers. Consistent with our hypothesis, ∼90% of ParE-PAmCherry molecules now diffused rapidly and were uniformly distributed throughout the cell, showing no bias toward the nucleoid region (Figures S1F and S1G). Fitting a three-species model to this data (with *D*_imm_ and *D*_slow_ constrained) established that *D*_fast_ = 0.94 ± 0.02 μm^2^s^−1^. Conversely, imaging ParE-PAmCherry molecules in a strain overexpressing unlabeled ParC showed that the *D*_fast_ population is completely lost (Figure S1H), confirming that ParE molecules in the *D*_imm_ and *D*_slow_ states are complexed with ParC in TopoIV heterotetramers.

The three-species model for the ParE data, with constrained *D*_imm_, *D*_slow_, and *D*_fast_ values, showed that 32% ± 1% were immobile, 24% ± 1% were slow diffusing, and 44% ± 1% were fast diffusing (Figure 1C). As the copy-number estimates showed that there is no excess ParE in the cell, the 44% of uncomplexed, fast-diffusing ParE molecules must reflect a steady-state level of TopoIV heterotetramer formation and dissociation, with ∼56% of ParE subunits being present in ∼60 TopoIV heterotetramers. Therefore, the ∼60 TopoIV molecules present at any time form from a pool of ParC and E molecules sufficient for ∼105 TopoIV heterotetramers. By using the estimated copy numbers and the ∼1.1 μm^3^ volume of cells 2.5 μm long, we estimated the in vivo dissociation constant of TopoIV heterotetramers to be ∼0.5 μM (Supplemental Experimental Procedures).

### Half of Immobile TopoIV Molecules Result from Interaction with MukB

**Figure 2:**
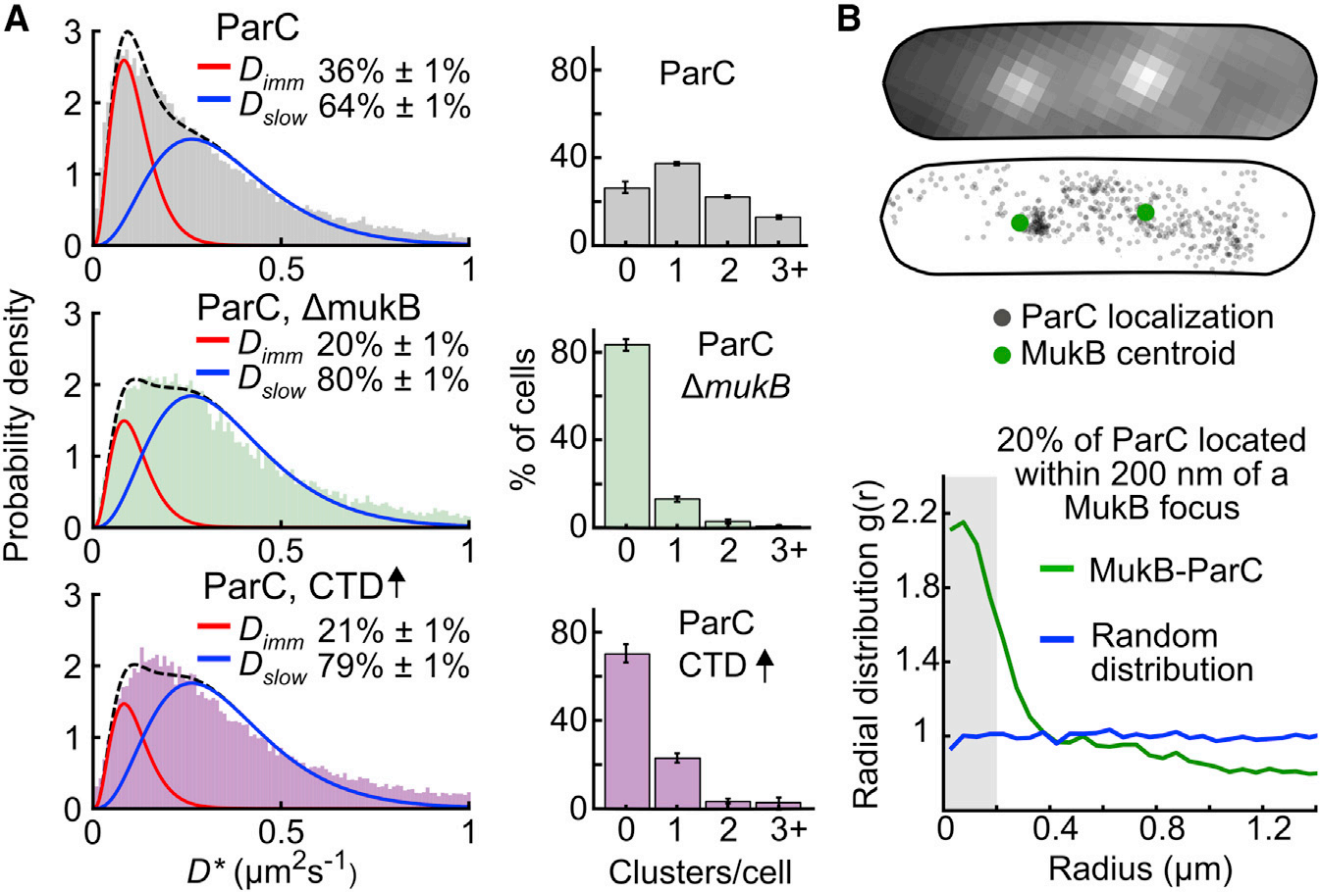
MukBEF Clusters Influence TopoIV Diffusion and Organization (A) Left panels: distribution of *D^∗^* values for ParC molecules fitted with a two-species model with immobile molecules (constrained at *D*_imm_ = 0.11 μm^2^s^−1^) and slow-moving molecules (constrained at *D*_slow_ = 0.35 μm^2^s^−1^). Top: ParC molecules in wild-type cells (from Figure 1B). Middle: 18,971 ParC molecules in Δ*mukB* cells. Bottom: 42,920 ParC molecules after unlabeled ParC-CTD overexpression (3 hr). Ranges give 95% confidence intervals. Right panels: the number of ParC clusters per cell, determined by clustering all localizations using a nearest-neighbor algorithm, in wild-type (2,635 cells) and Δ*mukB* (387 cells) cells and with ParC-CTD overexpression (214 cells). Error bars indicate SD of three experimental repeats. (B) Example cell with MukB-mYPet foci (top) visualized prior to PALM acquisition and localization of ParC-PAmCherry molecules (middle). Radial distribution of ParC localizations from each MukB focus (717 cells), compared to random distribution (bottom). The radial distribution function shows the probability of finding a ParC localization at distance, *r*, from a MukB focus. Gray bar shows localization within 200 nm.

Since ParC interacts with the MukB dimerization hinge in vitro (Hayama and Marians, 2010, Li et al., 2010, Vos et al., 2013b) and shows an enrichment near MukBEF clusters in vivo (Nicolas et al., 2014), we tested whether a fraction of the immobile ParC and ParE molecules result from their binding to immobile MukBEF clusters on DNA. Fitting a two-species model (with the *D*_imm_ and *D*_slow_ populations established previously) to the distribution of *D^∗^* values for ParC molecules in Δ*mukB* or *mukB*^DA^ cells; MukB^DA^ is unable to bind ATP and form *ori-*associated MukBEF clusters (Badrinarayanan et al., 2012), showed a ∼50% reduction in the immobile fraction of ParC/ParE consistent with these molecules being immobile as a consequence of their interaction with *ori-*associated MukBEF clusters (Figure 2A; Figures S2A and S2B). Using a clustering algorithm to define ParC clusters containing ≥25 localizations, we showed that ParC formed a median of one cluster per cell and deletion of MukB removed most ParC clustering (Figure 2A). This was confirmed by the radial distribution analysis of all ParC localizations that showed a strongly clustered distribution, which was reduced ∼4-fold in Δ*mukB* cells (Figure S2C).

Since Δ*mukB* and *mukB*^*DA*^ cells have disorganized chromosomes (Danilova et al., 2007), we also considered whether the reduction in the fraction of immobile ParC/E molecules in these cells was instead a consequence of global chromosome changes. To distinguish these possibilities, we impaired the TopoIV-MukB interaction by overexpressing an unlabeled ParC C-terminal domain (ParC-CTD), which binds MukB (Vos et al., 2013b), thereby outcompeting TopoIV binding. Overexpression of ParC-CTD did not significantly affect growth rate, cell length, or formation of anucleate cells (Table S3), consistent with unperturbed chromosome organization. Flow cytometry profiles showed a small increase in cells with multiple chromosomes (Figure S1A). Under these conditions, the immobile fraction of ParC was reduced to the level in *ΔmukB* cells (Figure 2A, bottom), and clustering of ParC was lost, consistent with approximately half of immobile ParC molecules being dependent on a direct interaction with immobile MukBEF clusters.

To demonstrate that ParC clusters spatially associate with MukBEF clusters, we imaged ParC-PAmCherry and MukB-mYPet in the same cells. Calculating the radial distribution function of ParC PALM localizations with respect to the centroid of each MukBEF focus showed that ParC is enriched near MukBEF foci, which moved very little during the observation period (Figure 2B; Figure S2D), with ∼20% of ParC localizations within 200 nm of MukBEF centroids. This result is consistent with the ∼16% of ParC molecules that were immobile due to a direct interaction with MukB, as judged by the reduction in the fraction of immobile molecules in *ΔmukB* cells (Figure 2A). We noticed that while ParC clusters were nearly always in close proximity to a MukBEF focus, not all MukBEF foci were associated with a ParC cluster, a trend also evident in intensity projections from epifluorescent imaging (Figure S3E). Imaging MukB-PAmCherry with PALM showed that, despite having a similar copy number (195 ± 57 copies/cell; Figure S2F), MukBEF formed approximately twice as many clusters per cell as ParC (Figure S2G), thereby indicating an additional level of regulation governing the MukB-ParC interaction.

By using the fraction of immobile TopoIV molecules dependent on MukB (Figures 1B, 1C, and 2A) and, independently, the fraction of ParC localizations close to MukB in the radial distribution analysis (Figure 2B), we estimated that ∼15 TopoIV molecules were associated with MukBEF clusters at any given time and determined the in vivo dissociation constant of MukB-ParC complexes to be ∼2 μM (Supplemental Experimental Procedures), consistent with in vitro measurements (Li et al., 2010).

To understand further how MukBEF clusters direct the organization of immobile ParC molecules within the nucleoid, we determined the probability density of ParC molecules across the short cell axis. We segmented cell outlines from the bright-field images and determined the intracellular location of the tracks. We then established a *D^∗^* threshold (0.16 μm^2^s^−1^), which preserved the ratio of immobile (36%) to mobile (64%) molecules, established from fitting, to classify each individual ParC track as immobile or mobile. The analysis showed that immobile ParC molecules were preferentially located along the long axis of the cell (Figure S3A). Similar intracellular positioning was observed for immobile MukB-PAmCherry molecules, with an even stronger bias of immobile molecules along the long cell axis (Figure S3A, right). We found a similar pattern of MukBEF cluster enrichment on the long cell axis when we analyzed the distribution of MukBEF foci in epifluorescence images (Figure S3B). In Δ*mukB* cells, immobile ParC molecules showed a lower probability of locating to the cell long axis, consistent with MukBEF clusters recruiting ParC molecules to the long cell axis (Figure S3A, middle). When we co-imaged MukB-mYPet and DAPI-stained DNA with 3D structured illumination microscopy, we also observed MukBEF clusters located along the long cell axis, close to regions of high nucleoid density (Figure S3C; Movie S1; Supplemental Experimental Procedures).

### Two Populations of Immobile TopoIV Molecules

**Figure 3:**
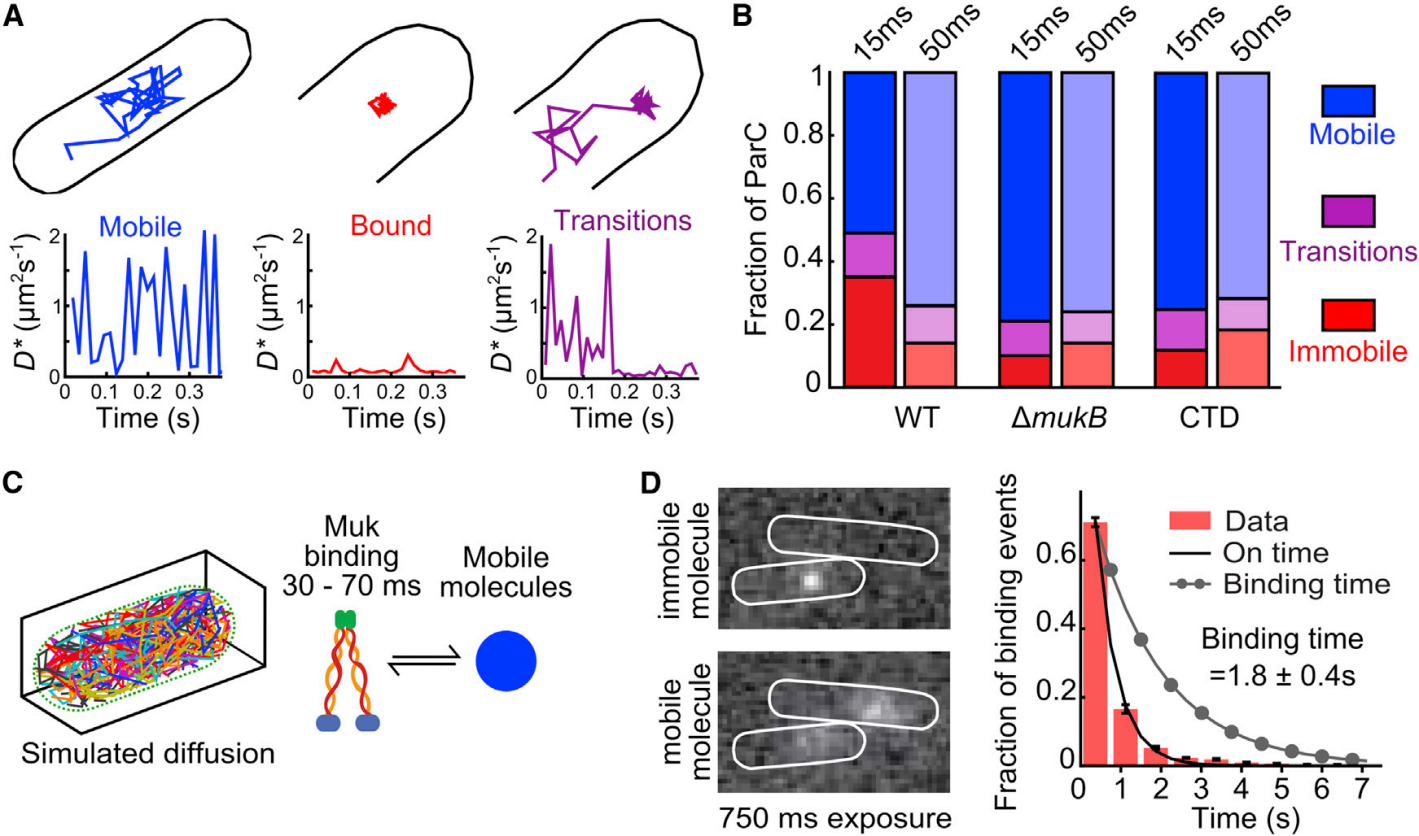
MukB-Dependent and Independent ParC Binding Behavior (A) Examples of long ParC trajectories (ten or more localizations) classified according to their *D^∗^* transitions. Molecules mobile over observation period (blue), immobile (red), and undergoing transition from one state to another (purple). (B) Bar graph of all ParC trajectories for the indicated strains, classified from PALM experiments performed at 15-ms continuous acquisition and time lapse (15-ms exposure + 35-ms delay). (C) Schematic of Markov chain Monte Carlo simulations of molecules inside a typically sized cell volume interconverting between immobile and free diffusion. Cartoon representation of transitions analyzed in simulations. Shown is the time range obtained in simulations that recapitulated the experimental data. (D) Left: example 750-ms exposure frames showing cells with an immobile TopoIV molecule (top) and a mobile molecule (bottom). Right: on-time distributions for immobile ParC with exponential fit (line) and photobleaching-corrected binding time distribution (line with dots). Error bars indicate SD of three experimental repeats.

To dissect TopoIV binding events, we analyzed long trajectories of ten or more localizations and sorted molecules into three categories: mobile molecules that remained above the *D^∗^* threshold for the observation period, immobile molecules that remained below the *D^∗^* threshold over the observation period, and molecules that exhibited transitions between these states (Figure 3A). This analysis detected similar fractions of immobile molecules as determined from fits to the *D^∗^* distributions (compare Figure 3B with Figure 1B). In addition, a fraction of the molecules underwent transitions, consistent with TopoIV molecules being in a dynamic equilibrium between bound and mobile states (Figure 3A, right).

In time-lapse experiments, using 15-ms exposures followed by 35-ms delays, we observed a reduction in the population of ParC molecules that remained immobile over the course of the trajectory from 35% to 14% (Figure 3B). The result was also evident in *D^∗^* distributions (Figure S4A). Molecules in the immobile category in the time-lapse experiments (bound for ten or more localizations with a 50-ms frame time) must remain bound for ≥0.5 s, compared to ≥0.15 s for the immobile molecules in normal 15-ms frame-time experiments (bound for ten or more localizations). The observed reduction in the fraction of immobile molecules shows that ∼21% of the binding events in Muk^+^ cells lasted for ≤0.5 s. In contrast, when we performed the same analysis in Δ*mukB* cells or in cells overexpressing ParC-CTD, the fraction of immobile molecules remained unchanged in normal and time-lapse PALM experiments (Figure 3B). This shows that in wild-type cells, a population of MukB-dependent transiently immobile (≤0.5 s) TopoIV molecules is present alongside molecules immobile for ≥0.5 s.

Because the underlying binding times are exponentially distributed, they cannot be extracted intuitively from experiments. We therefore used Markov chain Monte Carlo simulations to gain a better estimate of the durations of the short-lived MukB-dependent binding events. Molecule trajectories were simulated undergoing Brownian motion inside a confined cell volume (Bakshi et al., 2013, Persson et al., 2013, Uphoff et al., 2013). Molecules were in one of two diffusive states: D_free_ and D_imm_, with transitions allowed between each state. The free diffusion, *D*_*free*,_ of TopoIV heterotetramers was calculated based on the free diffusion of ParE (*D*_*fast*_), correcting for their relative sizes (Supplemental Experimental Procedures). Molecule trajectories were simulated to generate localizations at either 15-ms intervals or 15-ms intervals with 35-ms delays to match normal and time-lapse experiments, respectively. The simulated localizations were analyzed with the same tracking and categorizing algorithm as used for the experimental data. We simulated interconverting molecules with different exponentially distributed binding durations from 0.1 ms to 150 ms (keeping the fraction in each state equal). Plotting the change in the fraction of molecules categorized as bound in time-lapse simulations compared to normal simulations showed that a binding duration of 30–70 ms for MukB-dependent TopoIV transient binding events recapitulated the experimentally observed decrease (Figure 3C; Figure S4D). Furthermore, simulations with a binding time ≪ exposure time showed that a transient (≤1 ms) DNA binding explains well the lower-than-expected mobility of slowly diffusing ParC molecules (Figure S4C).

Finally, we characterized the molecules that remained immobile over the time-lapse experiment observation time (binding time ≥0.5 s). Because our ability to observe complete events was limited by photobleaching, we increased the observation time by using low excitation intensities, sparse photoactivation, and long (≥0.5 s) exposure times, when mobile molecules are motion blurred, whereas immobile molecules appear as point sources, producing a diffraction-limited spot (Elf et al., 2007, Stracy et al., 2014) (Figure 3D). Immobile molecules could therefore be distinguished by the width of the elliptical Gaussian fits to the fluorescent spot. We used thresholds established with Pol1 (with clearly resolvable immobile molecules) of <160 nm short axis width and <200 nm long axis width to identify immobile molecules (Uphoff et al., 2013; Figure S4E). The probability of observing a particular on-time is the product of the underlying binding-time probability and the bleaching probability. The bleaching-time distributions were measured independently with the same acquisition and excitation conditions using MukB-PAmCherry, which binds DNA in clusters with a dwell time longer (∼50 s) than the photobleaching lifetime (Badrinarayanan et al., 2012). We measured ParC on-times at 0.5 s, 0.75 s, and 1 s exposure times and corrected for photobleaching (Uphoff et al., 2013). We found the mean binding time to be 1.8 ± 0.4 s (Table S4).

In conclusion, we have shown that “immobile” TopoIV molecules display two different bound states: a 30- to 70-ms MukBEF binding-dependent state and ∼1.8-s binding events, which we propose identify TopoIV molecules undergoing a single catalytic cycle, since such a binding time is of the same order as measurements of a single TopoIV catalytic cycle in vitro (Crisona et al., 2000, Neuman et al., 2009, Stone et al., 2003). The analysis did not detect longer events that would be expected for processive catalysis. Based on analysis in vitro of processive bursts on (+) supercoiled DNA, they were expected to last tens of seconds (Crisona et al., 2000, Stone et al., 2003).

### TopoIV Molecules Undergoing Catalysis Are Enriched at MukBEF Clusters

**Figure 4:**
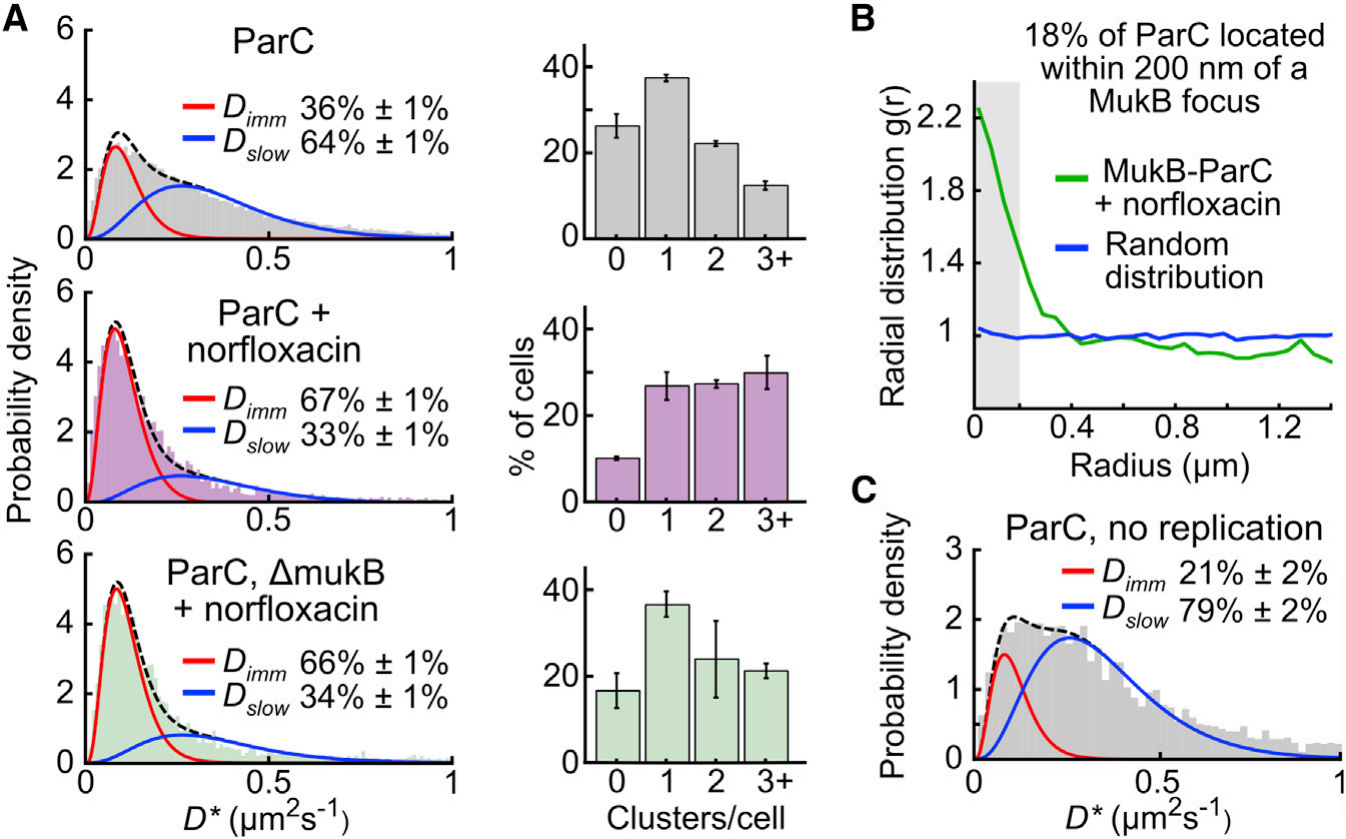
Catalytically Active TopoIV (A) Left panels: distribution of *D^∗^* values for ParC molecules in wild-type (387 cells) and Δ*mukB* (214 cells) cells after ∼10-min treatment with norfloxacin. Control ParC molecules in untreated cells (top, from Figure 1B). Right panels: number of ParC clusters per cell for steady-state populations of cells. Error bars indicate SD of three experimental repeats. Ranges give 95% confidence intervals. (B) Radial distribution of ParC localizations from each MukB focus in cells treated with norfloxacin (726 cells), compared to random distribution. (C) Distribution of *D^∗^* values for 1,930 ParC molecules in non-replicating cells, as assessed by lack of mYPet-DnaN foci prior to PALM data acquisition. Distributions of *D*^∗^ were fitted with a two-species model with both *D* values constrained.

To determine if the TopoIV-MukB interaction directs TopoIV catalytic activity close to MukBEF clusters, we treated cells carrying a norfloxacin-resistant gyrase gene with norfloxacin, which blocks the TopoIV catalytic cycle, resulting in ParC molecules covalently bound to DNA (Khodursky et al., 1995). We observed a 2-fold increase in the fraction of immobile ParC/E molecules after ∼10-min norfloxacin treatment (Figure 4A; Figure S5A), showing that most TopoIV molecules had performed catalysis during this period; however, we cannot exclude the possibility that norfloxacin captures a fraction of nonproductive catalytic events that do not result in topological changes. Longer incubation with norfloxacin did not increase the fraction of immobile molecules, showing that at ∼10 min, we had reached saturation and did therefore not have a quantitative measure of catalytic rate; shorter exposure times were not experimentally tractable. *ΔmukB* cells showed a similar fraction of immobile molecules after saturating norfloxacin treatment. This result agrees with the fact that *ΔmukB* cells can decatenate and segregate their chromosomes (Danilova et al., 2007, Nicolas et al., 2014), but it does not address the question of whether the MukB-ParC interaction stimulates decatenation globally. The enrichment of ParC molecules close to MukBEF clusters, as judged by radial distribution analysis, was retained after norfloxacin treatment (Figure 4B), showing that a fraction of TopoIV molecules underwent catalysis close to MukBEF clusters. After norfloxacin treatment of wild-type cells, we observed a modest increase in the number of TopoIV clusters per cell, whereas Δ*mukB* cells showed a similar cluster distribution to wild-type cells (Figure 4A). These data indicate that TopoIV molecules undergo catalysis in defined clusters, some of which are close to MukBEF clusters.

When we analyzed the distribution of catalytically active TopoIV molecules across the short cell axis, we found that the previous bias toward the cell center was lost, presumably because the additional clusters were not associated with MukBEF clusters (Figure S5C). MukBEF clusters were retained after norfloxacin, and ParC colocalized with them (Figure S5B). In the absence of MukBEF clusters, covalently linked DNA-ParC molecules were less likely to be located close to the long cell axis, similar to the situation in steady-state cells (compare Figure S5C curve with Figure S3A middle curve). Taken together, the results indicate that MukBEF clusters direct the catalytic activity of some TopoIV molecules to the cell long axis, whereas MukBEF-independent catalysis occurs throughout the nucleoid. In these analyses, we cannot exclude the possibility that covalently bound TopoIV reshapes the chromosome and thereby influences the spatial distribution of TopoIV. Nevertheless, the spatial distribution of MukBEF clusters was retained, suggestive of normal chromosome organization being maintained. Furthermore, we note that TopoIV-targeted strand breaks introduced by norfloxacin did not lead to chromosome fragmentation (Hsu et al., 2006).

### TopoIV Catalysis in Cells Lacking (Pre)catenanes

To address whether TopoIV catalysis occurs in cells lacking (pre)catenanes, we analyzed cells from a steady-state population that had not initiated DNA replication, as assessed by a lack of mYPet-DnaN foci. The reduction in the immobile fraction of ParC from 36% ± 1% to 21% ± 2% in these cells, when compared to the whole population (Figure 4C), indicated that almost half of immobile TopoIV molecules were dependent on replication. The replication-independent molecules showed a similar cluster distribution to that in steady-state cells (Figure S5D), consistent with a large fraction of them being bound to MukBEF clusters. Norfloxacin treatment gave a similar proportion of immobile TopoIV molecules as in steady-state cells, showing that TopoIV catalysis occurs in the absence of (pre)catenanes, but not addressing its frequency (Figure S5E). We conclude that even though the essential function of TopoIV is in decatenation, its catalytic action is not restricted to (pre)catenanes.

### The Interaction between ParC and MukB Facilitates *ori* Decatenation

To test whether the interaction between ParC and *ori*-associated MukBEF clusters influenced decatenation of newly replicated *ori*-sisters, we used two assays to assess the time of *ori* separation after replication, and we analyzed how this changed after impairment of the ParC-MukBEF interaction. These assays have been validated previously and have shown that the time of locus separation is regulated by the activity/availability of TopoIV, indicating that decatenation by TopoIV directs the time of chromosome segregation (Joshi et al., 2013, Wang et al., 2008).

**Figure 5:**
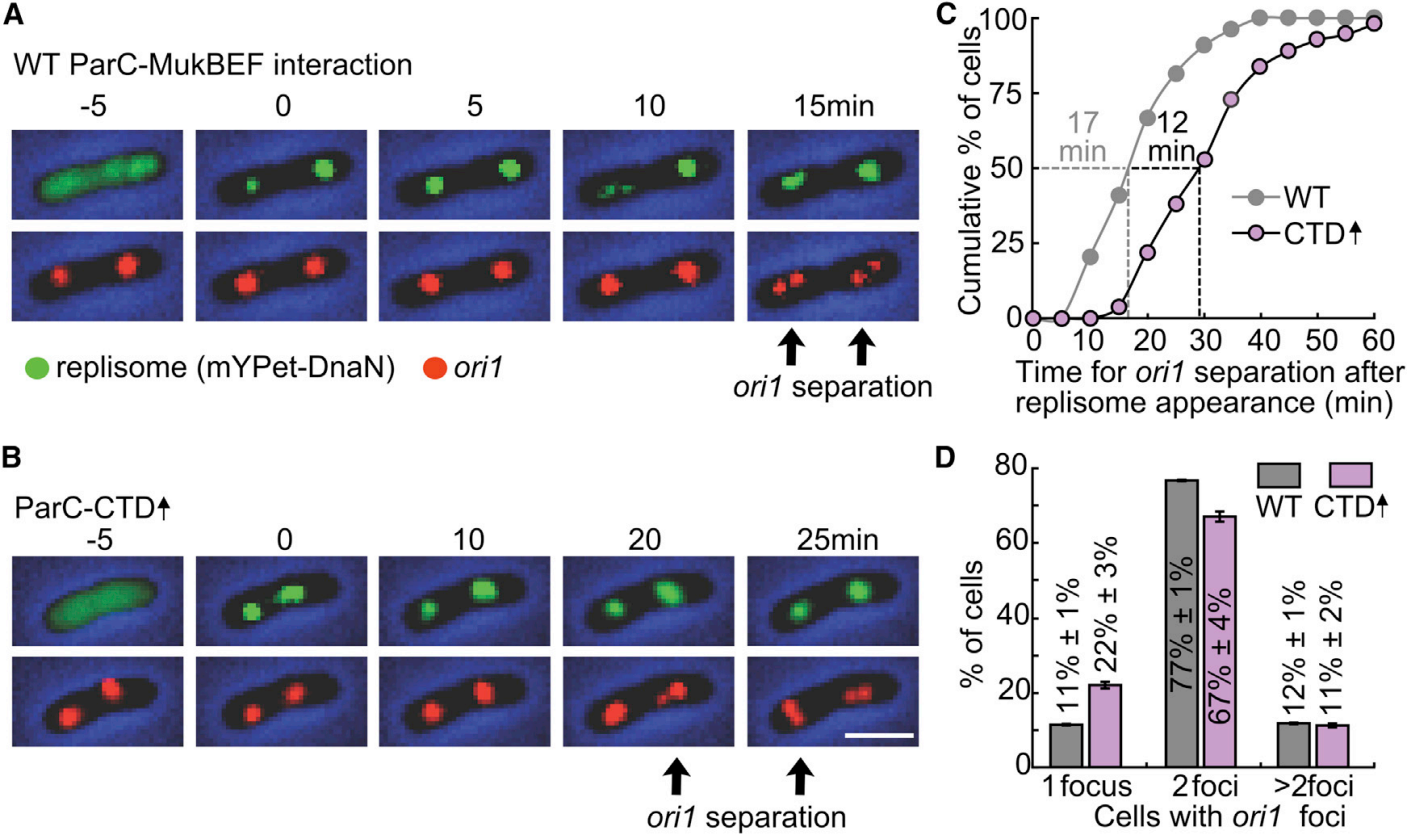
The MukB-ParC Interaction Stimulates *ori* Decatenation (A) Example cells from the time-lapse experiments with wild-type cells transformed with empty expression plasmid (pBAD24). Black arrows show time of *ori1* segregation. 0 min time was defined by replisome appearance at *ori1*. (B) Example cell showing ParC-CTD 3-hr overexpression. (C) Cumulative distribution of times of *ori1* locus segregation after replication initiation, marked by appearance of mYPet-DnaN foci at *ori1*. (D) Snapshot analysis of the number of *ori1* foci/cell in steady-state cells. Mean ± SD of three independent experiments (>1,000 cells).

In time-lapse experiments, we measured the time of *ori1* locus separation after replication initiation, marked by the appearance of a fluorescent mYPet-DnaN focus (Figure 5A). The *ori1* locus replicates <30 s after initiation at *oriC*, and a sufficient amount of mYPet-DnaN loads at the forks to visualize it within <2 min of initiation (Moolman et al., 2014). Cells in which the MukBEF-ParC interaction is normal had stably segregated 50% of the newly replicated *ori1* loci by 17 min after the appearance of mYPet-DnaN. In contrast, cells in which the MukBEF-ParC interaction was impaired by ParC-CTD overexpression showed a ∼12 min increase in the time required for 50% of cells to exhibit *ori1*-segregation (Figures 5B and 5C).

We also compared the fraction of cells containing one or more *ori1* foci in snapshots of steady-state populations (Figure 5D). We observed a decrease in the fraction of cells containing two *ori1* foci when the ParC-MukBEF interaction was impaired, consistent with delayed *ori1* decatenation. Taken together, these results provide strong support for a mechanism in which the MukB-TopoIV interaction plays a role in timely decatenation of newly replicated *ori*1 DNA.

## Discussion

The in vivo single-molecule approach exploited here provides a comprehensive understanding of the formation and behavior of TopoIV molecules in their native unperturbed environment inside living cells. We observed a dynamic equilibrium between free and complexed ParC/E subunits, independently of replication, with ∼60 potentially functional TopoIV heterotetramers at birth. The observation that only ∼60% of subunits are in heterotetramers at any given time raises the possibility that more functional enzymes can be assembled if required. By combining in vivo super-resolution techniques with genetics, inhibitors, and overexpression of competing proteins, we have gained a molecular understanding of the interaction between TopoIV and MukB and demonstrated the important functional significance of this interaction for DNA segregation. We are confident that the C-terminal fusions of ParC and ParE are fully functional, that the fluorescent protein domains themselves are not influencing the localization and diffusional properties of the fusion proteins and therefore that the observed behavior reflects the true properties of TopoIV subunits (Supplemental Experimental Procedures).

### Catalysis by TopoIV

We infer that individual TopoIV molecules undergo multiple attempts to bind DNA productively before undergoing catalysis. This is reflected in the slow diffusion of ParC/TopoIV molecules, which simulations have indicated may result from transient (≤1 ms) interactions with DNA (Figure 1B; Figure S4C). We infer that this transient binding probably does not identify molecules undergoing catalysis, since a single TopoIV catalytic cycle was measured to be ∼1 s in single-molecule and ensemble experiments in vitro (Crisona et al., 2000, Neuman et al., 2009, Stone et al., 2003). Our analysis is consistent with other data (Lee et al., 2013, Stone et al., 2003, Vos et al., 2013a) that has led to the proposal that prior to catalysis, TopoIV must first capture the G-DNA segment that is to be cleaved and then capture a T-segment that is transported through the cleaved G-segment. The transient DNA binding we observed likely represents an initial interaction with DNA proceeding G-segment capture or G-segment capture itself. Assuming that long binding events, lasting ∼1.8 s (t_catalysis_, Figure 3D), represent catalytically active molecules and that 14% (F_catalysis_) of all molecules display this behavior (Figure 3B), we calculated the mean time for a given TopoIV molecule to locate and productively bind to its substrate, t_search_, using F_catalysis_ = t_catalysis_/(t_catalysis_ + t_search_) (Uphoff et al., 2013). We calculated that (t_search_ + t_catalysis_) = ∼13 s. Therefore, for ∼11 s, TopoIV molecules will diffuse slowly, presumably undergoing multiple transient interaction with DNA, before initiating a catalytic cycle.

(−) supercoils are relaxed distributively in vitro, whereas (+) supercoils are relaxed processively (Crisona et al., 2000, Stone et al., 2003). Given that processive events would be predicted to last tens of seconds, the inferred TopoIV catalytic cycle of 1.8 s, derived from the long exposure analysis, suggests that TopoIV predominantly acts distributively rather than processively in vivo. This is consistent with RH (−) supercoils and RH replicative catenanes being the preferred targets for TopoIV.

Our results provide a deeper understanding of the relative activity of TopoIV on (pre)catenanes behind replication forks and in maintaining global supercoiling homeostasis. TopoIV-mediated decatenation behind the fork is essential, and TopoIV inactivation prevents decatenation and segregation of newly replicated sister loci without affecting replication or transcription (Wang et al., 2008). DNA gyrase is thought to be largely responsible for removal of LH (+) supercoils ahead of replication forks and the transcription machinery (Vos et al., 2011). Our results showing a reduction of immobile TopoIV molecules in non-replicating cells, and a failure to detect processive relaxation of (+) supercoils in vivo, are consistent with most TopoIV activity being directed to decatenation of newly replicated DNA. Consistent with this, covalent linking of TopoIV to DNA with norfloxacin does not block chromosomal or plasmid replication (Khodursky and Cozzarelli, 1998, Khodursky et al., 1995). In the absence of functional gyrase, TopoIV could support replication at ∼30% of the wild-type rate (Khodursky et al., 2000), indicating that TopoIV may act ahead of the fork, although it could be that in the absence of gyrase accumulation of the (+) supercoiling ahead of the fork is converted intro precatenanes by replisome rotation, thereby allowing limited fork progression as a consequence of TopoIV-mediated decatenation.

The presence of a similar proportion of TopoIV heterotetramers in cells of all ages (Figures S5F–S5H) and catalytically active TopoIV molecules in cells that have not initiated replication, along with the demonstration that TopoIV availability controls decatenation at *ori* (Figure 5) (Joshi et al., 2013, Wang et al., 2008, Nicolas et al., 2014), shows that TopoIV activity is not directed exclusively to replication termination and is inconsistent with the hypothesis that active TopoIV molecules form only at replication termination as a consequence of replisome disassembly (Espeli et al., 2003).

### MukBEF Cluster-Dependent TopoIV Molecules

Our data lead us to propose that direct interaction between ParC and MukB leads to a fraction of TopoIV molecules being bound to MukBEF clusters for 30–70 ms, unless they undergo catalysis, in which case we propose that they will remain bound for ∼1.8 s. The MukBEF cluster-ParC interaction is important for timely segregation of newly replicated sister *ori*s, consistent with the observation that TopoIV availability determines *ori1* locus separation time (Wang et al., 2008). Furthermore, ablation of TopoIV activity, prevents *ori* segregation and its reinstatement leads to resumed *ori* segregation (Nicolas et al., 2014). These observations strongly suggest that measurements of *ori* segregation time define decatenation efficiency. In the experiments here, we have demonstrated an ∼12-min delay in *ori1* segregation if the ParC-MukB interaction is impaired, consistent with TopoIV being less active in decatenation without this interaction. We propose the TopoIV interaction with MukBEF clusters may favor *ori* decatenation partly because of an increased local concentration of TopoIV and partly because of enhanced catalysis. Because the MukB-ParC interaction stimulates relaxation of RH (−) supercoils in vitro, we would also expect this interaction to stimulate decatenation because of the identical chirality of replicative catenanes and negative supercoils (Nicolas et al., 2014). Although in vitro experiments designed to test whether the TopoIV-MukB interaction stimulated decatenation showed little or no stimulation (Hayama et al., 2013, Hayama and Marians, 2010, Li et al., 2010), the substrates used were different from those used in the supercoil relaxation experiments. We propose, given our in vivo results and the identical chirality of replicative catenanes and (−) supercoils, that the TopoIV-MukBEF interaction will stimulate decatenation. Since the MukBEF clusters are relatively stably associated with DNA (Badrinarayanan et al., 2012), their interaction with TopoIV may facilitate binding of the G- and/or T-segment by TopoIV. Alternatively, this interaction might affect TopoIV substrate specificity.

Since decatenation of newly replicated *ori*s only occurs during a short period of the cell cycle, we wonder also whether the MukBEF cluster-TopoIV interaction may stimulate (−) supercoil relaxation in the region of the origin and thereby act to prevent premature *ori* firing, which requires that *ori* is highly negatively supercoiled (Donczew et al., 2014). Consistent with this, we note that MukBEF clusters tended to move away from *ori* prior to replication initiation (Nicolas et al., 2014) and that in cells in which the TopoIV-MukB interaction is perturbed, we observed some replication initiation asynchrony (Figure S1A).

### Perspective

We propose that the coordination of type II topoisomerase activity by an SMC complex revealed here is not limited to *E. coli*. Other studies have implicated functional interactions between eukaryotic SMCs and their TopoIV counterpart, TopoII (Baxter, 2015, Baxter and Aragón, 2012). For example, condensin was shown to facilitate decatenation of yeast minichromosomes (Charbin et al., 2014). The sequential and coordinated action of TopoIV and MukBEF in the successive steps of decatenation and chromosome segregation revealed here provides a platform for future mechanistic studies that will reveal whether SMC complexes provide DNA-protein substrates that provide selectivity for topoisomerase action and precisely how topoisomerase action is coordinated with SMC functions in chromosome processing.

## Experimental Procedures

### Bacterial Strains and Growth

Bacterial strains are listed in Table S1. Plasmids and oligonucleotides are shown in Table S2. Strains were streaked onto Luria-Bertani broth plates with appropriate antibiotics. Single colonies were inoculated into M9 glycerol (0.2%) and grown overnight at 37°C to A_600_ 0.4–0.6, then diluted into fresh M9 and grown to A_600_ 0.1. Cells were centrifuged and immobilized on agarose pads between two glass coverslips (0.17 mm thick, heated to 500°C for 1 hr to remove any fluorescent background particles). We prepared 1% agarose pads by mixing low-fluorescence 2% agarose (Bio-Rad) in dH_2_O 1:1 with 2× growth medium. For details, see Supplemental Experimental Procedures.

### PALM Imaging, Molecule Localization, Tracking, and Diffusion

Live cell single-molecule-tracking PALM used a custom-built total internal reflection fluorescence microscope. Photoactivatable mCherry activation used a 405-nm laser, with excitation at 561 nm. YPet excitation was with a 488 nm laser. Bright-field cell images were recorded with an LED source and condenser (ASI Imaging). PALM single-molecule-tracking analysis used custom-written MATLAB software (MathWorks). We distinguished bound and diffusing proteins by calculating an apparent diffusion coefficient *D^∗^* = MSD/(4 Δt) from the mean-squared displacement (MSD) for each track with four steps. Note that *D^∗^* is an apparent diffusion coefficient because of cell confinement and motion blurring (Stracy et al., 2014). For details, see Supplemental Experimental Procedures.

### Measuring Long-Lasting Binding Events

PALM movies to measure long-duration binding events were recorded at low continuous 561-nm excitation intensities using long exposure times (Uphoff et al., 2013). At these exposure times, mobile ParC-PAmCherry molecules are motion blurred over a large fraction of the cell, whereas immobile ParC-PAmCherry molecules still appear as point sources, producing a diffraction limited spot. The probability of observing a particular on-time is the product of the underlying binding-time probability and the bleaching probability. The bleaching-time distributions were measured independently using MukB-PAmCherry, which binds DNA in one to three large clusters per cell with a dwell time of ∼50 s (Badrinarayanan et al., 2012), with the same acquisition and excitation conditions. On-time and bleaching-time distributions were fitted with single-exponential functions to extract exponential-time constants t_on_ and t_bleach_, and the binding-time constant was calculated by t_bound_ = t_on_ × t_bleach_ / (t_bleach_ − t_on_).

### Simulations

Diffusion simulations were performed with custom-written MATLAB software (MathWorks). Molecules were simulated undergoing Brownian motion confined within a volume corresponding to the average size of cells imaged in experiments. The localization in each 15-ms frame determined from averaging the simulated molecule positions over 100 subframes and adding Gaussian distributed localization error. The list of simulated localizations, with their corresponding frame number, could then be analyzed in exactly the same way as the experimental data.

### Measuring Cohesion Time

Sister *ori1* cohesion time in the strain KG52 containing plasmid pZ68 (overproducing a ParC CTD domain) was assessed in a 5-min time-lapse analysis. We have measured the time from replisome appearance at initiation to *ori1* segregation. Chromosomal genetic loci were visualized using fluorescent repressor-operator systems. A *lacO* array was inserted 16 kb counterclockwise of oriC (*ori1)*; LacI-mCherry was expressed from the chromosomal *leuB* locus, regulated by the *lac* promoter (Wang et al., 2008). A chromosomally encoded mYPet-DnaN fusion protein was used as a marker for the replisome (Moolman et al., 2014, Reyes-Lamothe et al., 2010). Cells were growing exponentially in minimal medium supplemented with glycerol, at 37°C (generation time ∼100 min). CTD overproduction was induced by addition of L-arabinose (final concentration, 0.2%) 3 hr prior to the experiment. As a control, the strain with the empty plasmid pBAD24 (Guzman et al., 1995) was used.

## Author Contributions

P.Z. and D.J.S. designed the research. P.Z., K.G., K.Z., and C.L. performed experiments and analyzed data. M.S. wrote analytical tools and analyzed data. A.N.K provided technical advice. P.Z., M.S., and D.J.S. wrote the paper.

## Supplemental Information

Document S1. Supplemental Experimental Procedures, Figures S1–S5, and Tables S1–S4

Movie S1. Visualization of 3D Structured Illumination Images Showing the Organization of MukB Foci Inside Living Cells, Related to Figures 2 and S3 MukB-mYPet fluorescence is shown in green, and DAPI-stained DNA is shown in red.

Document S2. Article plus Supplemental Information
