# Supplementary material for: The Localization and Action of Topoisomerase IV in Escherichia coli Chromosome Segregation Is Coordinated by the SMC Complex, MukBEF
Source: Cell Rep. 2015 Dec 10;13(11):2587–96. doi: 10.1016/j.celrep.2015.11.034 (PMC5061553; doi:10.1016/j.celrep.2015.11.034)
Supplement: Document S1. Supplemental Experimental Procedures, Figures S1–S5, and Tables S1–S4 [file mmc1.pdf]

Cell Reports
Supplemental Information

# The Localization and Action of Topoisomerase IV in *Escherichia coli* Chromosome Segregation Is Coordinated by the SMC Complex, MukBEF

Pawel Zawadzki, Mathew Stracy, Katarzyna Ginda, Katarzyna Zawadzka, Christian Lesterlin, Achillefs N. Kapanidis, and David J. Sherratt

**The Localization and Action of Topoisomerase IV in *Escherichia coli* Chromosome Segregation is Coordinated by the SMC Complex, MukBEF**

**Authors:**

Pawel Zawadzki[1,4], Mathew Stracy[2,4], Katarzyna Ginda[1], Katarzyna Zawadzka[1], Christian Lesterlin[1,3], Achillefs N. Kapanidis[2], David J. Sherratt[1*]

**Affiliations:**

[1]Department of Biochemistry, University of Oxford, South Parks Road, Oxford, OX1 3QU, UK.

[2]Biological Physics Research Group, Clarendon Laboratory, Department of Physics, University of Oxford, Parks Road, Oxford, OX1 3PU, UK.

[3]Bases Moleculaires et Structurales des Systemes Infectieux, UMR 5086, Centre National de la Recherche Scientifique, University of Lyon, 69367 Lyon, France

[4]Co-first author

Corresponding author: D.J. Sherratt, Department of Biochemistry, University of Oxford, South Parks Rd, Oxford, OX1 3QU, UK Tel: +44 (0)1865 613237 Fax: +44 (0)1865 613238 david.sherratt@bioch.ox.ac.uk

## Supplemental Experimental Procedures

**1. Bacterial Strains and Cell Preparation.**

All strains were derivatives of *Escherichia coli* K-12 AB1157 (Bachmann, 1972) and are listed in Table S1. The plasmids and oligonucleotides used are shown in Table S2. Fusion of genes with fluorescent tags used λRed recombination (Datsenko and Wanner, 2000). Fused genes were transferred to generate the final strains through P1 phage transduction (Thomason et al., 2007). For multiple insertions of modified genes, the $Kan^r$ gene was removed using site-specific recombination through expression of the Flp recombinase from plasmid pCP20 (Datsenko and Wanner, 2000). *lacO* and *tetO* arrays were inserted 16 kb CCW of *oriC* (*ori1*) and 50 kb CW of *dif* (*ter3*), respectively (Badrinarayanan et al., 2012). Justifiably, concerns have been raised about the interpretation of results form experiments using fluorescent fusions because the fluorescent protein may interfere with function of the protein it is tagged to, and/or the fluorescent tag itself may influence the localization and apparent copy number of the protein under study (Landgraf et al., 2012; Wang et al., 2014). We are confident that our data and interpretations are physiologically relevant and are free from artefacts. For example, fusions of PAmCherry and mYPet to any of the components of TopoIV or MukBEF, or to DnaN in the case of mYPet, gave functional fusions when expressed from the endogenous chromosomal context in the absence of wild type protein. Fusions to a given protein showed similar cellular behavior independently of whether the imaging used PALM, widefield, or SIM. Furthermore, fusions to different proteins behaved in a protein-specific manner that reflected the known biology of the proteins. Growth rates, cell size distributions and flow cytometry profiles were identical to those of wild type cells (Table S3; Figure S1A). Finally, the cellular behavior of ParC-PAmCherry expressing cells changed predictably when its interaction with MukBEF clusters was impaired by deleting MukB or by over-expressing ParC-CTD or when norfloxacin was used to covalently link TopoIV to DNA.

Strains were streaked onto LB plates with appropriate antibiotics. Single colonies were inoculated into M9 glycerol (0.2%) and grown overnight at 37ºC to $A_{600}$ 0.4-0.6, then diluted into fresh M9 and grown to $A_{600}$ 0.1. Cells were centrifuged and immobilized on agarose pads between two glass coverslips (0.17mm thickness, heated to 500ºC for 1 h to remove any fluorescent background particles). We prepared 1% agarose pads by mixing low-fluorescence 2% agarose (Bio-Rad) in $dH_2O$ 1:1 with 2x growth medium. For fixation, centrifuged cells prepared as above were resuspended into 2.5% paraformaldehyde in PBS buffer and fixed for 45 min shaking at 22ºC. Fixed cells were washed with PBS and immobilized on agarose pads as above. Nucleoids were visualized using 5 μg $ml^{-1}$ 4′,6-diamidino-2-phenylindole (DAPI). Norfloxacin was used at 5 μg $ml^{-1}$. Cells were placed on an agarose pad containing the drug 5 minutes prior the experiment.

## 2. Epifluorescence Microscopy

Conventional wide-field fluorescence microscopy used an Eclipse TE2000-U microscope (Nikon), equipped with an 100x/NA1.4 oil PlanApo objective and a Cool-Snap $HQ^2$ CCD, and using Metamorph software for image acquisition.

Sister *ori1* cohesion time in the strain KG52 containing plasmid pZ68 (overproducing a ParC CTD domain) was assessed in a 5-min time-lapse analysis. We have measured the time from replisome appearance at initiation to *ori1* segregation. Chromosomal genetic loci were visualized using fluorescent repressor–operator systems. A *lacO* array of 240 copies was inserted 16 kb CCW of oriC (*ori1)*; LacI-mCherry was expressed from the chromosomal *leuB* locus, regulated by the *lac* promoter (Wang et al., 2008). A chromosomally encoded mYPet-DnaN fusion protein was used as a marker for the replisome (Moolman et al., 2014; Reyes-Lamothe et al., 2010). Cells were growing exponentially in minimal medium supplemented with glycerol, at 37°C (generation time ~100 min). CTD overproduction was induced by addition of L-Arabinose, final concentration 0.2%, 3 hours prior to the experiment. As a control, the strain with the empty plasmid, pBAD24 (Guzman et al., 1995) was used.

## 3. 3D Structured-Illumination Microscopy

Super-resolution 3D-SIM imaging was performed as in (Lesterlin et al., 2014), on a DeltaVision OMX V3 (Applied Precision/GE Healthcare) equipped with a Blaze SIM module, a ×60/1.42 oil UPlanSApo objective (Olympus), 405 nm and 488 nm diode

lasers and three sCMOS cameras (PCO). Cells were grown in LB in order to obtain more discrete lobed nucleoids. Three-dimensional two-color stacks of MukB-mYPet and DAPI stained DNA were obtained using sequential acquisition of 512 × 512 pixels image stacks with 8 x 125 nm z-sections (sample thickness 0.75 μm). Each z-section results from striped illumination patterns rotated to the three angles (−60°, 0°, +60°) and shifted in five phase steps. Acquisition settings were as follows: MukB–mYPet, 30 ms exposure with 488 nm laser (100 % transmission) and DAPI, 15 ms exposure with 405 nm laser (100% transmission). 3D-SIM raw data were computationally reconstructed with SoftWoRx 6.0 (Applied Precision) using a Wiener filter setting of 0.002 and channel specifically measured optical transfer functions to generate a super-resolution three-dimensional image stack. Images from the different channels were aligned using parameters obtained from calibration measurements with 0.2-μm-diameter TetraSpeck beads (Life Technologies) using the OMX Editor software (C. Weisiger and J. Sedat, unpublished). IMARIS analysis software (BITPLANE) was used to generate 3D rendering of fluorescent signals (Figure S3C; Movie 1), with bulk nucleoid DNA represented by a red wireframe, which excludes fluorescence values that are below the threshold of 20% of the maximum intensity value on the raw DAPI image. This results in exclusion of background fluorescence and the 'cloudy' fluorescence signal at the periphery of the nucleoid. To identify regions of highest DNA density (solid red) surfaces including regions with fluorescence values above the exclusion threshold of 70% of the maximum DAPI intensity values were generated. The distance between MukBEF clusters and the highest DNA density zone of the nucleoid was extracted from z-projections of the 3D-SIM stack to obtain 2D images. The *x* and *y* coordinates of the maximum intensity pixels for MukB and DAPI signals were identified using the FIJI "*find maxima*" function. The distance between the MukBEF maximum intensity pixel and the closest DAPI maximum intensity pixel was then calculated. Pixels were converted to distances using 0.082 μm/pixel. The mean distance between the brightest DAPI pixel and the brightest MukB-mYPet pixel (0.22 μm ± 0.12 μm) was derived from 97 distances, measured from four fields of view in two independent experiments.

### 4. PALM Microscopy

Live cell single-molecule-tracking PALM was performed on a custom-built total internal reflection fluorescence (TIRF) microscope built around the Rapid Automated

Modular Microscope (RAMM) System (ASI Imaging). Photoactivatable mCherry activation was controlled by a 405 nm laser and excitation with 561 nm. GFP and YFP excitation was provided by a 488 nm laser. All lasers provided by a multi-laser engine (iChrome MLE, Toptica). At the fiber output, the laser beams were collimated and focused (100x oil immersion objective, NA 1.4, Olympus) onto the sample under an angle allowing for highly inclined thin illumination (Tokunaga et al., 2008). Fluorescence emission was filtered by a dichroic mirror and notch filter (ZT405/488/561rpc & ZET405/488/561NF, Chroma). PAmCherry emission was projected onto an EMCCD camera (iXon Ultra, 512x512 pixels, Andor). The pixel size was 96 nm. Brightfield cell images were recorded with an LED source and condenser (ASI Imaging). Sample position and focus were controlled with a motorized piezo stage, a z-motor objective mount, and autofocus system (MS-2000, PZ-2000FT, CRISP, ASI Imaging).

**5. Localization and Tracking**.

PALM data for single-molecule-tracking analysis was localized using custom-written MATLAB software (MathWorks). Fluorophore images were identified for localization by band-pass filtering and applying an intensity threshold to each frame of a super-resolution movie. Candidate positions were used as initial guesses in a two-dimensional elliptical Gaussian fit for high-precision localization. Free fit parameters were x-position, y-position, x-width, y-width, elliptical rotation angle, intensity, background. Single-particle tracking analysis was performed by adapting the MATLAB implementation of the algorithm described in (Crocker and Grier, 1996). Positions were linked to a track if they appeared in consecutive frames within a window of 5 pixels (0.48 μm). In rare cases when multiple localizations fell within the tracking radius, tracks were linked such that the sum of step distances was minimized. We used a memory parameter of 1 frame to allow for transient (1 frame) disappearance of the fluorophore image within a track due to blinking or missed localization.

**6. Molecule Counting**

We counted the total number of ParC or ParE molecules by recording long movies (61000 frames) – until no further activation was observed. Copy numbers were derived from observed counts of molecules per cell. The experimentally determined

number of photoactivatable molecules likely underestimates the real copy number due to the fact that not all PAmCherry proteins can be activated (Durisic et al., 2014). In this study, using Xenopus oocytes, ~50% of PAmCherry molecules could be activated, with ~85% of the molecules not blinking. We do not have the appropriate correction factor for *E. coli*, but think it likely that we underestimate the copy number by a factor of ~2. All cells in the steady state population were analyzed, then the copy number was normalized to a 2.5µm long cell (the length soon after birth). Additionally, cells were placed on an agarose pad with norfloxacin (5µg/ml) in order to increase the fraction of immobile molecules. To estimate the number of TopoIV heterotetramers we used data from Figure 1C. There are 210 ParE molecules per cell; 32% are immobile and 24% are slowly diffusing. Therefore, 56% of 210 ParE molecules (117) are complexed with ParC, giving ~60 $ParC_2ParE_2$ TopoIV heterotetramers present at any time.

To estimate the number of TopoIV molecules associated with MukBEF clusters we used two calculations derived using different datasets. First, given that 14% of ~210 ParE molecules (~30) were dependent on MukB and immobile (and therefore in TopoIV heterotetramers), we deduced that ~15 TopoIV molecules were MukBEF cluster-associated at any time. Second, we used the 20% of 289 ParC molecules that were close to MukBEF clusters in the radial distribution analysis (Figure 2B; ~58 molecules). Since ~42% of all ParC molecules overall were in TopoIV heterotetramers (compare Figure 1B and 1C), we get a value of ~12 TopoIV molecules associated with MukBEF clusters. In contrast, if we compare Figure 2A top and middle panels, we get 16% of ~289 ParC molecules being immobile and dependent on MukB (~46). Since we deduce above that ~30 TopoIV heterotetramers are immobile and dependent on MukB, then we deduce that 65% of ParC molecules are in MukB-dependent TopoIV heterotetramers. 65% of the ~58 ParC molecules close to clusters gives ~19 TopoIV heterotetamers associated with MukBEF clusters. We conclude therefore that 12-19 TopoIV molecules were MukBEF-cluster associated at any time and in the main text, we use the value of ~15, which is the value from the first method.

**7. Measuring the Diffusion of PAmCherry Labeled Proteins**.

We determined the mobility of each molecule by calculating an apparent diffusion coefficient from the one-step mean-squared displacement (MSD) of the track using:

**Equation 1**

$$D^* = \frac{1}{4n\Delta t}\sum_{i=1}^{n}[x(i\Delta t) - x(i\Delta t + \Delta t)]^2 + [y(i\Delta t) - y(i\Delta t + \Delta t)]^2$$

Where $x(t)$ and $y(t)$ are the coordinates of the molecule at time $t$, the frame time of the camera is $\Delta t$, and $n$ is the number of steps in the trajectory. Tracks shorter than $n = 4$ steps long were discarded for this analysis because the higher uncertainty in *D** value.

For a molecule with diffusion coefficient D, the probability distribution of obtaining a D* value, $x$ , is given by:

**Equation 2**

$$f(x; D, n) = \frac{({}^{n}/_{D})^{n} x^{n-1} e^{-nx/D}}{(n-1)!}$$

Where $n$ is the number of displacement steps in the trajectory. To allow for fitting to the $D^*$ distribution longer tracks were truncated after 5th localization (i.e. $n = 4$), and the $n = 4$ equation was used:

**Equation 3**

$$f(x; D) = \frac{({}^{4}/_{D})^{4} x^{3} e^{-4x/D}}{6}$$

Least squares fitting to the histogram of *D** values was used. A single species model fits poorly to the data (Figure S1B). We reasoned that at least two species with different mobilities are present: mobile molecules diffusing and transiently binding DNA, and immboile molecules bound to DNA. We therefore introduced a second species:

**Equation 4**

$$f(x; D_1, D_2, A_1, A_2) = \frac{A_1({}^{4}/_{D_1})^{4} x^{3} e^{-4x/D_1}}{6} + \frac{A_2({}^{4}/_{D_2})^{4} x^{3} e^{-4x/D_2}}{6}$$

Where $D_1$ and $D_2$ are the diffusion coefficients of the two different species, and $A_1$and $A_2$ are the fraction of molecules found in each state, and $A_1 + A_2 = 1$. For fitting to ParE a third species was added in a similar fashion.

We note that Equations 1-3 assume that the *n* displacements averaged to generate the *D** value are independent, which is not strictly true as the same localisation is used to determine the preceeding and following displacement step, and hence any localisation error and motion blurring of this localisation will effect both displacements (Michalet, X. 2010). To verify that Equations 1-3 are adequate approximations for *D** values generated from overlapping displacements we used ParC trajectories with 7 displacements and determined the *D** value from either the first 4 sequential steps (using Equation 1, with *n* = 4) or from nonoverlapping steps using:

**Equation 5**

$$D^* = \frac{1}{16\Delta t}\sum_{i=1}^{4}[x((2i-1)\Delta t) - x(2i\Delta t)]^2 + [y((2i-1)\Delta t) - y(2i\Delta t)]^2$$

The *D** distribution from sequential steps was essentially identical to the distribution generated with nonoverlapping steps and fitting both with Equation 4 gave identical values (see Fig. S1 J).

To establish the apparent diffusion of the DNA-bound species, we then turned to a well charaterized control protein DNA polymerase 1 (Pol1) which shows clearly distinct *D** populations for molecules specifically bound to DNA and those mobile molecules diffusing through the nucleoid searching the for substrate (Fig. S1C). Inducing DNA methylation damage by incubating cells with MMS increases the fraction of specifically bound molecules (described in (Uphoff et al., 2013)), making it easier to resolve the distribution of bound molecules. Fitting this distribution to two diffusing species allows us to determine the *D* value of specifically bound molecules, as *D** = 0.11 $\mu m^2 s^{-1}$. This apparent motion of bound molecules is mainly due to localisation error, which manifests as a shift to the right in *D** value of $\sigma_{loc}^2$ /Δt, hence immobile molecules appear to have a *D** value of ~ 0.1 $\mu m^2 s^{-1}$. Using this D* value

for bound molecules to constrain one $D^*$ species and allowing a second unconstrained $D^*$ species fits well to the data giving two populations of immobile molecules, $D_{imm}$ = 0.11 μm$^2$s$^{-1}$, and molecules slowly diffusing, $D_{slow}$ = 0.35 μm$^2$s$^{-1}$ (Fig.1B).

ParE $D^*$ histograms, on the other hand, fits poorly to a two species distribution. Instead, we reasoned that third population with faster diffusion exists, representing uncomplexed ParE molecules which do not bind DNA. Consistent with this overexpression of unlabelled ParE resulted in the dramatic increase in fraction of this third population showing that this population represents individual ParE subunits not nomplexed with ParC (Figure S2G and H). Fiting three diffusing species to overexpression data with two population constrained at values obtained for ParC; $D_{imm}$ =0.11μm$^2$s$^{-1}$, and $D_{slow}$ =0.35 μm$^2$s$^{-1}$, resulted in a good fit to the data, with a third population at $D_{fast}$ =0.94 μm$^2$s$^{-1}$ (Figure S1H). Subsequently, we fit ParE distribution with three species and constrained all three $D$ values at; $D_{imm}$ =0.11 μm$^2$s$^{-1}$, $D_{slow}$ =0.35 μm$^2$s$^{-1}$ and $D_{fast}$ =0.94 μm$^2$s$^{-1}$.

**8. Intracellular Spatial Distributions of Classified Molecules**

A two species fit to the ParC gave 36 % as immobile and 64 % mobile. We determined a $D^*$ threshold (0.16 μm$^2$s$^{-1}$) which preserves the ratio of immobile-to-mobile molecules established from fitting to the population to sort each individual ParC track as immobile or mobile based on its mobility (Stracy et al., 2015; Uphoff et al., 2013). In order to minimize the uncertainty when determining the $D^*$ for categorizing individual molecules, only trajectories with at least 4 steps were used, and all steps longer than 4 were included. Based on the overlap between the two fitted distributions truncated at 4 steps, we estimate that this threshold correctly categorized 86 % of molecules. This is likely to be an underestimate of the true categorization accuracy as the variance in the distributions of longer trajectories is smaller thus the overlap is reduced.

Using this threshold we determined the average spatial distribution of immobile and mobile ParC molecules over many cells. To do this, cells were segmented from brightfield images using MicrobeTracker (Sliusarenko et al., 2011). The probability distributions along the cell short-axis were calculated by determining the distance of each localization from the cell midline, with the distances normalized

to 1 and -1 at the cell membrane and 0 at the cell midline. The analytical probability distribution for a uniform distribution within a cylindrical volume is also presented (Sanamrad et al., 2014) is given by:

$$f(\rho) = \frac{2\sqrt{\max(1-\rho^2, 0)}}{\pi}$$

Where $\rho$ is the relative short-axis position. This is the expected distribution from a molecule able to move freely through the complete cell volume, and is shown in Figure S1G for comparison with experimental distributions.

**9. Clustering Analysis and Radial Distribution Analysis**

The radial distribution functions and clustering analysis were performed in Matlab (Mathworks). The radial distribution function, g(*r*), was calculated for all ParC/E localizations inside segmented cells. First, the pairwise distances of all localizations were calculated on a cell by cell basis. Because of the small size of *E. coli*, as the radius, *r*, of the pairwise distance increases much of the area falls outside the cell. To account for this effect we simulated the randomly distributed points within the same segmented cell outline. For each segmented cell the same number of localizations was simulated as observed experimentally, and their pairwise distances calculated. This was repeated over all cells and the histogram of pairwise distances was divided by the simulated random pairwise distances to give the empirical radial distribution function, g(r), with random distribution having a g(*r*) of one.

A nearest neighbor clustering algorithm was implemented to cluster ParC localizations on a cell by cell basis inside cells. Localizations were counted as a cluster if more than 25 localizations clusters using a nearest neighbor threshold of 200 nm. The number of clusters per cell was recorded. Cells with fewer than 300 localizations were not used for this analysis.

**10. Measuring Long-lasting Binding Events**

PALM movies to measure long duration binding events were recorded at low continuous 561-nm excitation intensities using long exposure times (500 ms/frame, 750 ms/frame, and 1000 ms/frame) (Uphoff et al., 2013). At these exposure times mobile ParC-PAmCherry molecules are motion blurred over a large fraction of the

cell, whereas immobile ParC-PAmCherry molecules still appear as point sources, producing a diffraction limited spot. Elliptical Gaussian fitting was used as described in section S5. Bound and mobile molecules were distinguished by the width of the elliptical fits, with thresholds short axis-width < 160 nm and long axis-width < 200 nm to identify bound molecules (Figure S4E). The probability of observing a particular on-time is the product of the underlying binding-time probability and the bleaching probability. The bleaching-time distributions were measured independently using MukB-PAmCherry, which binds DNA in 1-3 large clusters per cell with a dwell time of ~50s (Badrinarayanan et al., 2012), with the same acquisition and excitation conditions. On-time and bleaching-time distributions were fitted with single-exponential functions to extract exponential-time constants $t_{on}$ and $t_{bleach}$, and the binding-time constant was calculated by $t_{bound} = t_{on} \cdot t_{bleach} / (t_{bleach} - t_{on})$. Stochastic photoactivation of ParC-PAmCherry molecules before or during binding events does not influence our measurement, because the observed binding times follow an exponential distribution and are therefore memoryless. All three time regimes (500, 750 and 1000ms) gave similar binding times.

This analysis showed that most binding events are not long (>30 s) processive events. Nevertheless, the calculated binding time of 1.8 s ± 0.4 s assumes a single binding dwell time, so we cannot rule out a mixed population of catalytic events, with a majority of short (>1 s) and a minority of long-lived (>30 s) binding events. We therefore undertook a time-lapse experiment to increase fluorophore lifetime, by using 4 s delays between 1 s exposures. This analysis gave a binding time of 2.6 s ± 0.7 s (Figure S4F) indicating that there may be some longer binding events.

To estimate an upper limit for the fraction of longer events, which we propose would represent processive events lasting >30 s, based on analysis *in vitro* (Crisona et al., 2000; Stone et al., 2003), we undertook simulations that assumed a lower limit of 1 s for short (distributive) and 30 s for long (processive) events. 100,000 exponentially distributed dwell times were simulated with either 1s or 30 s dwells. The dwell times were truncated by the exponentially distributed bleaching times from an experimental MukB control at either 1 s exposures, or for 1 s + 4 s time-lapse. Corrected binding times were calculated for both conditions with varying fractions of 30 s events between 0% and 100 % to establish which fraction best matched the 1.8 s and 2.6 s binding times established from the experimental data. The closest match

was 22% long events. Because 1 s binding time is likely to be a lower limit for the distributive events, this indicates that ~22% is the upper limit for the fraction of long, and therefore presumably processive, catalytic events.

**11. Determining the Diffusion Coefficients of Free and Immobile TopoIV**

The free diffusion coefficient for TopoIV heterotetramers, $D_{free}$, was estimated by first establishing the diffusion coefficient for free ParE. The ParE subunit cannot bind DNA (Lee et al., 2013). Using the ParE-PAmCherry with overexpressed unlabeled ParE (which outcompetes ParE-PAmCherry in TopoIV molecules, Figure S1F) gave a clear distribution of molecules with much higher mobility, $D_{fast}$ = 0.94 $\mu m^2 s^{-1}$. We verified that these mobile ParE subunits are not interacting with DNA and therefore occupy the full volume of the cell cytoplasm by plotting their probability density of molecules across the short axis of the cell (Figure S1G), which matched very well to the distribution expected from uniformly distributed molecules within a cylindrical volume of the same dimensions as a bacterial cell (Sanamrad et al., 2014).

The *D* value of mobile ParE therefore reports on the free diffusion coefficient. However, the apparent diffusion determined experimentally through particle tracking does not take into account confinement due to the small size of bacteria, and other effects such as localization error and motion blurring (Stracy et al., 2014; Uphoff et al., 2013; Uphoff et al., 2014). To determine the accurate $D_{free}$ value we simulated Brownian motion confined within a volume corresponding to the average size of cells imaged in experiments, defined as a cylindrical volume of length 2 μm long and 0.9 μm wide with hemispherical endcaps with a radius of 0.9 μm (Uphoff et al., 2013). Each 15 ms frame was split into 100 sub-frames with Gaussian distributed displacements in each sub-frame. Each molecule trajectory was given a random starting time to mimic stochastic photoactivation. The trajectory was then simulated until photobleaching with duration sampled from an exponential distribution with a mean time equal to our experimentally determined photobleaching lifetime (~70 ms). The sub-frame distributions were then averaged to give a position for each frame, and a localization error sampled from a Guassian distribution with $\sigma_{loc}$ = 40 nm (determined from experiments in fixed cells) was added. The list of simulated localizations, with their corresponding frame number was then analyzed using the same tracking algorithm with the same settings as used for the experimental data.

The outputted tracks could then be analyzed in exactly the same way as experimental data.

Running simulations with different $D$ values between 0 and 5 $\mu m^2 s^{-1}$, we found that the $D_{fast}$ = 0.94 $\mu m^2 s^{-1}$ (Figure S4B; green line) for ParE was best matched by a simulated $D_{free}$ = 2.4 $\mu m^2 s^{-1}$. ParE and ParC subunits are similar in size (ParC = 84kDa, ParE = 70kDa). The TopoIV heterotetramer contains 2 ParE and 2 ParC molecules. Given this, we estimated that the TopoIV heterotetramer had ~4 x the volume of ParE, and hence a radius ~1.59 times as large. Because the diffusion coefficient for a spherical particle depends linearly on the radius, we therefore estimate that the $D_{free}$ for TopoIV is 2.4/1.59 = 1.51 $\mu m^2 s^{-1}$.

The movement of DNA loci is small relative to the movement of diffusing TopoIV molecules (Elmore et al., 2005). The majority of the experimentally observed $D$ = 0.11 $\mu m^2 s^{-1}$ value for immobile molecules (bound to DNA) was due to apparent motion from the localization error (determined from fixed cells). A very small $D$ motion of 0.06 $\mu m^2 s^{-1}$ on top of the localization error best matched the observed $D$ = 0.11 $\mu m^2 s^{-1}$ in live cells (Figure S4B; red line).

**12. Simulating Molecules Interconverting between Diffusive States.**

We hypothesized that our observed slow diffusion, $D_{slow}$ = 0.35 $\mu m^2 s^{-1}$ for mobile ParC molecules was due to ParC molecules interconverting between the $D_{free}$ and $D_{imm}$ states. Simulations showed that observed apparent $D_{slow}$ = 0.35 $\mu m^2 s^{-1}$ corresponds to accurate $D$ = 0.4 $\mu m^2 s^{-1,}$ which is significantly lower than $D$ = 1.51 $\mu m^2 s^{-1}$, estimated from the size of TopoIV. Having established an estimate of these values we determined the fraction of these molecules spent in each state $f_{free}$ and $f_{imm}$ using:

$$D_{slow} = (1 - f_{free})*D_{imm} + f_{free}*D_{free}$$

Solving these equation gave $f_{free}$ = 0.27 and $f_{imm}$ = 0.73. To establish if we could recapitulate our experimental data with these fractions, we simulated molecules, which could rapidly interconvert the two states; $D_{free}$ and $D_{bound}$, while spending 27% of time freely diffusing and 73% as bound. The duration of this transient binding, $t_{imm}$, was randomly sampled from an exponential with a mean of 1ms. The simulated

interconverting species (Figure.S4C) shows that the experimentally observed *D* can be recapitulated using two states; $D_{free}$ and $D_{bound}$. As $t_{imm}$ was (1ms being an upper limit) << observation time, the interconverting species appeared as a single diffusing species with reduced mobility, and could be fitted with an analytical equations for a single diffusing species at $D = 0.35\ \mu m^2 s^{-1}$.

In time lapse experiments with a 15 ms exposures followed by a 35 ms delay, a reduction in the fraction of molecules that remained immobile over the duration of the track was observed which was not seen in Δ*mukB* cells. Using 15ms exposures in Muk+ cells, the fraction of bound TopoIV is significantly higher than in Δ*mukB* cells (36%). However, in time-lapse experiments this bound fraction was reduced to the same level as Δ*mukB* cells (14%), indicating that there is a population of Muk-dependent TopoIV molecules which appear immobile over 15ms exposures, but appear mobile in time-lapse experiments. To determine the duration of the MukB dependent events we simulated molecules interconverting between $D_{imm}$ and $D_{free}$ and varied the mean duration of the binding events. Molecule trajectories were simulated and averaged to generate localizations at either 15 ms intervals or 15 ms intervals with 35ms delays to match normal and time-lapse experiments. The simulated localizations were analyzed with the same tracking and categorizing algorithm as used for experimental data. Plotting the reduction in the fraction of molecules categorized as bound in time-lapse simulations compared to normal simulations showed that binding for 30–70 ms recapitulated experimentally observed decrease (Figure S4D). For binding times significantly smaller than the exposure time of each frame the normal and time-lapse simulations showed little difference. In Δ*mukB* cells no decrease was observed between time-lapse and normal experiments. However, the observed *D* in Δ*mukB* cells was again = $0.35\ \mu m^2 s^{-1}$, suggesting that transient interactions with DNA, estimated to last ~1ms, are MukB-independent.

### 13. Flow Cytometry

Cells were grown in M9-gly to exponential phase ($A_{600} \approx 0.2$). 200 µl of the cultures were fixed with 3.5 ml of ice-cold 74% ethanol. For the staining procedure, the cells were pelleted and washed twice in 100 µl of cold staining buffer (10 mM Tris pH 7.4, 10 mM $MgCl_2$). The 100 µl samples were mixed with an equal volume of the staining

solution Syto-16 (3 μM). 100,000 events were recorded in a Becton Dickinson FACScalibur machine using FL1-H and results were analysed using FlowJo.

**14. Determination of *in vivo* Dissociation Constants**

At equilibrium, the dissociation constant between two proteins is expressed as:

$$K_D = \frac{C^a \times C^b}{C^{ab}}$$

Where $C^a$ represents the concentration of protein A, $C^b$ represents the concentration of protein B, and $C^{ab}$ represents the concentration of the complex (Sudhaharan et al., 2009). Since we counted the average number of molecules inside 2.5 μm long cells, and determined the volume of a 2.5 μm long and 800nm wide *E.coli* cell to be ~ 1.1 $\mu m^3$ (Kubitschek and Friske, 1986)(Kubitschek and Friske, 1986) we assume 1 molecule/cell corresponds to a concentration of 1nM. Therefore, the concentrations in living cells, of ParC, ParE, and MukB are 289nM, 210nM, and 195nM respectively. To calculate the dissociation constant of TopoIV heterotetramers we assumed that a dimer of ParC will form a heterotetramer with a dimer of ParE.

**15. Determination of Search Time Preceding Catalysis.**

Since we measured the fraction of catalytically active TopoIV at any given time, $F_{catalysis}$, to be 14% (Figure 3B), and we measured the average dwell of the catalytic cycle, $t_{catalysis}$, to be 1.8 s (Figure 3D) we can calculate the average time for a given TopoIV molecule to locate and bind to its substrate, $t_{search}$, using $F_{catalysis} = t_{catalysis}/(t_{catalysis} + t_{search})$ (Uphoff et al., 2013). Using our measured values of $F_{catalysis}$ and $t_{catalysis}$ to solve this equation gives a search time of 11s. The combined catalytic dwell time and search time can be used to determine the reaction rate per molecule: $1/(t_{catalysis} + t_{search})$. Using this we found that a single TopoIV has a reaction rates of ~ 4.7 $min^{-1}$. This high rate of nearly five reactions per minute per molecule explains our observations that the fraction of catalytic events captured by norfloxacin treatment reaches saturation in less than a few minutes after addition of the antibiotic.

**16. Determining ParC/E *D** Distributions at Different Points in the Cell Cycle**

Cells were segmented from brightfield images using MicrobeTracker, giving a cell outline and a cell midline (Sliusarenko et al., 2011) and the positions of molecule

trajectories were determined relative to the cell midline, with the x-axis defined as the cell short axis and the y-axis defined as the cell long axis. Cells were binned by cell length (which is a reasonable proxy for their stage in the cell cycle), into three categories; short cells (2-3 μm long) having a single centrally located nucleoid, intermediate length cells (3-4 μm long), and longer cells (4-5 μm long) having replicated most of their chromosome which separate into distinct nucleoids prior to cell division (Figure S5F). Localizations within segmented cell boundaries were tracked and $D^*$ values determined. Fitting the distribution of $D^*$ values for ParC/E in small, intermediate, and long cells showed a similar proportion of heterotetramers in cells at these three different stages of the cell cycle (Figure S5G and H).

## References


Bachmann, B.J. (1972). Pedigrees of some mutant strains of Escherichia coli K-12. Bacteriological reviews *36*, 525-557.

Badrinarayanan, A., Reyes-Lamothe, R., Uphoff, S., Leake, M.C., and Sherratt, D.J. (2012). In vivo architecture and action of bacterial structural maintenance of chromosome proteins. Science *338*, 528-531.

Crisona, N.J., Strick, T.R., Bensimon, D., Croquette, V., and Cozzarelli, N.R. (2000). Preferential relaxation of positively supercoiled DNA by E. coli topoisomerase IV in single-molecule and ensemble measurements. Genes & development *14*, 2881-2892.

Crocker, J.C., and Grier, D.G. (1996). When Like Charges Attract: The Effects of Geometrical Confinement on Long-Range Colloidal Interactions. Physical review letters *77*, 1897-1900.

Datsenko, K.A., and Wanner, B.L. (2000). One-step inactivation of chromosomal genes in Escherichia coli K-12 using PCR products. Proceedings of the National Academy of Sciences of the United States of America *97*, 6640-6645.

Durisic, N., Laparra-Cuervo, L., Sandoval-Alvarez, A., Borbely, J.S., and Lakadamyali, M. (2014). Single-molecule evaluation of fluorescent protein photoactivation efficiency using an in vivo nanotemplate. Nature methods *11*, 156-162.

Elmore, S., Muller, M., Vischer, N., Odijk, T., and Woldringh, C.L. (2005). Single-particle tracking of oriC-GFP fluorescent spots during chromosome segregation in Escherichia coli. Journal of structural biology *151*, 275-287.

Guzman, L.M., Belin, D., Carson, M.J., and Beckwith, J. (1995). Tight regulation, modulation, and high-level expression by vectors containing the arabinose PBAD promoter. Journal of bacteriology *177*, 4121-4130.

Kubitschek, H.E., and Friske, J.A. (1986). Determination of bacterial cell volume with the Coulter Counter. Journal of bacteriology *168*, 1466-1467.

Landgraf, D., Okumus, B., Chien, P., Baker, T.A., and Paulsson, J. (2012). Segregation of molecules at cell division reveals native protein localization. Nature methods *9*, 480-482.

Lee, I., Dong, K.C., and Berger, J.M. (2013). The role of DNA bending in type IIA topoisomerase function. Nucleic acids research *41*, 5444-5456.

Lesterlin, C., Ball, G., Schermelleh, L., and Sherratt, D.J. (2014). RecA bundles mediate homology pairing between distant sisters during DNA break repair. Nature *506*, 249-253.

Moolman, M.C., Krishnan, S.T., Kerssemakers, J.W., van den Berg, A., Tulinski, P., Depken, M., Reyes-Lamothe, R., Sherratt, D.J., and Dekker, N.H. (2014). Slow unloading leads to DNA-bound beta2-sliding clamp accumulation in live Escherichia coli cells. Nature communications *5*, 5820.

Reyes-Lamothe, R., Sherratt, D.J., and Leake, M.C. (2010). Stoichiometry and architecture of active DNA replication machinery in Escherichia coli. Science *328*, 498-501.

Sanamrad, A., Persson, F., Lundius, E.G., Fange, D., Gynna, A.H., and Elf, J. (2014). Single-particle tracking reveals that free ribosomal subunits are not excluded from the Escherichia coli nucleoid. Proceedings of the National Academy of Sciences of the United States of America *111*, 11413-11418.

Sliusarenko, O., Heinritz, J., Emonet, T., and Jacobs-Wagner, C. (2011). High-throughput, subpixel precision analysis of bacterial morphogenesis and intracellular spatio-temporal dynamics. Molecular microbiology *80*, 612-627.

Stone, M.D., Bryant, Z., Crisona, N.J., Smith, S.B., Vologodskii, A., Bustamante, C., and Cozzarelli, N.R. (2003). Chirality sensing by Escherichia coli topoisomerase IV and the mechanism of type II topoisomerases. Proceedings of the National Academy of Sciences of the United States of America *100*, 8654-8659.

Stracy, M., Lesterlin, C., Garza de Leon, F., Uphoff, S., Zawadzki, P., and Kapanidis, A.N. (2015). Live-cell superresolution microscopy reveals the organization of RNA polymerase in the bacterial nucleoid. Proceedings of the National Academy of Sciences of the United States of America.

Stracy, M., Uphoff, S., Garza de Leon, F., and Kapanidis, A.N. (2014). In vivo single-molecule imaging of bacterial DNA replication, transcription, and repair. FEBS letters *588*, 3585-3594.

Sudhaharan, T., Liu, P., Foo, Y.H., Bu, W., Lim, K.B., Wohland, T., and Ahmed, S. (2009). Determination of in vivo dissociation constant, KD, of Cdc42-effector complexes in live mammalian cells using single wavelength fluorescence cross-correlation spectroscopy. The Journal of biological chemistry *284*, 13602-13609.

Thomason, L.C., Costantino, N., and Court, D.L. (2007). E. coli genome manipulation by P1 transduction. Current protocols in molecular biology / edited by Frederick M. Ausubel ... [et al.] *Chapter 1*, Unit 1 17.

Tokunaga, M., Imamoto, N., and Sakata-Sogawa, K. (2008). Highly inclined thin illumination enables clear single-molecule imaging in cells. Nature methods *5*, 159-161.

Uphoff, S., Reyes-Lamothe, R., Garza de Leon, F., Sherratt, D.J., and Kapanidis, A.N. (2013). Single-molecule DNA repair in live bacteria. Proceedings of the National Academy of Sciences of the United States of America *110*, 8063-8068.

Uphoff, S., Sherratt, D.J., and Kapanidis, A.N. (2014). Visualizing protein-DNA interactions in live bacterial cells using photoactivated single-molecule tracking. Journal of visualized experiments : JoVE.

Wang, S., Moffitt, J.R., Dempsey, G.T., Xie, X.S., and Zhuang, X. (2014). Characterization and development of photoactivatable fluorescent proteins for single-molecule-based superresolution imaging. Proceedings of the National Academy of Sciences of the United States of America *111*, 8452-8457.

Wang, X., Reyes-Lamothe, R., and Sherratt, D.J. (2008). Modulation of Escherichia coli sister chromosome cohesion by topoisomerase IV. Genes & development *22*, 2426-2433.

## Supplementary Figures

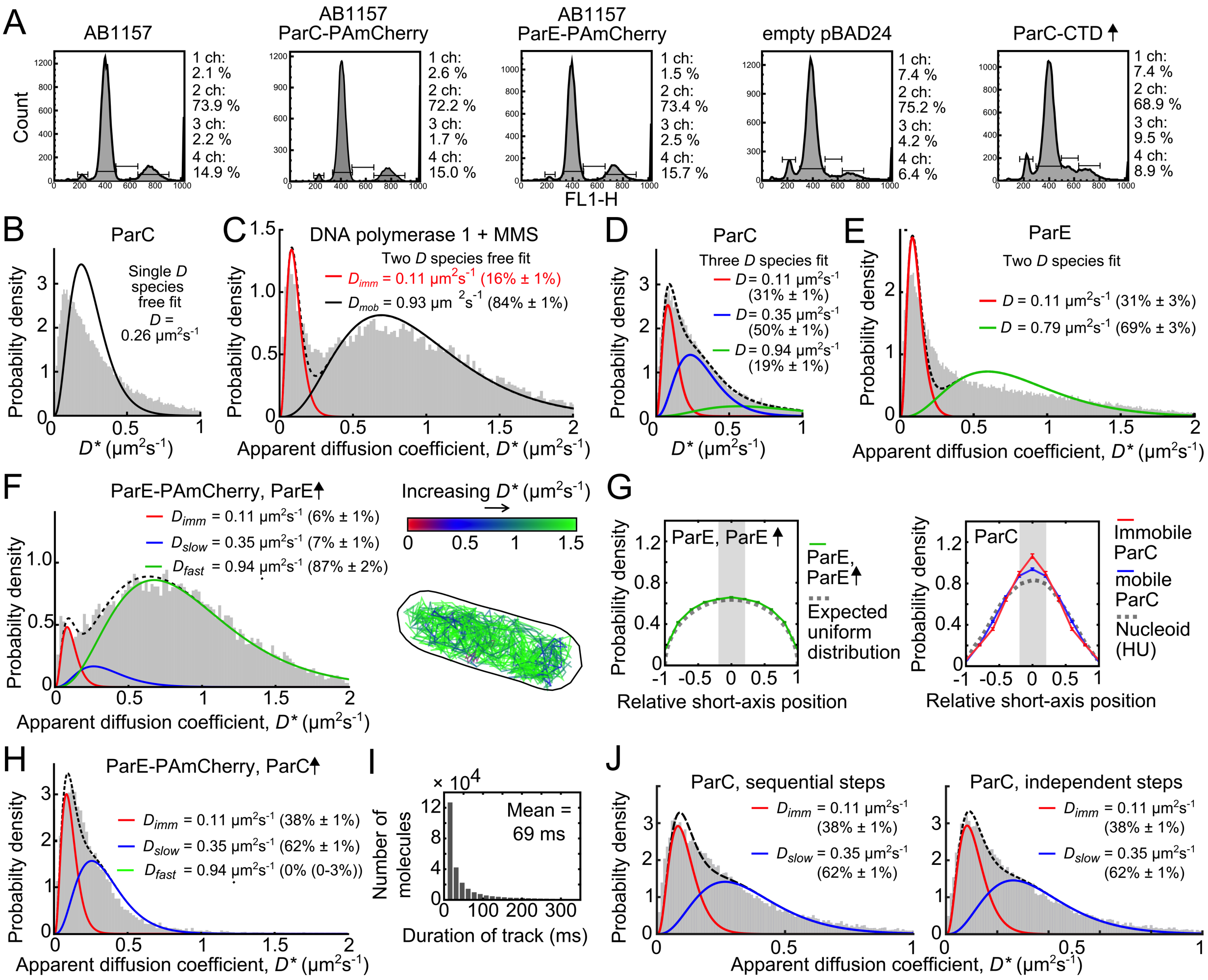


**Figure S1. PALM Tracking of ParC/E Molecules in Live *E. coli.*** Related to Figure 1. A) Flow cytometry profiles of cells with PAmCherry fusions (derivatives of AB1157) and after ParC-CTD over-expression in strain KK52. Ranges indicate cells with (from the left); one, two, three and four chromosomes. B) The distribution of apparent diffusion coefficients (*D**) of 73020 ParC molecules, fitted with a single species model. C) The distribution of apparent diffusion coefficients, *D**, for DNA polymerase1- PAmCherry grown in minimal media supplemented with MMS (100 mM). The distribution was fitted with a two species free fit, giving a population of specifically bound molecules with $D_{imm}$ = 0.11 μm$^2$s$^{-1}$. D) The distribution of apparent diffusion coefficients (*D**) of 73020 ParC molecules, fitted with a three species model. Immobile population was constrained at value obtained for immobile molecules. We could not find any biological justification to fit three species model to ParC distribution. The resulting population of $D_{slow}$ = 0.30 μm$^2$s$^{-1}$ and $D_{fast}$ = 0.65 μm$^2$s$^{-1}$ could not be seen in any of the control overexpression experiments. Therefore, a three species fit does not describe true populations of ParC.  E) The distribution of

apparent diffusion coefficients (D*) of 64551 ParE molecules fitted with two species model. Immobile population was constrained. The ParE data do not fit well to two species model well. F) The distribution of *D** values of 7101 ParE-PAmCherry molecules in a strain overexpressing unlabeled ParE (left). The distribution was fitted to a three species model with two species constrained at $D_{imm}$ = 0.11 μm$^2$s$^{-1}$ and $D_{slow}$ = 0.35 μm$^2$s$^{-1}$. The third unconstrained fit shows the majority of molecules are fast moving, with $D_{fast}$ = 0.94 μm$^2$s$^{-1}$. An example cell with ParE trajectories colored according to their *D** value (right). G) The probability density of ParE molecules across the cell short-axis in a strain overexpressing unlabeled ParE (left; 377 cells). 25% of molecules were located in the central region of the short axis (defined as the central 20% shown highlighted in grey). Dashed grey line shows the expected distribution for molecules uniformly distributed throughout a cylindrical cell volume. 25% of uniformly distributed molecules were expected to be located in the central region, agreeing well with the data. The short-axis distribution of immobile and mobile ParC molecules from Figure 3A is reproduced here for comparison (right). 39 % of immobile ParC molecules and 34% of mobile ParC molecules were located in the central region of the short-axis. The distribution of nucleoid associated protein HU (dashed line) shows the average spatial distribution of the nucleoid. H) The distribution of *D** values of 9508 ParE-PAmCherry molecules in a strain overexpressing unlabeled ParC. The distribution was fitted to a three species model with D values constrained at $D_{imm}$ = 0.11 μm$^2$s$^{-1}$, $D_{slow}$ = 0.35 μm$^2$s$^{-1}$ and $D_{fast}$ = 0.94 μm$^2$s$^{-1}$. After ParC overexpression no fast moving molecules were detected. I) Track length distribution for ParC molecules imaged at 15 ms exposure time. J) The *D** distribution from sequential steps was essentially identical to the distribution generated with non-overlapping steps and fitting both with Equation 4 gave identical values. Fitting ranges give 95% confidence intervals.

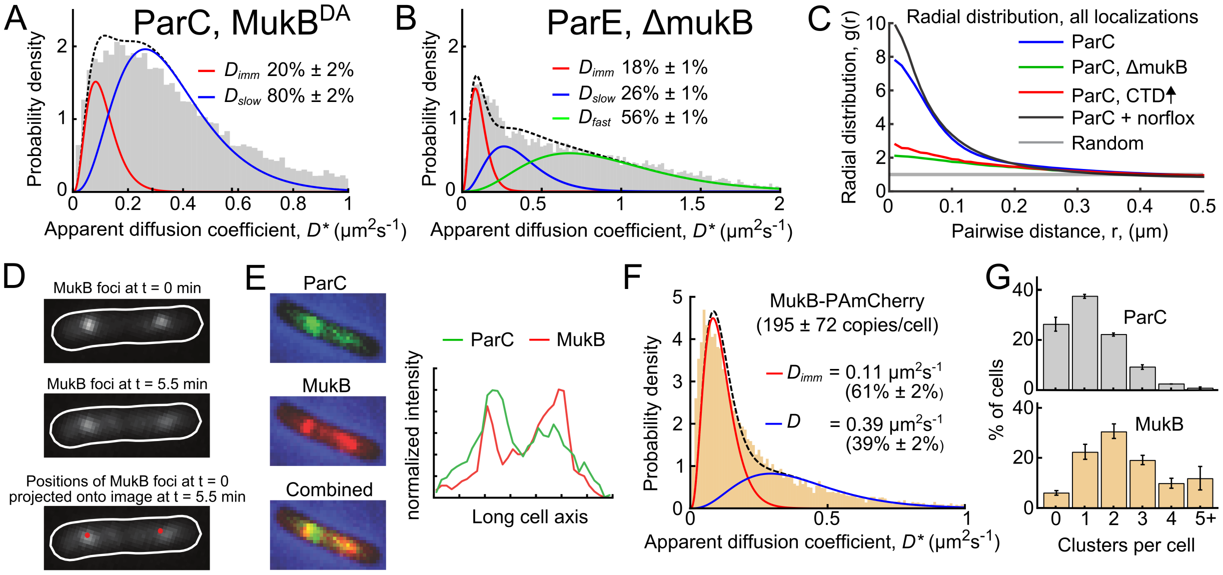


**Figure S2. MukB Influences the Diffusion and Organization of TopoIV.** Related to Figure 2. A) The distribution of $D^*$ values for 13188 ParC molecules in *mukB*$^{DA}$ cells. Distributions of D* were fitted with two species model with both D values constrained. B) The distribution of $D^*$ values for 15893 ParE molecules in Δ*mukB* cells. Distributions of D* were fitted with three species model with all D values constrained. C) Radial distribution function calculated for all ParC localizations in WT conditions, with ParC-CTD overexpression, MukB deletion, and norfloxacin incubation. This analysis provides an unbiased assessment of cluster formation. D) Location of MukB foci before and after ~5 min PALM acquisition in example cell. E) Example of a cell imaged in epifluorescence with labeled MukB-mCherry foci (red) and ParC-YPet foci (green). The long-axis lines-scan of the fluorescence intensity is shown on the right. Frequently we observed only some of MukB foci were associated with distinct ParC clusters. F) Distribution of $D^*$ values for 9068 MukB-PAmCherry trajectories. The distribution was fitted to a two species model with $D_{imm}$ constrained at 0.11 μm$^2$s$^{-1}$ and a second unconstrained fit. G) The number of clusters per cell formed by MukB defined by the same clustering parameters used for ParC. For comparison the number of ParC clusters per cell (Fig. 2A) is also shown here. MukB forms ~ twice as many clusters as ParC. Error bars indicate standard deviation of three experimental repeats. Fitting ranges give 95% confidence intervals.

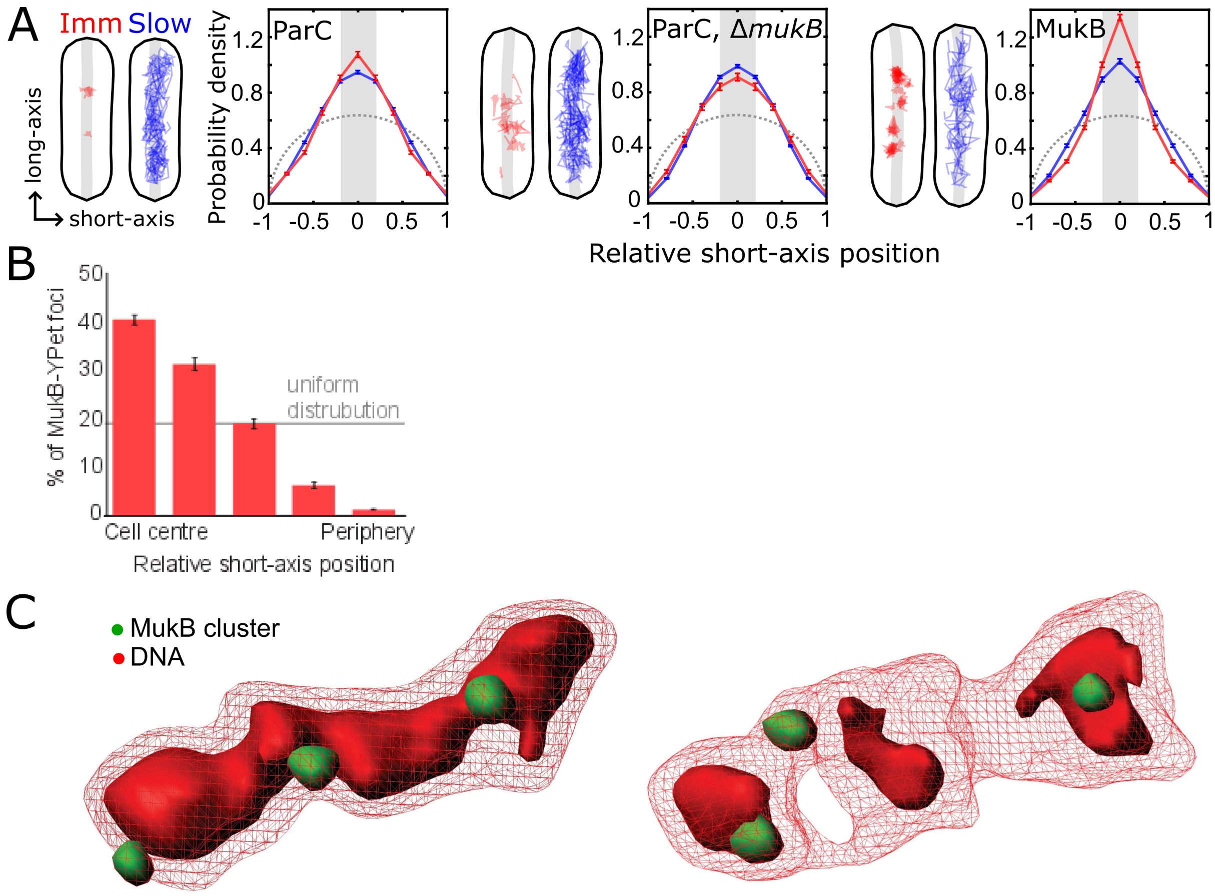


**Figure S3. Intracellular Organization of MukB and TopoIV.** Related to Figure 2. A) Example cells show intracellular location of ParC molecules sorted by their D* value as immobile (red) and slow (blue). Probability distribution of immobile and slow moving ParC molecules across the short cell axis in wild type (2386 cells) and DmukB (253 cells). Also shown are immobile and slow MukB molecules in wild-type cells (852 cells). Cell width is normalized to 0 at the cell midline and 1 and -1 at the cell membrane. Dotted lines indicate random distribution in a cellular cylinder and grey bars indicate central 20% of cell. Error bars show the square root of the number of observations per histogram bin assuming Poissonian statistics. B) Distributions of MukBEF foci across the short cell axis, in epifluorescence microscopy (1407 cells). Segments of the lateral positions were normalized to cell volume. For a uniform distribution, an equal fraction of foci in each segment is expected. Error bars indicate standard deviation of three experimental repeats. C) 3D-SIM surface rendering of two cells with MukB-mYPet and DAPI stained DNA. Green spots; MukBEF foci. Wireframe shows nucleoid DNA (threshold: excludes signal ≤20% of the maximum

DAPI intensity); red surface shows the DNA regions with the highest density (threshold: ≥ 70% of the maximum DAPI intensity). See Movie 1.

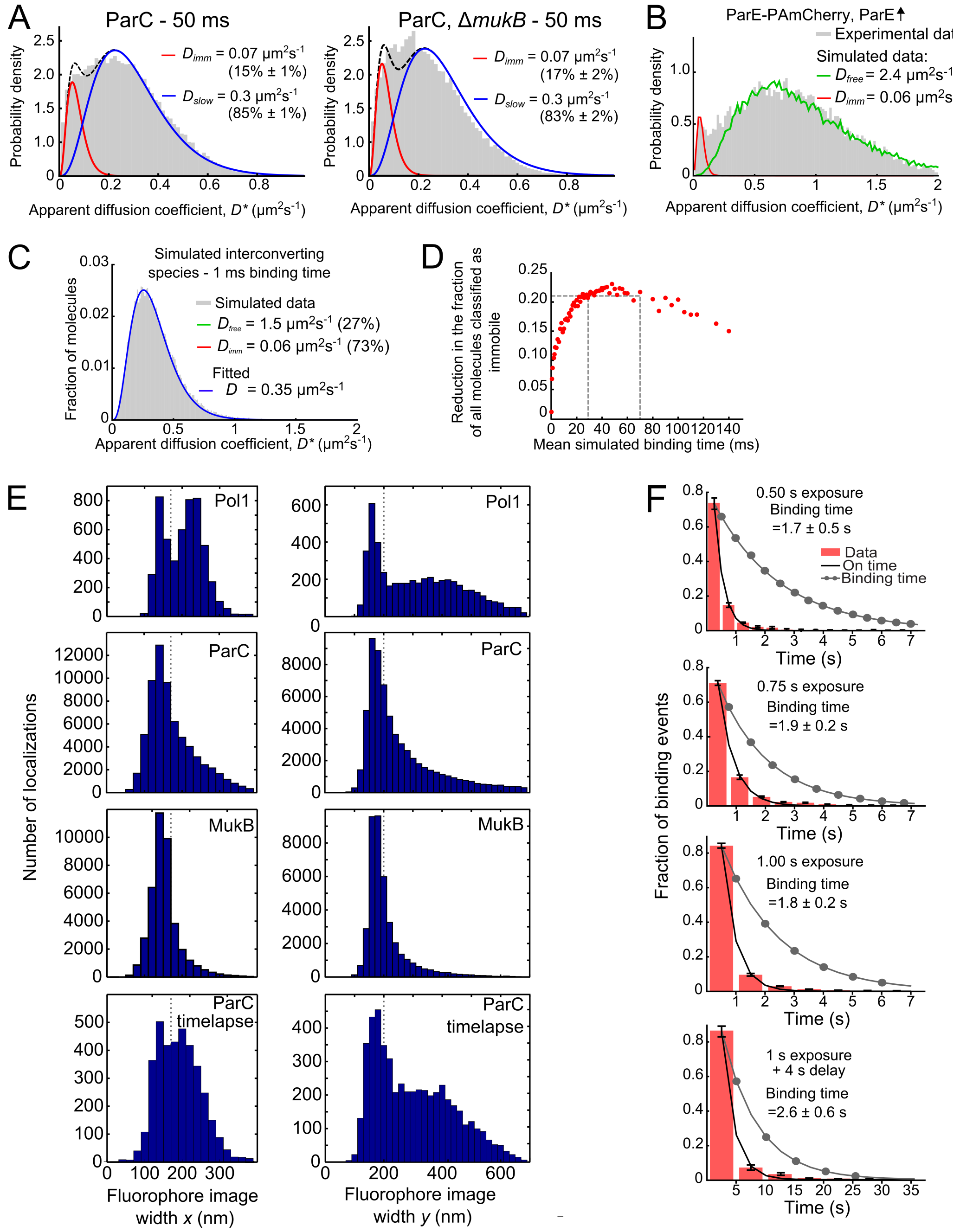


**Figure S4. Two Populations of Immobile ParC.** Related to Figure 3.

A) Distribution of D* values for 28188 ParC molecules imaged with a time-lapse using a 15 ms exposure followed by a 35 ms delay (left). Distribution of D* values for 26636 ParC molecules in ΔmukB cells imaged with a time-lapse (right). B) Comparison between experimental data for ParE-PAmCherry molecules in a strain

overexpressing unlabeled ParE and the distributions of two simulated diffusing species. The fast moving population agrees well with simulated molecules with Dfree = 2.4 µm2s-1. The immobile population agrees well with simulated molecules with Dimm = 0.06 µm2s-1. C) Simulated molecules interconverting between Dimm and Dfree = 1.5 µm2s-1 (corrected for the relative size of TopoIV compared to ParE). The binding time in the immobile state is set to 1 ms and the ratio between states is set to Dimm (73%) and Dfree (27%). The distribution of D* values from the simulated interconverting species with transient binding fits well to a single species at D = 0.35 µm2s-1, matching our observed Dslow population. D) Transition categorization analysis of simulated interconverting species with varying binding dwell times. Tracks were simulated with 15 ms exposure times and in time-lapse with 15 ms exposures followed by a 35 ms delay, then categorized in the same way as experimental data in Fig. 4B, and the reduction in the total fraction of molecules categorized as immobile between normal and time-lapse simulations was plotted against the binding time. A range of binding times between 30 and 70 ms agree with the observed reduction in immobile ParC in time-lapse experiments (reduced from 35% to 14%). E) Short axis (X axis) and long axis (Y axis) elliptical Gaussian fit widths at 750 ms exposure time for Pol1 + MMS, ParC, MukB and ParC in time-lapse experiment where 1 s exposures were followed by 4 s delays. We used thresholds of < 160 nm short axis-width and < 200 nm long axis-width to identify immobile molecules determined from the Pol1 control where immobile and mobile molecules are clearly resolvable (dashed line) (Uphoff et al, 2013; Fig.S1C). F) On-time distributions for bound ParC at 0.5 s exposure, 0.75 s exposure, 1 s exposure times and 1 s = 4 s time-lapse conditions. Exponential fits (solid lines) and photobleaching-corrected binding time distributions (dashed circled lines). Error bars shows standard deviation of three experimental repeats. Fitting ranges give 95% confidence intervals.

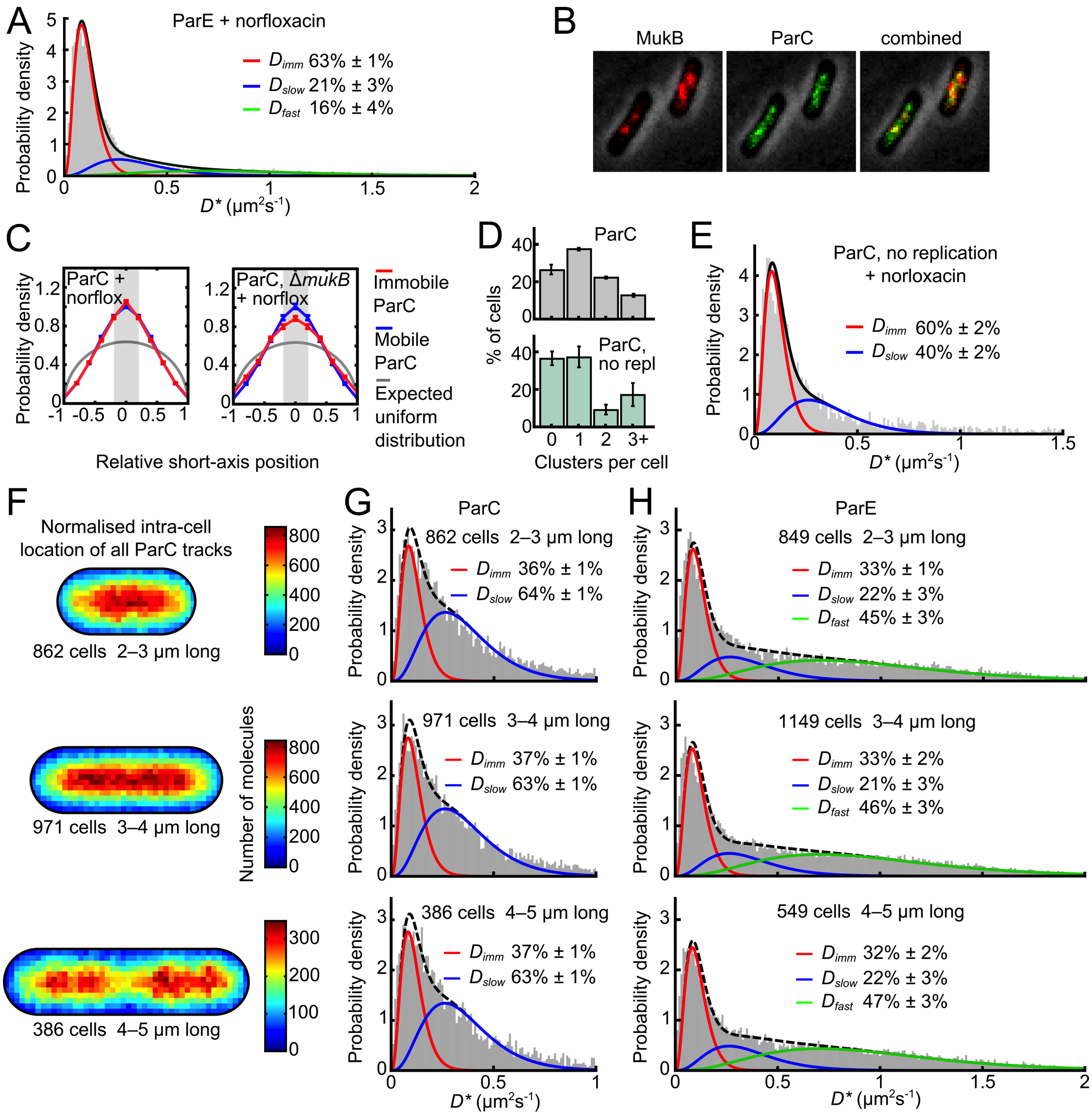


**Figure S5. Catalytically Active TopoIV.** Related to Figure 4.

A) Distribution of $D^*$ values for 17001 ParE molecules after 10min treatment with norfloxacin. ParE and ParC had a similar fraction of immobile molecules after norfloxacin treatment. B) Example cells treated with norfloxacin. Epifluorescence microscopy shows MukB-mcherry foci are still present and some of ParC-YPet clusters are associated with these MukB foci. C) The probability density of ParC molecules across the cell short-axis in 858 cells treated with norfloxacin (left). Dashed grey line shows the expected distribution for molecules uniformly distributed throughout a cylindrical cell volume. The mobile and immobile populations have similar distributions, both with 36 % of molecules located in the central 20 % of the width (grey shaded area). The distribution of ParC molecules across the cell short-axis in 463 Δ*mukB* cells treated with norfloxacin (right). The mobile population has 37 % of molecules located in the central 20 % of short-axis, whereas the immobile

population shows fewer molecules (33 %) in the central region. D) The number of ParC clusters per cell in cells not undergoing replication (right). We observed a small decrease in number of clusters in cells not undergoing replication, compared to the entire population of cells (right), indicating that ParC foci are not dependent on the presence of replication. Instead, persistence of ParC foci supports our conclusion that their formation is dependent on MukB. Only small cells without replisomes were used in this analysis, therefore a small decrease in the number of ParC foci was expected due to the smaller average size compared to the entire population of cells. E) Distribution of *D** values for 2479 ParC molecules in cells not undergoing replication after 10min treatment with norfloxacin. Distributions of D* were fitted with two species model with both D values constrained. F) Normalized 2D histograms showing the average spatial distribution of ParC localizations from many cells. Binning by cell length from short to long cells shows progression through the cell cycle, with short cells (2-3 μm long) having a single centrally located nucleoid, and longer cells (4-5 μm long) having two clearly separate nucleoids. G) Distribution of ParC *D** values from 862 short cells (top), 971 medium-length cells (middle), and 386 long cells (bottom). Distributions of D* were fitted with two species model with both D values constrained. H) Distribution of ParE *D** values from 849 short cells (top), 1149 medium-length cells (middle), and 549 long cells (bottom). Distributions of D* were fitted with three species model with all D values constrained. Fitting ranges give 95% confidence intervals

**Table S1.** Bacterial Strains. Related to the Experimental Procedures; section 1. Bacterial Strains and Cell Preparation

| Strain | Genotype[a] |
|---|---|
| AB1157 | $F^-$, $\lambda^-$, $rac^-$, *thi-1, hisG4, Δ(gpt-proA)62, argE3, thr-1, leuB6, kdgK51, rfbD1, araC14, lacY1, galK2, xylA5, mtl-1, tsx-33, supE44(glnV44), rpsL31(strR), qsr'-0, mgl-51* (Bachmann, 1972) |
| PZ103 | *parE:: PAmCherry kan* |
| KK10 | *parC::PAmCherry kan* |
| PZ147 | *parC::PAmCherry kan; mukB::mYPet frt;* $gyrA^{L83}$ *tet* |

| | |
|---|---|
| PZ120 | *parE:: PAmCherry kan; mukB:: mYPet frt;* $gyrA^{L83}$ *tet* |
| PZ124 | *parE:: PAmCherry kan; dnaN:: mYPet frt;* $gyrA^{L83}$ *tet* |
| PZ125 | *parC:: PAmCherry kan; dnaN:: mYPet frt;* $gyrA^{L83}$ *tet* |
| PK13 | *parC:: PAmCherry kan; tetR:: mYPet frt; tetO at ori1 gen* |
| PK16 | *parC:: PAmCherry kan; tetR:: mYPet frt; tetO at ter3 gen* |
| PZ109 | *parE:: PAmCherry kan; tetR:: mYPet frt; tetO at ori1 gen* |
| PZ114 | *parE:: PAmCherry kan; tetR:: mYPet frt; tetO at ter3 gen* |
| PZ128 | *parE:: PAmCherry frt; ΔmukB kan; dnaN:: mYPet frt;* $gyrA^{L83}$ *tet* |
| PZ129 | *parC:: PAmCherry frt; ΔmukB kan; dnaN:: mYPet frt;* $gyrA^{L83}$ *tet* |
| Ab238 | *MukB:: PAmCherry kan (Badrinarayanan et al., 2012)* |
| PZ122 | *parC::yPet kan; mukB::mcherry frt;* $GyrA^{L83}$ *tc* |
| KG52 | *lacO240::hyg* at *ori1; tetO240::gen* at *ter3; plac-lacI-mCherry* at *leuB, plac-lacI-tetR-mCerulean* at *galK, mYPet-DnaN kan* |
| AB1157 MukB-mYPet | MukB::mYPet *kan* |

[a] All strains were in the AB1157 background and were constructed in this study unless otherwise stated.
[b] Abbreviations: *kan*, kanamycin resistance gene; *tet*, tetracycline resistance gene; *gen*, gentamicin resistance gene; hyg, hygromycin resistance gene; *frt*, FLP site-specific recombination site.

**Table S2.** Oligonucleotides and Plasmids. Related to the Experimental Procedures; section 1. Bacterial Strains and Cell Preparation

| Name | Description | Reference |
|---|---|---|
| ParCpam cherryfor | GTGTTGAGATCGACTCTCCTCGCCGTGCCAGCA GCGGTGATAGCGAAGAG**TC**GGCTGGCTCCGCTG | This study |
| ParCpam cherryrev | TTTCATCCGGCGTTCCTTGCAAGCGGGAGGAAAC AGCGCCCTCCCCGGCATATATGAATATCCTCCTT AGTTC | This study |
| ParEpam cherryfor | ATCGCCGCAACTGGTTGCAAGAGAAAGGCGACA TGGCGGAGATTGAGGTT TCG GCT GGC TCC GCT GCT GGT TC | This study |
| ParEpam | TAATCCTGCCTTGTTTGCCCGGCCATCCTGACCG | This study |

| | | |
|---|---|---|
| cherryrev | GGCAATGTTCTTTCCT GAGGATCCCATATGAATATCCTCC | |
| pZ68 | ParC-CTD (amino acid residues 497-806) cloned into pBAD24 NcoI and XbaI cloning sites | This study |
| pZ65 | Full length ParE cloned into pBAD24 using NcoI and XbaI cloning sites | This study |

**Table S3.** Behavior of Fluorescent Fusions and strain overexpressing ParC-CTD (KG52). Related to the Experimental Procedures; section 1. Bacterial Strains and Cell Preparation

| | AB1157 | ParC-PAmCherry (KK10) | ParE-PAmCherry (PZ103) | KG52 pBAD24 | ParC-CTD Overexpression pZ68 |
|---|---|---|---|---|---|
| Generation time | 86 ± 3 min | 85 ± 5 min | 81 ± 11 min | 95 ± 1.3 min | 101 ± 3.9 min |
| Cell length | 3.1 ± 0.5 μm | 3.3 ± 0.7 μm | 3.1 ± 0.6 μm | 3.7 ± 0.8 μm | 3.7 ± 0.8 μm |
| Anucleate cells | not determined | 0/86 | 1/77 | 0/80 | 1/68 |

**Table S4.** Exponential time constants for PAmCherry photobleaching $t_{bleach}$, measured ParC on-times $t_{on}$, and corrected ParC binding times $t_{bound}$. (±SD). Related to Figure 3 and Figure S4.

| | 500 ms | 750 ms | 1000 ms |
|---|---|---|---|
| $t_{bleach\ [S]}$ | 0.423 | 0.647 | 0.672 |
| $t_{on\ [S]}$ | 0.328 ± 0.02 | 0.503 ± 0.03 | 0.484 ± 0.02 |
| $t_{bound\ [S]}$ | 1.68 ± 0.52 | 1.94 ± 0.22 | 1.83 ± 0.16 |

**Movie 1. Visualization of 3D structured illumination images showing the organization of MukB foci inside living cells.** Related to Figure 2 and Figure S3. MukB--mYPet fluorescence is shown in green, and DAPI stained DNA is shown in red.
