# Supplementary figures and images for: The Localization and Action of Topoisomerase IV in Escherichia coli Chromosome Segregation Is Coordinated by the SMC Complex, MukBEF
Source: Cell Rep. 2015 Dec 10;13(11):2587–96. doi: 10.1016/j.celrep.2015.11.034 (PMC5061553; doi:10.1016/j.celrep.2015.11.034)

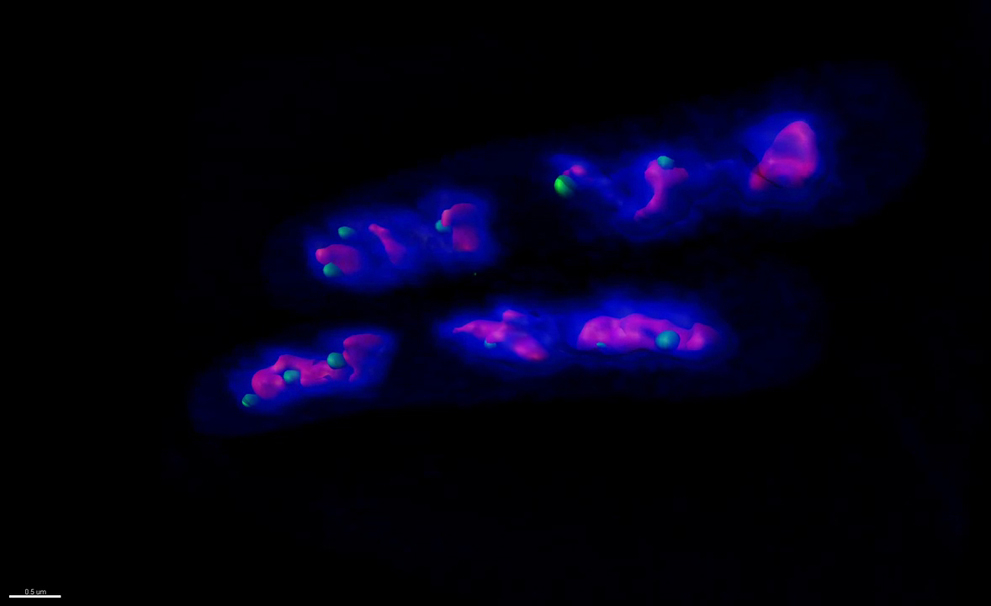

Supplement: Movie S1. Visualization of 3D Structured Illumination Images Showing the Organization of MukB Foci Inside Living Cells, Related to Figures 2 and S3 — MukB-mYPet fluorescence is shown in green, and DAPI-stained DNA is shown in red. [file mmc2.jpg]
