# Supplementary material for: The Localization and Action of Topoisomerase IV in Escherichia coli Chromosome Segregation Is Coordinated by the SMC Complex, MukBEF
Source: Cell Rep. 2015 Dec 10;13(11):2587–96. doi: 10.1016/j.celrep.2015.11.034 (PMC5061553; doi:10.1016/j.celrep.2015.11.034)
Supplement: Document S2. Article plus Supplemental Information [file mmc3.pdf]

Cell Reports

Article

The Localization and Action of Topoisomerase IV in *Escherichia coli* Chromosome Segregation Is Coordinated by the SMC Complex, MukBEF

Graphical Abstract

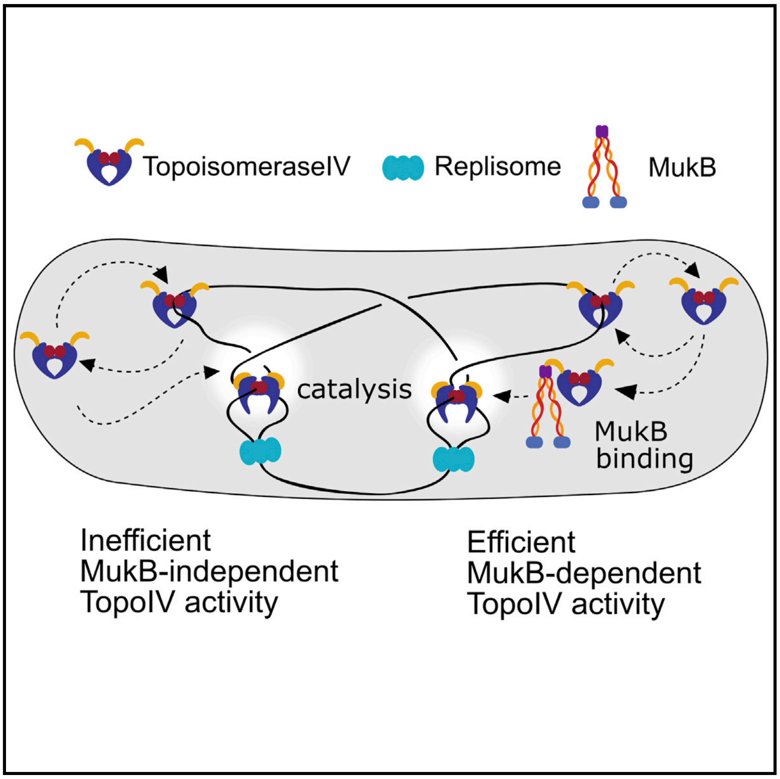

Highlights

- Individual molecules of topoisomerase IV (TopoIV) were tracked in live *E. coli* cells
- TopoIV was monitored in cellular space and in time throughout the cell cycle
- The interaction of TopoIV and MukBEF directs TopoIV to its sites of action
- The TopoIV-MukBEF interaction promotes timely segregation of newly replicated DNA

Authors

Pawel Zawadzki, Mathew Stracy, Katarzyna Ginda, Katarzyna Zawadzka, Christian Lesterlin, Achillefs N. Kapanidis, David J. Sherratt

Correspondence

david.sherratt@bioch.ox.ac.uk

In Brief

Zawadzki et al. use quantitative single-molecule imaging to examine the behavior of topoisomerase IV molecules in their native environment inside *Escherichia coli* cells. The work reveals how the functional interaction between topoisomerase IV and the SMC complex, MukBEF, aids chromosome segregation.

CrossMark

Zawadzki et al., 2015, Cell Reports *13*, 2587–2596
December 22, 2015 © 2016 The Authors.
http://dx.doi.org/10.1016/j.celrep.2015.11.034

CellPress

Cell Reports
Article

OPEN ACCESS
CellPress

The Localization and Action of Topoisomerase IV in *Escherichia coli* Chromosome Segregation Is Coordinated by the SMC Complex, MukBEF

Pawel Zawadzki,[1,4] Mathew Stracy,[2,4] Katarzyna Ginda,[1] Katarzyna Zawadzka,[1] Christian Lesterlin,[1,3] Achillefs N. Kapanidis,[2] and David J. Sherratt[1,*]
[1]Department of Biochemistry, University of Oxford, South Parks Road, Oxford OX1 3QU, UK
[2]Biological Physics Research Group, Clarendon Laboratory, Department of Physics, University of Oxford, Parks Road, Oxford OX1 3PU, UK
[3]Bases Moléculaires et Structurales des Systèmes Infectieux, UMR 5086, Centre National de la Recherche Scientifique, University of Lyon, 69367 Lyon, France
[4]Co-first author
*Correspondence: david.sherratt@bioch.ox.ac.uk
http://dx.doi.org/10.1016/j.celrep.2015.11.034
This is an open access article under the CC BY license (http://creativecommons.org/licenses/by/4.0/).

SUMMARY

The type II topoisomerase TopoIV, which has an essential role in *Escherichia coli* chromosome decatenation, interacts with MukBEF, an SMC (structural maintenance of chromosomes) complex that acts in chromosome segregation. We have characterized the intracellular dynamics of individual TopoIV molecules and the consequences of their interaction with MukBEF clusters by using photoactivated-localization microscopy. We show that ∼15 TopoIV molecules per cell are associated with MukBEF clusters that are preferentially localized to the replication origin region (*ori*), close to the long axis of the cell. A replication-dependent increase in the fraction of immobile molecules, together with a proposed catalytic cycle of ∼1.8 s, is consistent with the majority of active TopoIV molecules catalyzing decatenation, with a minority maintaining steady-state DNA supercoiling. Finally, we show that the MukB-ParC interaction is crucial for timely decatenation and segregation of newly replicated *ori* DNA.

INTRODUCTION

Segregation of newly replicated chromosomes must be completed before cell division can occur. Two classes of proteins play important roles in DNA segregation: topoisomerases and structural maintenance of chromosome (SMC) complexes.

DNA replication introduces positive (+) supercoiling ahead of the replication fork, and rotation of the forks leads to interlinking of the two sister chromosomes, generating (pre)catenanes behind the replisomes throughout the chromosome. In *Escherichia coli*, ∼225,000 catalytic events by the type II topoisomerases DNA gyrase and topoisomerase IV (TopoIV) are required for each generation to unlink the 4.6-Mb chromosome. DNA gyrase acts preferentially ahead of the replication fork to remove (+) supercoiling (Koster et al., 2010; Vos et al., 2011). TopoIV removes the majority of links behind the replication forks (Joshi et al., 2013; Wang et al., 2008), whereas the type I topoisomerase TopoIII is able to remove links in single-stranded DNA regions (Koster et al., 2010; Vos et al., 2011) and FtsK-dependent XerCD recombination at *dif* is able to remove catenation links in *ter* (Grainge et al., 2007).

Heterotetrameric TopoIV consists of dimers of ParC (the DNA binding and catalytic subunit) and ParE (the regulatory ATPase). It changes DNA topology by introducing a double-stranded break in DNA and passing a second duplex segment of DNA through the break before resealing it. TopoIV acts on topologically different substrates including (+) and negative (−) supercoiled DNA and knotted and catenated DNA (Koster et al., 2010; Postow et al., 2001; Vos et al., 2011). Its essential cellular role is in decatenation of newly replicated DNA (Joshi et al., 2013; Wang et al., 2008). The mechanism of how TopoIV recognizes and discriminates its substrates and which substrate is preferred in vivo is not fully understood (Lee et al., 2013; Vos et al., 2013a).

A second class of proteins, SMC complexes, play an equally important role in faithful DNA segregation (Hirano, 2006). Despite sharing little primary amino acid sequence homology with other SMC complexes, the *E. coli* complex MukBEF retains much of the distinctive SMC architecture (Nolivos and Sherratt, 2014; Woo et al., 2009), forming dimers joined at a hinge domain located at one end of an ∼50-nm-long intramolecular coiled coil with an ATPase head domain at the other end of the coiled coil. Inactivation of the MukB protein or either of the two accessory proteins, MukE and MukF, results in abnormal chromosome organization and segregation (Danilova et al., 2007; Nolivos and Sherratt, 2014). The MukB dimerization hinge has been shown to physically interact in vitro with ParC, which stimulates TopoIV-mediated relaxation of (−) supercoils (Hayama and Marians, 2010; Li et al., 2010). An enrichment of ParC/E molecules in the vicinity of *ori*-associated MukBEF clusters was observed in widefield imaging (Nicolas et al., 2014).

Here, we used super-resolution microscopy to characterize the behavior of single molecules of TopoIV in live *E. coli*. Moreover, by perturbing the action of TopoIV molecules using

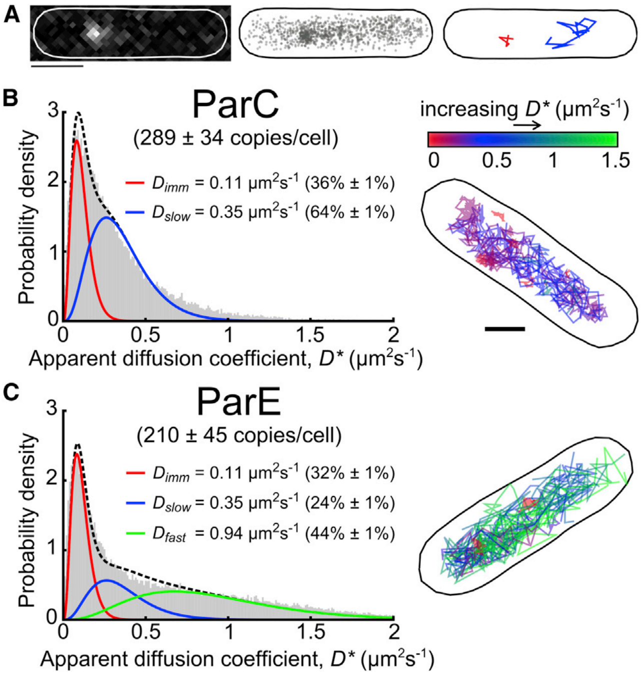

Figure 1. Tracking PALM of *E. coli* ParC/E Molecules

(A) Example image of a single ParC-PAmCherry molecule at 15 ms exposure (left), super-resolved localizations derived from all frames and for all molecules detected in this cell (middle), and example tracks of individual slow ParC (blue) and immobile ParC (red) molecules (right). Scale bar, 1 μm.

(B) Distribution of apparent diffusion coefficients (D^*) of 73,020 tracked ParC molecules, fitted with a two-species model. Ranges indicate 95% confidence interval. Example cell with individual trajectories colored according to their D^* value.

(C) Distribution of D^* values for 64,551 ParE molecules fitted with a three-species model. Copy numbers of ParC and ParE subunits, normalized for cells 2.5 μm long, were determined by sequentially photoactivating and tracking all available molecules.

genetics, an inhibitor, and overexpression of competing protein domains, we are able to provide mechanistic insight into the function of TopoIV and its interaction with MukBEF clusters. Using photoactivated-localization microscopy (PALM) combined with single-particle tracking (Manley et al., 2008), we show that ~60 molecules of TopoIV were present at any time, although sufficient ParC and ParE subunits were present for ~105 TopoIV molecules. Impairing the interaction between functional TopoIV and MukBEF, by overexpressing a competing but non-functional ParC C-terminal domain, resulted in an ~2-fold reduction in the number of immobile TopoIV molecules, consistent with the interaction between TopoIV and MukBEF directing the location and catalytic action of TopoIV molecules toward *ori*-associated MukBEF clusters. We identified two populations of immobile TopoIV molecules; we propose that one with a dwell time of ~1.8 s identifies catalytically active molecules, while the other, with a dwell time of ~30–70 ms, identifies molecules bound to MukBEF clusters. Wide-field, PALM, and 3D-structured illumination microscopy (3D-SIM) (Allen et al., 2014) demonstrated that MukBEF clusters were enriched along the long axis of the cell. Furthermore, we found that the MukB-ParC interaction, although not essential for TopoIV function, is crucial for timely segregation of newly replicated *ori* DNA. Impairing this interaction caused delayed segregation of newly replicated sister *oris*, consistent with the MukBEF-ParC interaction enhancing decatenation of newly replicated DNA.

RESULTS

A Fraction of TopoIV Subunits Are in TopoIV Heterotetramers

To characterize the copy number and behavior of TopoIV heterotetramers in live *E. coli* cells, we labeled the ParC or ParE subunits by replacing the endogenous genes with functional C-terminal fusions to the photoactivable fluorophore, PAmCherry. The fusions were fully functional in in vivo assays (Supplemental Experimental Procedures; Figure S1A; Table S3). Cells were imaged with a PALM microscope and individual molecules localized in each frame. Linking consecutive localizations into trajectories allowed us to follow the movement of individual ParC/E molecules at 15-ms intervals until photobleaching (Figure 1A) (Manley et al., 2008; Uphoff et al., 2013). 289 ± 34 photoactivatable molecules of ParC and 210 ± 46 photoactivatable molecules of ParE, normalized to a 2.5-μm-long cell, were counted. Since the photactivation efficiency of PAmCherry was determined to be ~50% in vivo, the actual copy numbers are likely to be approximately two times higher than these values (Supplemental Experimental Procedures).

To measure the mobility of ParC/E, we calculated an apparent diffusion coefficient (D^*) for each molecule from the one-step mean squared displacement (*MSD*) of its trajectory using $D^* = MSD/(4\ \Delta t)$, where Δt is the frame time of 15 ms. The different diffusing populations, which could not be described by a single diffusing species (Figure S1B), were defined by fitting an analytical expression to the distribution of experimental D^* values (Stracy et al., 2015). We first established the mean D^* of immobile molecules. Based on a localization error of ~40 nm, we estimated mean D^* of immobile molecules to be ~0.1 $\mu m^2 s^{-1}$. This was confirmed by fitting to the distribution D^* values for the previously characterized protein DNA polymerase 1 (where the immobile population was clearly resolvable), showing that $D_{imm} = 0.11 \pm 0.01\ \mu m^2 s^{-1}$ (Uphoff et al., 2013; Stracy et al., 2015; Figure S1C).

The ParC D^* distribution fitted well to a two-species model (Figure 1B): an immobile population (36% ± 1%; constrained at $D_{imm} = 0.11\ \mu m^2 s^{-1}$) and a second, unconstrained D distribution, corresponding to a slowly diffusing population (64% ± 1%; $D_{slow} = 0.35 \pm 0.01\ \mu m^2 s^{-1}$). Molecules in the slow-diffusing population had a lower mobility than expected for free 3D diffusion, consistent with them undergoing transient interactions with DNA, which ParC does (Corbett et al., 2005). The spatial distribution of slowly diffusing ParC molecules showed that they were associated with the nucleoid, consistent with them being transiently associated with DNA (Figures S1G and S3A). In contrast, we propose that the immobile ParC molecules are relatively stably bound to DNA or DNA-bound proteins.

The ParE D^* distribution showed a third population of molecules with higher mobility in addition to the two populations similar to those observed for ParC. As ParE does not bind DNA

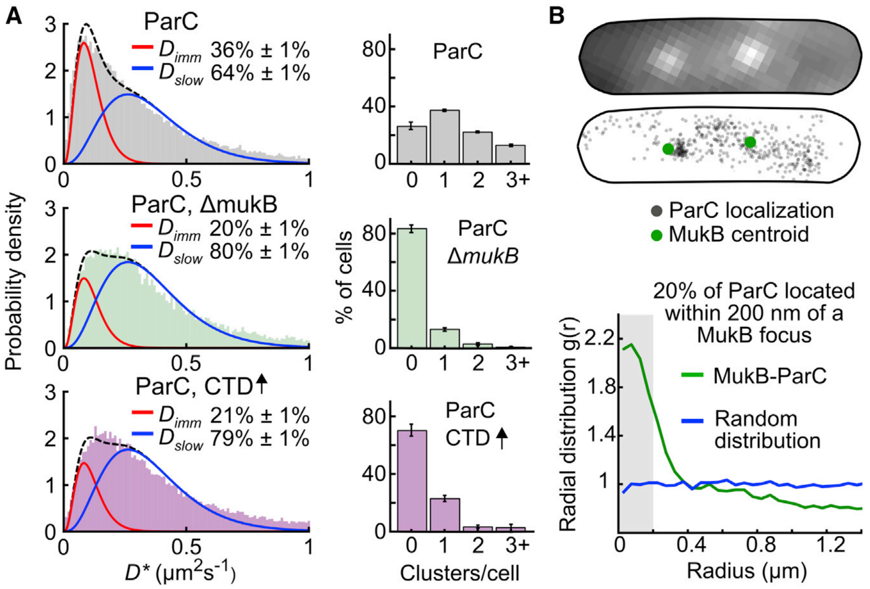

Figure 2. MukBEF Clusters Influence TopoIV Diffusion and Organization

(A) Left panels: distribution of D^* values for ParC molecules fitted with a two-species model with immobile molecules (constrained at D_{imm} = 0.11 $\mu m^2 s^{-1}$) and slow-moving molecules (constrained at D_{slow} = 0.35 $\mu m^2 s^{-1}$). Top: ParC molecules in wild-type cells (from Figure 1B). Middle: 18,971 ParC molecules in Δ*mukB* cells. Bottom: 42,920 ParC molecules after unlabeled ParC-CTD overexpression (3 hr). Ranges give 95% confidence intervals. Right panels: the number of ParC clusters per cell, determined by clustering all localizations using a nearest-neighbor algorithm, in wild-type (2,635 cells) and Δ*mukB* (387 cells) cells and with ParC-CTD overexpression (214 cells). Error bars indicate SD of three experimental repeats.

(B) Example cell with MukB-mYPet foci (top) visualized prior to PALM acquisition and localization of ParC-PAmCherry molecules (middle). Radial distribution of ParC localizations from each MukB focus (717 cells), compared to random distribution (bottom). The radial distribution function shows the probability of finding a ParC localization at distance, *r*, from a MukB focus. Gray bar shows localization within 200 nm.

(Lee et al., 2013), we propose that the fast-diffusing molecules represent free ParE subunits, whereas the immobile and slow-diffusing molecules were in TopoIV heterotetramers. To test this, we imaged ParE-PAmCherry molecules in cells in which unlabeled ParE was overexpressed, outcompeting labeled ParE in TopoIV heterotetramers. Consistent with our hypothesis, ~90% of ParE-PAmCherry molecules now diffused rapidly and were uniformly distributed throughout the cell, showing no bias toward the nucleoid region (Figures S1F and S1G). Fitting a three-species model to this data (with D_{imm} and D_{slow} constrained) established that D_{fast} = 0.94 ± 0.02 $\mu m^2 s^{-1}$. Conversely, imaging ParE-PAmCherry molecules in a strain overexpressing unlabeled ParC showed that the D_{fast} population is completely lost (Figure S1H), confirming that ParE molecules in the D_{imm} and D_{slow} states are complexed with ParC in TopoIV heterotetramers.

The three-species model for the ParE data, with constrained D_{imm}, D_{slow}, and D_{fast} values, showed that 32% ± 1% were immobile, 24% ± 1% were slow diffusing, and 44% ± 1% were fast diffusing (Figure 1C). As the copy-number estimates showed that there is no excess ParE in the cell, the 44% of uncomplexed, fast-diffusing ParE molecules must reflect a steady-state level of TopoIV heterotetramer formation and dissociation, with ~56% of ParE subunits being present in ~60 TopoIV heterotetramers. Therefore, the ~60 TopoIV molecules present at any time form from a pool of ParC and E molecules sufficient for ~105 TopoIV heterotetramers. By using the estimated copy numbers and the ~1.1 μm^3 volume of cells 2.5 μm long, we estimated the in vivo dissociation constant of TopoIV heterotetramers to be ~0.5 μM (Supplemental Experimental Procedures).

Half of Immobile TopoIV Molecules Result from Interaction with MukB

Since ParC interacts with the MukB dimerization hinge in vitro (Hayama and Marians, 2010; Li et al., 2010; Vos et al., 2013b) and shows an enrichment near MukBEF clusters in vivo (Nicolas et al., 2014), we tested whether a fraction of the immobile ParC and ParE molecules result from their binding to immobile MukBEF clusters on DNA. Fitting a two-species model (with the D_{imm} and D_{slow} populations established previously) to the distribution of D^* values for ParC molecules in Δ*mukB* or *mukB*^{DA} cells; MukB^{DA} is unable to bind ATP and form *ori*-associated MukBEF clusters (Badrinarayanan et al., 2012), showed a ~50% reduction in the immobile fraction of ParC/ParE consistent with these molecules being immobile as a consequence of their interaction with *ori*-associated MukBEF clusters (Figure 2A; Figures S2A and S2B). Using a clustering algorithm to define ParC clusters containing ≥25 localizations, we showed that ParC formed a median of one cluster per cell and deletion of MukB removed most ParC clustering (Figure 2A). This was confirmed by the radial distribution analysis of all ParC localizations that showed a strongly clustered distribution, which was reduced ~4-fold in Δ*mukB* cells (Figure S2C).

Since Δ*mukB* and *mukB*^{DA} cells have disorganized chromosomes (Danilova et al., 2007), we also considered whether the reduction in the fraction of immobile ParC/E molecules in these cells was instead a consequence of global chromosome changes. To distinguish these possibilities, we impaired the TopoIV-MukB interaction by overexpressing an unlabeled ParC C-terminal domain (ParC-CTD), which binds MukB (Vos et al., 2013b), thereby outcompeting TopoIV binding. Overexpression of ParC-CTD did not significantly affect growth rate, cell length, or formation of anucleate cells (Table S3), consistent with unperturbed chromosome organization. Flow cytometry profiles showed a small increase in cells with multiple chromosomes (Figure S1A). Under these conditions, the immobile fraction of ParC was reduced to the level in *ΔmukB* cells (Figure 2A, bottom), and clustering of ParC was lost, consistent with approximately half of immobile ParC molecules being

OPEN ACCESS
CellPress

dependent on a direct interaction with immobile MukBEF clusters.

To demonstrate that ParC clusters spatially associate with MukBEF clusters, we imaged ParC-PAmCherry and MukB-mYPet in the same cells. Calculating the radial distribution function of ParC PALM localizations with respect to the centroid of each MukBEF focus showed that ParC is enriched near MukBEF foci, which moved very little during the observation period (Figure 2B; Figure S2D), with ~20% of ParC localizations within 200 nm of MukBEF centroids. This result is consistent with the ~16% of ParC molecules that were immobile due to a direct interaction with MukB, as judged by the reduction in the fraction of immobile molecules in *ΔmukB* cells (Figure 2A). We noticed that while ParC clusters were nearly always in close proximity to a MukBEF focus, not all MukBEF foci were associated with a ParC cluster, a trend also evident in intensity projections from epifluorescent imaging (Figure S3E). Imaging MukB-PAmCherry with PALM showed that, despite having a similar copy number (195 ± 57 copies/cell; Figure S2F), MukBEF formed approximately twice as many clusters per cell as ParC (Figure S2G), thereby indicating an additional level of regulation governing the MukB-ParC interaction.

By using the fraction of immobile TopoIV molecules dependent on MukB (Figures 1B, 1C, and 2A) and, independently, the fraction of ParC localizations close to MukB in the radial distribution analysis (Figure 2B), we estimated that ~15 TopoIV molecules were associated with MukBEF clusters at any given time and determined the in vivo dissociation constant of MukB-ParC complexes to be ~2 μM (Supplemental Experimental Procedures), consistent with in vitro measurements (Li et al., 2010).

To understand further how MukBEF clusters direct the organization of immobile ParC molecules within the nucleoid, we determined the probability density of ParC molecules across the short cell axis. We segmented cell outlines from the bright-field images and determined the intracellular location of the tracks. We then established a D^* threshold (0.16 $\mu m^2 s^{-1}$), which preserved the ratio of immobile (36%) to mobile (64%) molecules, established from fitting, to classify each individual ParC track as immobile or mobile. The analysis showed that immobile ParC molecules were preferentially located along the long axis of the cell (Figure S3A). Similar intracellular positioning was observed for immobile MukB-PAmCherry molecules, with an even stronger bias of immobile molecules along the long cell axis (Figure S3A, right). We found a similar pattern of MukBEF cluster enrichment on the long cell axis when we analyzed the distribution of MukBEF foci in epifluorescence images (Figure S3B). In Δ*mukB* cells, immobile ParC molecules showed a lower probability of locating to the cell long axis, consistent with MukBEF clusters recruiting ParC molecules to the long cell axis (Figure S3A, middle). When we co-imaged MukB-mYPet and DAPI-stained DNA with 3D structured illumination microscopy, we also observed MukBEF clusters located along the long cell axis, close to regions of high nucleoid density (Figure S3C; Movie S1; Supplemental Experimental Procedures).

Two Populations of Immobile TopoIV Molecules

To dissect TopoIV binding events, we analyzed long trajectories of ten or more localizations and sorted molecules into three categories: mobile molecules that remained above the D^* threshold for the observation period, immobile molecules that remained below the D^* threshold over the observation period, and molecules that exhibited transitions between these states (Figure 3A). This analysis detected similar fractions of immobile molecules as determined from fits to the D^* distributions (compare Figure 3B with Figure 1B). In addition, a fraction of the molecules underwent transitions, consistent with TopoIV molecules being in a dynamic equilibrium between bound and mobile states (Figure 3A, right).

In time-lapse experiments, using 15-ms exposures followed by 35-ms delays, we observed a reduction in the population of ParC molecules that remained immobile over the course of the trajectory from 35% to 14% (Figure 3B). The result was also evident in D^* distributions (Figure S4A). Molecules in the immobile category in the time-lapse experiments (bound for ten or more localizations with a 50-ms frame time) must remain bound for ≥0.5 s, compared to ≥0.15 s for the immobile molecules in normal 15-ms frame-time experiments (bound for ten or more localizations). The observed reduction in the fraction of immobile molecules shows that ~21% of the binding events in Muk^+ cells lasted for ≤0.5 s. In contrast, when we performed the same analysis in Δ*mukB* cells or in cells overexpressing ParC-CTD, the fraction of immobile molecules remained unchanged in normal and time-lapse PALM experiments (Figure 3B). This shows that in wild-type cells, a population of MukB-dependent transiently immobile (≤0.5 s) TopoIV molecules is present alongside molecules immobile for ≥0.5 s.

Because the underlying binding times are exponentially distributed, they cannot be extracted intuitively from experiments. We therefore used Markov chain Monte Carlo simulations to gain a better estimate of the durations of the short-lived MukB-dependent binding events. Molecule trajectories were simulated undergoing Brownian motion inside a confined cell volume (Bakshi et al., 2013; Persson et al., 2013; Uphoff et al., 2013). Molecules were in one of two diffusive states: D_{free} and D_{imm}, with transitions allowed between each state. The free diffusion, D_{free}, of TopoIV heterotetramers was calculated based on the free diffusion of ParE (D_{fast}), correcting for their relative sizes (Supplemental Experimental Procedures). Molecule trajectories were simulated to generate localizations at either 15-ms intervals or 15-ms intervals with 35-ms delays to match normal and time-lapse experiments, respectively. The simulated localizations were analyzed with the same tracking and categorizing algorithm as used for the experimental data. We simulated interconverting molecules with different exponentially distributed binding durations from 0.1 ms to 150 ms (keeping the fraction in each state equal). Plotting the change in the fraction of molecules categorized as bound in time-lapse simulations compared to normal simulations showed that a binding duration of 30–70 ms for MukB-dependent TopoIV transient binding events recapitulated the experimentally observed decrease (Figure 3C; Figure S4D). Furthermore, simulations with a binding time ≪ exposure time showed that a transient (≤1 ms) DNA binding explains well the lower-than-expected mobility of slowly diffusing ParC molecules (Figure S4C).

Finally, we characterized the molecules that remained immobile over the time-lapse experiment observation time (binding

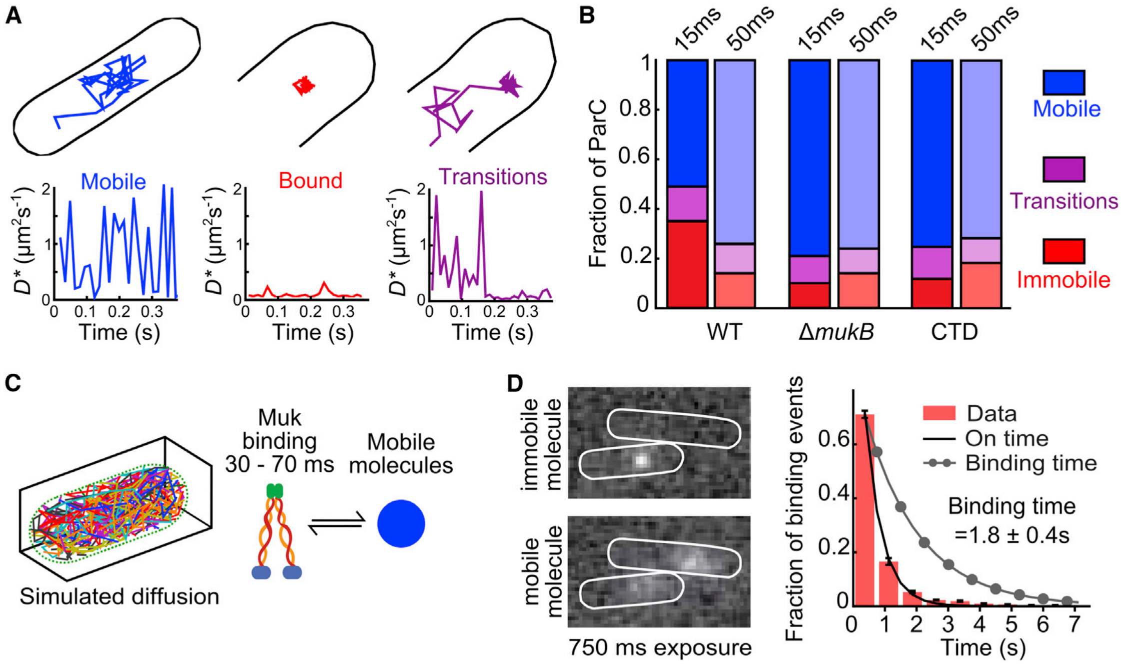

Figure 3. MukB-Dependent and Independent ParC Binding Behavior

(A) Examples of long ParC trajectories (ten or more localizations) classified according to their D^* transitions. Molecules mobile over observation period (blue), immobile (red), and undergoing transition from one state to another (purple).

(B) Bar graph of all ParC trajectories for the indicated strains, classified from PALM experiments performed at 15-ms continuous acquisition and time lapse (15-ms exposure + 35-ms delay).

(C) Schematic of Markov chain Monte Carlo simulations of molecules inside a typically sized cell volume interconverting between immobile and free diffusion. Cartoon representation of transitions analyzed in simulations. Shown is the time range obtained in simulations that recapitulated the experimental data.

(D) Left: example 750-ms exposure frames showing cells with an immobile TopoIV molecule (top) and a mobile molecule (bottom). Right: on-time distributions for immobile ParC with exponential fit (line) and photobleaching-corrected binding time distribution (line with dots). Error bars indicate SD of three experimental repeats.

time ≥0.5 s). Because our ability to observe complete events was limited by photobleaching, we increased the observation time by using low excitation intensities, sparse photoactivation, and long (≥0.5 s) exposure times, when mobile molecules are motion blurred, whereas immobile molecules appear as point sources, producing a diffraction-limited spot (Elf et al., 2007; Stracy et al., 2014) (Figure 3D). Immobile molecules could therefore be distinguished by the width of the elliptical Gaussian fits to the fluorescent spot. We used thresholds established with Pol1 (with clearly resolvable immobile molecules) of <160 nm short axis width and <200 nm long axis width to identify immobile molecules (Uphoff et al., 2013; Figure S4E). The probability of observing a particular on-time is the product of the underlying binding-time probability and the bleaching probability. The bleaching-time distributions were measured independently with the same acquisition and excitation conditions using MukB-PAmCherry, which binds DNA in clusters with a dwell time longer (~50 s) than the photobleaching lifetime (Badrinarayanan et al., 2012). We measured ParC on-times at 0.5 s, 0.75 s, and 1 s exposure times and corrected for photobleaching (Uphoff et al., 2013). We found the mean binding time to be 1.8 ± 0.4 s (Table S4).

In conclusion, we have shown that "immobile" TopoIV molecules display two different bound states: a 30- to 70-ms MukBEF binding-dependent state and ~1.8-s binding events, which we propose identify TopoIV molecules undergoing a single catalytic cycle, since such a binding time is of the same order as measurements of a single TopoIV catalytic cycle in vitro (Crisona et al., 2000; Neuman et al., 2009; Stone et al., 2003). The analysis did not detect longer events that would be expected for processive catalysis. Based on analysis in vitro of processive bursts on (+) supercoiled DNA, they were expected to last tens of seconds (Crisona et al., 2000; Stone et al., 2003).

TopoIV Molecules Undergoing Catalysis Are Enriched at MukBEF Clusters

To determine if the TopoIV-MukB interaction directs TopoIV catalytic activity close to MukBEF clusters, we treated cells carrying a norfloxacin-resistant gyrase gene with norfloxacin, which blocks the TopoIV catalytic cycle, resulting in ParC molecules covalently bound to DNA (Khodursky et al., 1995). We observed a 2-fold increase in the fraction of immobile ParC/E molecules after ~10-min norfloxacin treatment (Figure 4A; Figure S5A), showing that most TopoIV molecules had performed catalysis during this period; however, we cannot exclude the possibility that norfloxacin captures a fraction of nonproductive catalytic events that do not result in topological changes. Longer incubation with norfloxacin did not increase the fraction of immobile

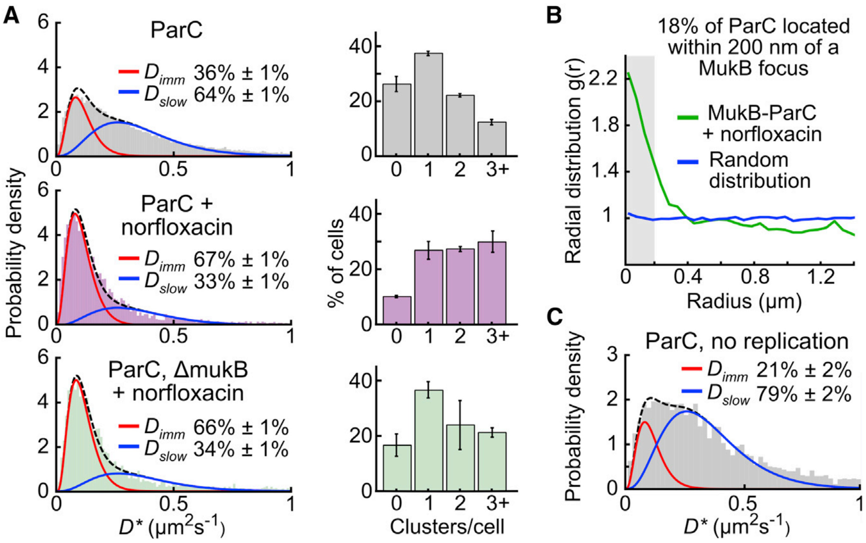

Figure 4. Catalytically Active TopoIV

(A) Left panels: distribution of D^* values for ParC molecules in wild-type (387 cells) and Δ*mukB* (214 cells) cells after ~10-min treatment with norfloxacin. Control ParC molecules in untreated cells (top, from Figure 1B). Right panels: number of ParC clusters per cell for steady-state populations of cells. Error bars indicate SD of three experimental repeats. Ranges give 95% confidence intervals.

(B) Radial distribution of ParC localizations from each MukB focus in cells treated with norfloxacin (726 cells), compared to random distribution.

(C) Distribution of D^* values for 1,930 ParC molecules in non-replicating cells, as assessed by lack of mYPet-DnaN foci prior to PALM data acquisition. Distributions of D^* were fitted with a two-species model with both D values constrained.

molecules, showing that at ~10 min, we had reached saturation and did therefore not have a quantitative measure of catalytic rate; shorter exposure times were not experimentally tractable. Δ*mukB* cells showed a similar fraction of immobile molecules after saturating norfloxacin treatment. This result agrees with the fact that Δ*mukB* cells can decatenate and segregate their chromosomes (Danilova et al., 2007; Nicolas et al., 2014), but it does not address the question of whether the MukB-ParC interaction stimulates decatenation globally. The enrichment of ParC molecules close to MukBEF clusters, as judged by radial distribution analysis, was retained after norfloxacin treatment (Figure 4B), showing that a fraction of TopoIV molecules underwent catalysis close to MukBEF clusters. After norfloxacin treatment of wild-type cells, we observed a modest increase in the number of TopoIV clusters per cell, whereas Δ*mukB* cells showed a similar cluster distribution to wild-type cells (Figure 4A). These data indicate that TopoIV molecules undergo catalysis in defined clusters, some of which are close to MukBEF clusters.

When we analyzed the distribution of catalytically active TopoIV molecules across the short cell axis, we found that the previous bias toward the cell center was lost, presumably because the additional clusters were not associated with MukBEF clusters (Figure S5C). MukBEF clusters were retained after norfloxacin, and ParC colocalized with them (Figure S5B). In the absence of MukBEF clusters, covalently linked DNA-ParC molecules were less likely to be located close to the long cell axis, similar to the situation in steady-state cells (compare Figure S5C curve with Figure S3A middle curve). Taken together, the results indicate that MukBEF clusters direct the catalytic activity of some TopoIV molecules to the cell long axis, whereas MukBEF-independent catalysis occurs throughout the nucleoid. In these analyses, we cannot exclude the possibility that covalently bound TopoIV reshapes the chromosome and thereby influences the spatial distribution of TopoIV. Nevertheless, the spatial distribution of MukBEF clusters was retained, suggestive of normal chromosome organization being maintained. Furthermore, we note that TopoIV-targeted strand breaks introduced by norfloxacin did not lead to chromosome fragmentation (Hsu et al., 2006).

TopoIV Catalysis in Cells Lacking (Pre)catenanes

To address whether TopoIV catalysis occurs in cells lacking (pre) catenanes, we analyzed cells from a steady-state population that had not initiated DNA replication, as assessed by a lack of mYPet-DnaN foci. The reduction in the immobile fraction of ParC from 36% ± 1% to 21% ± 2% in these cells, when compared to the whole population (Figure 4C), indicated that almost half of immobile TopoIV molecules were dependent on replication. The replication-independent molecules showed a similar cluster distribution to that in steady-state cells (Figure S5D), consistent with a large fraction of them being bound to MukBEF clusters. Norfloxacin treatment gave a similar proportion of immobile TopoIV molecules as in steady-state cells, showing that TopoIV catalysis occurs in the absence of (pre)catenanes, but not addressing its frequency (Figure S5E). We conclude that even though the essential function of TopoIV is in decatenation, its catalytic action is not restricted to (pre) catenanes.

The Interaction between ParC and MukB Facilitates *ori* Decatenation

To test whether the interaction between ParC and *ori*-associated MukBEF clusters influenced decatenation of newly replicated *ori*-sisters, we used two assays to assess the time of *ori* separation after replication, and we analyzed how this changed after impairment of the ParC-MukBEF interaction. These assays have been validated previously and have shown that the time of locus separation is regulated by the activity/availability of TopoIV, indicating that decatenation by TopoIV directs the time of chromosome segregation (Joshi et al., 2013; Wang et al., 2008).

In time-lapse experiments, we measured the time of *ori1* locus separation after replication initiation, marked by the appearance of a fluorescent mYPet-DnaN focus (Figure 5A). The *ori1* locus replicates <30 s after initiation at *oriC*, and a sufficient amount

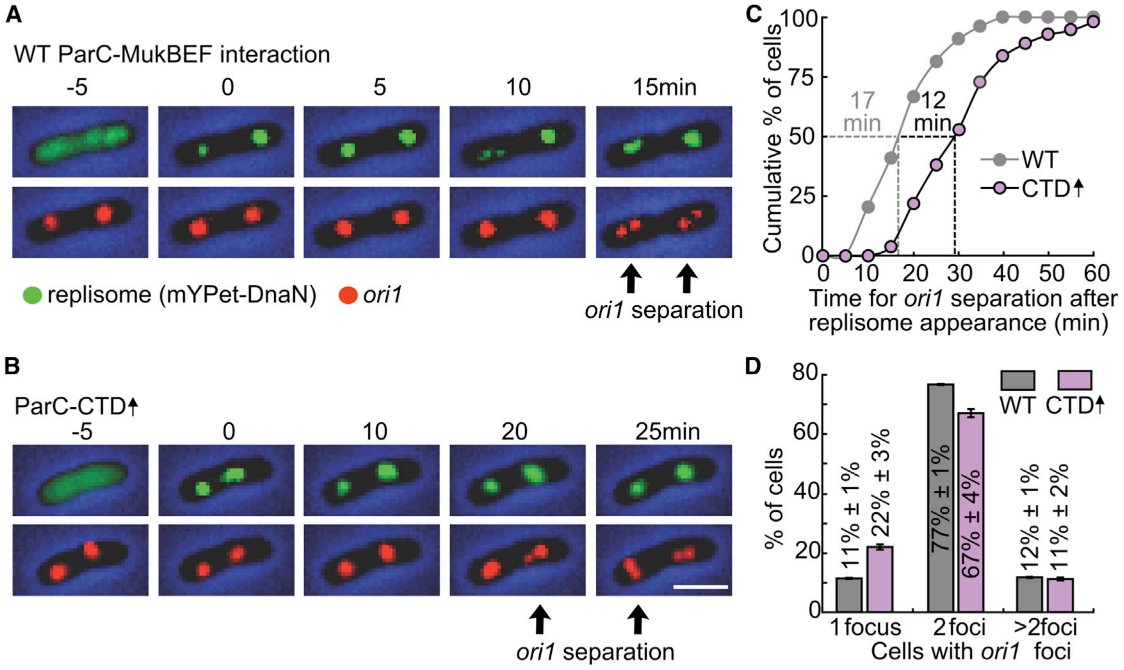

Figure 5. The MukB-ParC Interaction Stimulates *ori* Decatenation

(A) Example cells from the time-lapse experiments with wild-type cells transformed with empty expression plasmid (pBAD24). Black arrows show time of *ori1* segregation. 0 min time was defined by replisome appearance at *ori1*.

(B) Example cell showing ParC-CTD 3-hr overexpression.

(C) Cumulative distribution of times of *ori1* locus segregation after replication initiation, marked by appearance of mYPet-DnaN foci at *ori1*.

(D) Snapshot analysis of the number of *ori1* foci/cell in steady-state cells. Mean ± SD of three independent experiments (>1,000 cells).

of mYPet-DnaN loads at the forks to visualize it within <2 min of initiation (Moolman et al., 2014). Cells in which the MukBEF-ParC interaction is normal had stably segregated 50% of the newly replicated *ori1* loci by 17 min after the appearance of mYPet-DnaN. In contrast, cells in which the MukBEF-ParC interaction was impaired by ParC-CTD overexpression showed a ~12 min increase in the time required for 50% of cells to exhibit *ori1*-segregation (Figures 5B and 5C).

We also compared the fraction of cells containing one or more *ori1* foci in snapshots of steady-state populations (Figure 5D). We observed a decrease in the fraction of cells containing two *ori1* foci when the ParC-MukBEF interaction was impaired, consistent with delayed *ori1* decatenation. Taken together, these results provide strong support for a mechanism in which the MukB-TopoIV interaction plays a role in timely decatenation of newly replicated *ori*1 DNA.

DISCUSSION

The in vivo single-molecule approach exploited here provides a comprehensive understanding of the formation and behavior of TopoIV molecules in their native unperturbed environment inside living cells. We observed a dynamic equilibrium between free and complexed ParC/E subunits, independently of replication, with ~60 potentially functional TopoIV heterotetramers at birth. The observation that only ~60% of subunits are in heterotetramers at any given time raises the possibility that more functional enzymes can be assembled if required. By combining in vivo super-resolution techniques with genetics, inhibitors, and overexpression of competing proteins, we have gained a molecular understanding of the interaction between TopoIV and MukB and demonstrated the important functional significance of this interaction for DNA segregation. We are confident that the C-terminal fusions of ParC and ParE are fully functional, that the fluorescent protein domains themselves are not influencing the localization and diffusional properties of the fusion proteins and therefore that the observed behavior reflects the true properties of TopoIV subunits (Supplemental Experimental Procedures).

Catalysis by TopoIV

We infer that individual TopoIV molecules undergo multiple attempts to bind DNA productively before undergoing catalysis. This is reflected in the slow diffusion of ParC/TopoIV molecules, which simulations have indicated may result from transient (≤1 ms) interactions with DNA (Figure 1B; Figure S4C). We infer that this transient binding probably does not identify molecules undergoing catalysis, since a single TopoIV catalytic cycle was measured to be ~1 s in single-molecule and ensemble experiments in vitro (Crisona et al., 2000; Neuman et al., 2009; Stone et al., 2003). Our analysis is consistent with other data (Lee et al., 2013; Stone et al., 2003; Vos et al., 2013a) that has led to the proposal that prior to catalysis, TopoIV must first capture the G-DNA segment that is to be cleaved and then capture a

T-segment that is transported through the cleaved G-segment. The transient DNA binding we observed likely represents an initial interaction with DNA proceeding G-segment capture or G-segment capture itself. Assuming that long binding events, lasting ~1.8 s ($t_{catalysis}$, Figure 3D), represent catalytically active molecules and that 14% ($F_{catalysis}$) of all molecules display this behavior (Figure 3B), we calculated the mean time for a given TopoIV molecule to locate and productively bind to its substrate, t_{search}, using $F_{catalysis} = t_{catalysis}/(t_{catalysis} + t_{search})$ (Uphoff et al., 2013). We calculated that ($t_{search} + t_{catalysis}$) = ~13 s. Therefore, for ~11 s, TopoIV molecules will diffuse slowly, presumably undergoing multiple transient interaction with DNA, before initiating a catalytic cycle.

(−) supercoils are relaxed distributively in vitro, whereas (+) supercoils are relaxed processively (Crisona et al., 2000; Stone et al., 2003). Given that processive events would be predicted to last tens of seconds, the inferred TopoIV catalytic cycle of 1.8 s, derived from the long exposure analysis, suggests that TopoIV predominantly acts distributively rather than processively in vivo. This is consistent with RH (−) supercoils and RH replicative catenanes being the preferred targets for TopoIV.

Our results provide a deeper understanding of the relative activity of TopoIV on (pre)catenanes behind replication forks and in maintaining global supercoiling homeostasis. TopoIV-mediated decatenation behind the fork is essential, and TopoIV inactivation prevents decatenation and segregation of newly replicated sister loci without affecting replication or transcription (Wang et al., 2008). DNA gyrase is thought to be largely responsible for removal of LH (+) supercoils ahead of replication forks and the transcription machinery (Vos et al., 2011). Our results showing a reduction of immobile TopoIV molecules in non-replicating cells, and a failure to detect processive relaxation of (+) supercoils in vivo, are consistent with most TopoIV activity being directed to decatenation of newly replicated DNA. Consistent with this, covalent linking of TopoIV to DNA with norfloxacin does not block chromosomal or plasmid replication (Khodursky and Cozzarelli, 1998; Khodursky et al., 1995). In the absence of functional gyrase, TopoIV could support replication at ~30% of the wild-type rate (Khodursky et al., 2000), indicating that TopoIV may act ahead of the fork, although it could be that in the absence of gyrase accumulation of the (+) supercoiling ahead of the fork is converted intro precatenanes by replisome rotation, thereby allowing limited fork progression as a consequence of TopoIV-mediated decatenation.

The presence of a similar proportion of TopoIV heterotetramers in cells of all ages (Figures S5F–S5H) and catalytically active TopoIV molecules in cells that have not initiated replication, along with the demonstration that TopoIV availability controls decatenation at *ori* (Figure 5) (Joshi et al., 2013; Wang et al., 2008; Nicolas et al., 2014), shows that TopoIV activity is not directed exclusively to replication termination and is inconsistent with the hypothesis that active TopoIV molecules form only at replication termination as a consequence of replisome disassembly (Espeli et al., 2003).

MukBEF Cluster-Dependent TopoIV Molecules

Our data lead us to propose that direct interaction between ParC and MukB leads to a fraction of TopoIV molecules being bound to MukBEF clusters for 30–70 ms, unless they undergo catalysis, in which case we propose that they will remain bound for ~1.8 s. The MukBEF cluster-ParC interaction is important for timely segregation of newly replicated sister *ori*s, consistent with the observation that TopoIV availability determines *ori1* locus separation time (Wang et al., 2008). Furthermore, ablation of TopoIV activity, prevents *ori* segregation and its reinstatement leads to resumed *ori* segregation (Nicolas et al., 2014). These observations strongly suggest that measurements of *ori* segregation time define decatenation efficiency. In the experiments here, we have demonstrated an ~12-min delay in *ori1* segregation if the ParC-MukB interaction is impaired, consistent with TopoIV being less active in decatenation without this interaction. We propose the TopoIV interaction with MukBEF clusters may favor *ori* decatenation partly because of an increased local concentration of TopoIV and partly because of enhanced catalysis. Because the MukB-ParC interaction stimulates relaxation of RH (−) supercoils in vitro, we would also expect this interaction to stimulate decatenation because of the identical chirality of replicative catenanes and negative supercoils (Nicolas et al., 2014). Although in vitro experiments designed to test whether the TopoIV-MukB interaction stimulated decatenation showed little or no stimulation (Hayama et al., 2013; Hayama and Marians, 2010; Li et al., 2010), the substrates used were different from those used in the supercoil relaxation experiments. We propose, given our in vivo results and the identical chirality of replicative catenanes and (−) supercoils, that the TopoIV-MukBEF interaction will stimulate decatenation. Since the MukBEF clusters are relatively stably associated with DNA (Badrinarayanan et al., 2012), their interaction with TopoIV may facilitate binding of the G- and/or T-segment by TopoIV. Alternatively, this interaction might affect TopoIV substrate specificity.

Since decatenation of newly replicated *ori*s only occurs during a short period of the cell cycle, we wonder also whether the MukBEF cluster-TopoIV interaction may stimulate (−) supercoil relaxation in the region of the origin and thereby act to prevent premature *ori* firing, which requires that *ori* is highly negatively supercoiled (Donczew et al., 2014). Consistent with this, we note that MukBEF clusters tended to move away from *ori* prior to replication initiation (Nicolas et al., 2014) and that in cells in which the TopoIV-MukB interaction is perturbed, we observed some replication initiation asynchrony (Figure S1A).

Perspective

We propose that the coordination of type II topoisomerase activity by an SMC complex revealed here is not limited to *E. coli*. Other studies have implicated functional interactions between eukaryotic SMCs and their TopoIV counterpart, TopoII (Baxter, 2015; Baxter and Aragón, 2012). For example, condensin was shown to facilitate decatenation of yeast minichromosomes (Charbin et al., 2014). The sequential and coordinated action of TopoIV and MukBEF in the successive steps of decatenation and chromosome segregation revealed here provides a platform for future mechanistic studies that will reveal whether SMC complexes provide DNA-protein substrates that provide selectivity for topoisomerase action and precisely how topoisomerase action is coordinated with SMC functions in chromosome processing.

EXPERIMENTAL PROCEDURES

Bacterial Strains and Growth

Bacterial strains are listed in Table S1. Plasmids and oligonucleotides are shown in Table S2. Strains were streaked onto Luria-Bertani broth plates with appropriate antibiotics. Single colonies were inoculated into M9 glycerol (0.2%) and grown overnight at 37°C to A_{600} 0.4–0.6, then diluted into fresh M9 and grown to A_{600} 0.1. Cells were centrifuged and immobilized on agarose pads between two glass coverslips (0.17 mm thick, heated to 500°C for 1 hr to remove any fluorescent background particles). We prepared 1% agarose pads by mixing low-fluorescence 2% agarose (Bio-Rad) in dH_2O 1:1 with 2× growth medium. For details, see Supplemental Experimental Procedures.

PALM Imaging, Molecule Localization, Tracking, and Diffusion

Live cell single-molecule-tracking PALM used a custom-built total internal reflection fluorescence microscope. Photoactivatable mCherry activation used a 405-nm laser, with excitation at 561 nm. YPet excitation was with a 488 nm laser. Bright-field cell images were recorded with an LED source and condenser (ASI Imaging). PALM single-molecule-tracking analysis used custom-written MATLAB software (MathWorks). We distinguished bound and diffusing proteins by calculating an apparent diffusion coefficient $D^* = MSD/(4\ \Delta t)$ from the mean-squared displacement (MSD) for each track with four steps. Note that D^* is an apparent diffusion coefficient because of cell confinement and motion blurring (Stracy et al., 2014). For details, see Supplemental Experimental Procedures.

Measuring Long-Lasting Binding Events

PALM movies to measure long-duration binding events were recorded at low continuous 561-nm excitation intensities using long exposure times (Uphoff et al., 2013). At these exposure times, mobile ParC-PAmCherry molecules are motion blurred over a large fraction of the cell, whereas immobile ParC-PAmCherry molecules still appear as point sources, producing a diffraction limited spot. The probability of observing a particular on-time is the product of the underlying binding-time probability and the bleaching probability. The bleaching-time distributions were measured independently using MukB-PAmCherry, which binds DNA in one to three large clusters per cell with a dwell time of ~50 s (Badrinarayanan et al., 2012), with the same acquisition and excitation conditions. On-time and bleaching-time distributions were fitted with single-exponential functions to extract exponential-time constants t_{on} and t_{bleach}, and the binding-time constant was calculated by $t_{bound} = t_{on} \times t_{bleach} / (t_{bleach} - t_{on})$.

Simulations

Diffusion simulations were performed with custom-written MATLAB software (MathWorks). Molecules were simulated undergoing Brownian motion confined within a volume corresponding to the average size of cells imaged in experiments. The localization in each 15-ms frame determined from averaging the simulated molecule positions over 100 subframes and adding Gaussian distributed localization error. The list of simulated localizations, with their corresponding frame number, could then be analyzed in exactly the same way as the experimental data.

Measuring Cohesion Time

Sister *ori1* cohesion time in the strain KG52 containing plasmid pZ68 (overproducing a ParC CTD domain) was assessed in a 5-min time-lapse analysis. We have measured the time from replisome appearance at initiation to *ori1* segregation. Chromosomal genetic loci were visualized using fluorescent repressor-operator systems. A *lacO* array was inserted 16 kb counterclockwise of oriC (*ori1)*; LacI-mCherry was expressed from the chromosomal *leuB* locus, regulated by the *lac* promoter (Wang et al., 2008). A chromosomally encoded mYPet-DnaN fusion protein was used as a marker for the replisome (Moolman et al., 2014; Reyes-Lamothe et al., 2010). Cells were growing exponentially in minimal medium supplemented with glycerol, at 37°C (generation time ~100 min). CTD overproduction was induced by addition of L-arabinose (final concentration, 0.2%) 3 hr prior to the experiment. As a control, the strain with the empty plasmid pBAD24 (Guzman et al., 1995) was used.

SUPPLEMENTAL INFORMATION

Supplemental Information includes Supplemental Experimental Procedures, five figures, four tables, and one movie and can be found with this article online at http://dx.doi.org/10.1016/j.celrep.2015.11.034.

AUTHOR CONTRIBUTIONS

P.Z. and D.J.S. designed the research. P.Z., K.G., K.Z., and C.L. performed experiments and analyzed data. M.S. wrote analytical tools and analyzed data. A.N.K provided technical advice. P.Z., M.S., and D.J.S. wrote the paper.

ACKNOWLEDGMENTS

Research in the Sherratt laboratory was supported by the Wellcome Trust (SIA 099204/Z/12Z) and the Leverhulme Trust (RP2013-K-017). A Wellcome Trust Strategic Award (Micron; 091911) for advanced microscopy provided equipment and expertise in imaging. M.S. was supported by an EPSRC studentship. We thank Stephan Uphoff, Charl Moolman, and Thomas Grigoris for helpful discussions.

Received: June 2, 2015
Revised: October 23, 2015
Accepted: November 10, 2015
Published: December 10, 2015

REFERENCES

Allen, J.R., Ross, S.T., and Davidson, M.W. (2014). Structured illumination microscopy for superresolution. Chemphyschem *15*, 566–576.

Badrinarayanan, A., Reyes-Lamothe, R., Uphoff, S., Leake, M.C., and Sherratt, D.J. (2012). In vivo architecture and action of bacterial structural maintenance of chromosome proteins. Science *338*, 528–531.

Bakshi, S., Dalrymple, R.M., Li, W., Choi, H., and Weisshaar, J.C. (2013). Partitioning of RNA polymerase activity in live Escherichia coli from analysis of single-molecule diffusive trajectories. Biophys. J. *105*, 2676–2686.

Baxter, J. (2015). "Breaking up is hard to do": the formation and resolution of sister chromatid intertwines. J. Mol. Biol. *427*, 590–607.

Baxter, J., and Aragón, L. (2012). A model for chromosome condensation based on the interplay between condensin and topoisomerase II. Trends Genet. *28*, 110–117.

Charbin, A., Bouchoux, C., and Uhlmann, F. (2014). Condensin aids sister chromatid decatenation by topoisomerase II. Nucleic Acids Res. *42*, 340–348.

Corbett, K.D., Schoeffler, A.J., Thomsen, N.D., and Berger, J.M. (2005). The structural basis for substrate specificity in DNA topoisomerase IV. J. Mol. Biol. *351*, 545–561.

Crisona, N.J., Strick, T.R., Bensimon, D., Croquette, V., and Cozzarelli, N.R. (2000). Preferential relaxation of positively supercoiled DNA by E. coli topoisomerase IV in single-molecule and ensemble measurements. Genes Dev. *14*, 2881–2892.

Danilova, O., Reyes-Lamothe, R., Pinskaya, M., Sherratt, D., and Possoz, C. (2007). MukB colocalizes with the oriC region and is required for organization of the two Escherichia coli chromosome arms into separate cell halves. Mol. Microbiol. *65*, 1485–1492.

Donczew, R., Zakrzewska-Czerwińska, J., and Zawilak-Pawlik, A. (2014). Beyond DnaA: the role of DNA topology and DNA methylation in bacterial replication initiation. J. Mol. Biol. *426*, 2269–2282.

Elf, J., Li, G.W., and Xie, X.S. (2007). Probing transcription factor dynamics at the single-molecule level in a living cell. Science *316*, 1191–1194.

Espeli, O., Levine, C., Hassing, H., and Marians, K.J. (2003). Temporal regulation of topoisomerase IV activity in E. coli. Mol. Cell *11*, 189–201.

Grainge, I., Bregu, M., Vazquez, M., Sivanathan, V., Ip, S.C., and Sherratt, D.J. (2007). Unlinking chromosome catenanes in vivo by site-specific recombination. EMBO J. *26*, 4228–4238.

Guzman, L.M., Belin, D., Carson, M.J., and Beckwith, J. (1995). Tight regulation, modulation, and high-level expression by vectors containing the arabinose PBAD promoter. J. Bacteriol. *177*, 4121–4130.

Hayama, R., and Marians, K.J. (2010). Physical and functional interaction between the condensin MukB and the decatenase topoisomerase IV in Escherichia coli. Proc. Natl. Acad. Sci. USA *107*, 18826–18831.

Hayama, R., Bahng, S., Karasu, M.E., and Marians, K.J. (2013). The MukB-ParC interaction affects the intramolecular, not intermolecular, activities of topoisomerase IV. J. Biol. Chem. *288*, 7653–7661.

Hirano, T. (2006). At the heart of the chromosome: SMC proteins in action. Nat. Rev. Mol. Cell Biol. *7*, 311–322.

Hsu, Y.H., Chung, M.W., and Li, T.K. (2006). Distribution of gyrase and topoisomerase IV on bacterial nucleoid: implications for nucleoid organization. Nucleic Acids Res. *34*, 3128–3138.

Joshi, M.C., Magnan, D., Montminy, T.P., Lies, M., Stepankiw, N., and Bates, D. (2013). Regulation of sister chromosome cohesion by the replication fork tracking protein SeqA. PLoS Genet. *9*, e1003673.

Khodursky, A.B., and Cozzarelli, N.R. (1998). The mechanism of inhibition of topoisomerase IV by quinolone antibacterials. J. Biol. Chem. *273*, 27668–27677.

Khodursky, A.B., Zechiedrich, E.L., and Cozzarelli, N.R. (1995). Topoisomerase IV is a target of quinolones in Escherichia coli. Proc. Natl. Acad. Sci. USA *92*, 11801–11805.

Khodursky, A.B., Peter, B.J., Schmid, M.B., DeRisi, J., Botstein, D., Brown, P.O., and Cozzarelli, N.R. (2000). Analysis of topoisomerase function in bacterial replication fork movement: use of DNA microarrays. Proc. Natl. Acad. Sci. USA *97*, 9419–9424.

Koster, D.A., Crut, A., Shuman, S., Bjornsti, M.A., and Dekker, N.H. (2010). Cellular strategies for regulating DNA supercoiling: a single-molecule perspective. Cell *142*, 519–530.

Lee, I., Dong, K.C., and Berger, J.M. (2013). The role of DNA bending in type IIA topoisomerase function. Nucleic Acids Res. *41*, 5444–5456.

Li, Y., Stewart, N.K., Berger, A.J., Vos, S., Schoeffler, A.J., Berger, J.M., Chait, B.T., and Oakley, M.G. (2010). Escherichia coli condensin MukB stimulates topoisomerase IV activity by a direct physical interaction. Proc. Natl. Acad. Sci. USA *107*, 18832–18837.

Manley, S., Gillette, J.M., Patterson, G.H., Shroff, H., Hess, H.F., Betzig, E., and Lippincott-Schwartz, J. (2008). High-density mapping of single-molecule trajectories with photoactivated localization microscopy. Nat. Methods *5*, 155–157.

Moolman, M.C., Krishnan, S.T., Kerssemakers, J.W., van den Berg, A., Tulinski, P., Depken, M., Reyes-Lamothe, R., Sherratt, D.J., and Dekker, N.H. (2014). Slow unloading leads to DNA-bound β2-sliding clamp accumulation in live Escherichia coli cells. Nat. Commun. *5*, 5820.

Neuman, K.C., Charvin, G., Bensimon, D., and Croquette, V. (2009). Mechanisms of chiral discrimination by topoisomerase IV. Proc. Natl. Acad. Sci. USA *106*, 6986–6991.

Nicolas, E., Upton, A.L., Uphoff, S., Henry, O., Badrinarayanan, A., and Sherratt, D. (2014). The SMC complex MukBEF recruits topoisomerase IV to the origin of replication region in live Escherichia coli. MBio *5*, e01001–e01013.

Nolivos, S., and Sherratt, D. (2014). The bacterial chromosome: architecture and action of bacterial SMC and SMC-like complexes. FEMS Microbiol. Rev. *38*, 380–392.

Persson, F., Lindén, M., Unoson, C., and Elf, J. (2013). Extracting intracellular diffusive states and transition rates from single-molecule tracking data. Nat. Methods *10*, 265–269.

Postow, L., Crisona, N.J., Peter, B.J., Hardy, C.D., and Cozzarelli, N.R. (2001). Topological challenges to DNA replication: conformations at the fork. Proc. Natl. Acad. Sci. USA *98*, 8219–8226.

Reyes-Lamothe, R., Sherratt, D.J., and Leake, M.C. (2010). Stoichiometry and architecture of active DNA replication machinery in *Escherichia coli*. Science *328*, 498–501.

Stone, M.D., Bryant, Z., Crisona, N.J., Smith, S.B., Vologodskii, A., Bustamante, C., and Cozzarelli, N.R. (2003). Chirality sensing by Escherichia coli topoisomerase IV and the mechanism of type II topoisomerases. Proc. Natl. Acad. Sci. USA *100*, 8654–8659.

Stracy, M., Uphoff, S., Garza de Leon, F., and Kapanidis, A.N. (2014). In vivo single-molecule imaging of bacterial DNA replication, transcription, and repair. FEBS Lett. *588*, 3585–3594.

Stracy, M., Lesterlin, C., Garza de Leon, F., Uphoff, S., Zawadzki, P., and Kapanidis, A.N. (2015). Live-cell superresolution microscopy reveals the organization of RNA polymerase in the bacterial nucleoid. Proc. Natl. Acad. Sci. USA *112*, E4390–E4399.

Uphoff, S., Reyes-Lamothe, R., Garza de Leon, F., Sherratt, D.J., and Kapanidis, A.N. (2013). Single-molecule DNA repair in live bacteria. Proc. Natl. Acad. Sci. USA *110*, 8063–8068.

Vos, S.M., Tretter, E.M., Schmidt, B.H., and Berger, J.M. (2011). All tangled up: how cells direct, manage and exploit topoisomerase function. Nat. Rev. Mol. Cell Biol. *12*, 827–841.

Vos, S.M., Lee, I., and Berger, J.M. (2013a). Distinct regions of the Escherichia coli ParC C-terminal domain are required for substrate discrimination by topoisomerase IV. J. Mol. Biol. *425*, 3029–3045.

Vos, S.M., Stewart, N.K., Oakley, M.G., and Berger, J.M. (2013b). Structural basis for the MukB-topoisomerase IV interaction and its functional implications in vivo. EMBO J. *32*, 2950–2962.

Wang, X., Reyes-Lamothe, R., and Sherratt, D.J. (2008). Modulation of Escherichia coli sister chromosome cohesion by topoisomerase IV. Genes Dev. *22*, 2426–2433.

Woo, J.S., Lim, J.H., Shin, H.C., Suh, M.K., Ku, B., Lee, K.H., Joo, K., Robinson, H., Lee, J., Park, S.Y., et al. (2009). Structural studies of a bacterial condensin complex reveal ATP-dependent disruption of intersubunit interactions. Cell *136*, 85–96.

Cell Reports

Supplemental Information

The Localization and Action of Topoisomerase IV in *Escherichia coli* Chromosome Segregation Is Coordinated by the SMC Complex, MukBEF

Pawel Zawadzki, Mathew Stracy, Katarzyna Ginda, Katarzyna Zawadzka, Christian Lesterlin, Achillefs N. Kapanidis, and David J. Sherratt

The Localization and Action of Topoisomerase IV in *Escherichia coli* Chromosome Segregation is Coordinated by the SMC Complex, MukBEF

Authors:

Pawel Zawadzki[1,4], Mathew Stracy[2,4], Katarzyna Ginda[1], Katarzyna Zawadzka[1], Christian Lesterlin[1,3], Achillefs N. Kapanidis[2], David J. Sherratt[1*]

Affiliations:

[1]Department of Biochemistry, University of Oxford, South Parks Road, Oxford, OX1 3QU, UK.

[2]Biological Physics Research Group, Clarendon Laboratory, Department of Physics, University of Oxford, Parks Road, Oxford, OX1 3PU, UK.

[3]Bases Moleculaires et Structurales des Systemes Infectieux, UMR 5086, Centre National de la Recherche Scientifique, University of Lyon, 69367 Lyon, France

[4]Co-first author

Corresponding author: D.J. Sherratt, Department of Biochemistry, University of Oxford, South Parks Rd, Oxford, OX1 3QU, UK Tel: +44 (0)1865 613237 Fax: +44 (0)1865 613238 david.sherratt@bioch.ox.ac.uk

Supplemental Experimental Procedures

1. Bacterial Strains and Cell Preparation.

All strains were derivatives of *Escherichia coli* K-12 AB1157 (Bachmann, 1972) and are listed in Table S1. The plasmids and oligonucleotides used are shown in Table S2. Fusion of genes with fluorescent tags used λRed recombination (Datsenko and Wanner, 2000). Fused genes were transferred to generate the final strains through P1 phage transduction (Thomason et al., 2007). For multiple insertions of modified genes, the Kan^r gene was removed using site-specific recombination through expression of the Flp recombinase from plasmid pCP20 (Datsenko and Wanner, 2000). *lacO* and *tetO* arrays were inserted 16 kb CCW of *oriC* (*ori1*) and 50 kb CW of *dif* (*ter3*), respectively (Badrinarayanan et al., 2012). Justifiably, concerns have been raised about the interpretation of results form experiments using fluorescent fusions because the fluorescent protein may interfere with function of the protein it is tagged to, and/or the fluorescent tag itself may influence the localization and apparent copy number of the protein under study (Landgraf et al., 2012; Wang et al., 2014). We are confident that our data and interpretations are physiologically relevant and are free from artefacts. For example, fusions of PAmCherry and mYPet to any of the components of TopoIV or MukBEF, or to DnaN in the case of mYPet, gave functional fusions when expressed from the endogenous chromosomal context in the absence of wild type protein. Fusions to a given protein showed similar cellular behavior independently of whether the imaging used PALM, widefield, or SIM. Furthermore, fusions to different proteins behaved in a protein-specific manner that reflected the known biology of the proteins. Growth rates, cell size distributions and flow cytometry profiles were identical to those of wild type cells (Table S3; Figure S1A). Finally, the cellular behavior of ParC-PAmCherry expressing cells changed predictably when its interaction with MukBEF clusters was impaired by deleting MukB or by over-expressing ParC-CTD or when norfloxacin was used to covalently link TopoIV to DNA.

Strains were streaked onto LB plates with appropriate antibiotics. Single colonies were inoculated into M9 glycerol (0.2%) and grown overnight at 37ºC to A_{600} 0.4-0.6, then diluted into fresh M9 and grown to A_{600} 0.1. Cells were centrifuged and immobilized on agarose pads between two glass coverslips (0.17mm thickness, heated to 500ºC for 1 h to remove any fluorescent background particles). We prepared 1% agarose pads by mixing low-fluorescence 2% agarose (Bio-Rad) in dH_2O 1:1 with 2x growth medium. For fixation, centrifuged cells prepared as above were resuspended into 2.5% paraformaldehyde in PBS buffer and fixed for 45 min shaking at 22ºC. Fixed cells were washed with PBS and immobilized on agarose pads as above. Nucleoids were visualized using 5 μg ml^{-1} 4′,6-diamidino-2-phenylindole (DAPI). Norfloxacin was used at 5 μg ml^{-1}. Cells were placed on an agarose pad containing the drug 5 minutes prior the experiment.

2. Epifluorescence Microscopy

Conventional wide-field fluorescence microscopy used an Eclipse TE2000-U microscope (Nikon), equipped with an 100x/NA1.4 oil PlanApo objective and a Cool-Snap HQ^2 CCD, and using Metamorph software for image acquisition.

Sister *ori1* cohesion time in the strain KG52 containing plasmid pZ68 (overproducing a ParC CTD domain) was assessed in a 5-min time-lapse analysis. We have measured the time from replisome appearance at initiation to *ori1* segregation. Chromosomal genetic loci were visualized using fluorescent repressor–operator systems. A *lacO* array of 240 copies was inserted 16 kb CCW of oriC (*ori1)*; LacI-mCherry was expressed from the chromosomal *leuB* locus, regulated by the *lac* promoter (Wang et al., 2008). A chromosomally encoded mYPet-DnaN fusion protein was used as a marker for the replisome (Moolman et al., 2014; Reyes-Lamothe et al., 2010). Cells were growing exponentially in minimal medium supplemented with glycerol, at 37°C (generation time ~100 min). CTD overproduction was induced by addition of L-Arabinose, final concentration 0.2%, 3 hours prior to the experiment. As a control, the strain with the empty plasmid, pBAD24 (Guzman et al., 1995) was used.

3. 3D Structured-Illumination Microscopy

Super-resolution 3D-SIM imaging was performed as in (Lesterlin et al., 2014), on a DeltaVision OMX V3 (Applied Precision/GE Healthcare) equipped with a Blaze SIM module, a ×60/1.42 oil UPlanSApo objective (Olympus), 405 nm and 488 nm diode

lasers and three sCMOS cameras (PCO). Cells were grown in LB in order to obtain more discrete lobed nucleoids. Three-dimensional two-color stacks of MukB-mYPet and DAPI stained DNA were obtained using sequential acquisition of 512 × 512 pixels image stacks with 8 x 125 nm z-sections (sample thickness 0.75 μm). Each z-section results from striped illumination patterns rotated to the three angles (−60°, 0°, +60°) and shifted in five phase steps. Acquisition settings were as follows: MukB–mYPet, 30 ms exposure with 488 nm laser (100 % transmission) and DAPI, 15 ms exposure with 405 nm laser (100% transmission). 3D-SIM raw data were computationally reconstructed with SoftWoRx 6.0 (Applied Precision) using a Wiener filter setting of 0.002 and channel specifically measured optical transfer functions to generate a super-resolution three-dimensional image stack. Images from the different channels were aligned using parameters obtained from calibration measurements with 0.2-μm-diameter TetraSpeck beads (Life Technologies) using the OMX Editor software (C. Weisiger and J. Sedat, unpublished). IMARIS analysis software (BITPLANE) was used to generate 3D rendering of fluorescent signals (Figure S3C; Movie 1), with bulk nucleoid DNA represented by a red wireframe, which excludes fluorescence values that are below the threshold of 20% of the maximum intensity value on the raw DAPI image. This results in exclusion of background fluorescence and the 'cloudy' fluorescence signal at the periphery of the nucleoid. To identify regions of highest DNA density (solid red) surfaces including regions with fluorescence values above the exclusion threshold of 70% of the maximum DAPI intensity values were generated. The distance between MukBEF clusters and the highest DNA density zone of the nucleoid was extracted from z-projections of the 3D-SIM stack to obtain 2D images. The *x* and *y* coordinates of the maximum intensity pixels for MukB and DAPI signals were identified using the FIJI "*find maxima*" function. The distance between the MukBEF maximum intensity pixel and the closest DAPI maximum intensity pixel was then calculated. Pixels were converted to distances using 0.082 μm/pixel. The mean distance between the brightest DAPI pixel and the brightest MukB-mYPet pixel (0.22 μm ± 0.12 μm) was derived from 97 distances, measured from four fields of view in two independent experiments.

4. PALM Microscopy

Live cell single-molecule-tracking PALM was performed on a custom-built total internal reflection fluorescence (TIRF) microscope built around the Rapid Automated

Modular Microscope (RAMM) System (ASI Imaging). Photoactivatable mCherry activation was controlled by a 405 nm laser and excitation with 561 nm. GFP and YFP excitation was provided by a 488 nm laser. All lasers provided by a multi-laser engine (iChrome MLE, Toptica). At the fiber output, the laser beams were collimated and focused (100x oil immersion objective, NA 1.4, Olympus) onto the sample under an angle allowing for highly inclined thin illumination (Tokunaga et al., 2008). Fluorescence emission was filtered by a dichroic mirror and notch filter (ZT405/488/561rpc & ZET405/488/561NF, Chroma). PAmCherry emission was projected onto an EMCCD camera (iXon Ultra, 512x512 pixels, Andor). The pixel size was 96 nm. Brightfield cell images were recorded with an LED source and condenser (ASI Imaging). Sample position and focus were controlled with a motorized piezo stage, a z-motor objective mount, and autofocus system (MS-2000, PZ-2000FT, CRISP, ASI Imaging).

5. Localization and Tracking.

PALM data for single-molecule-tracking analysis was localized using custom-written MATLAB software (MathWorks). Fluorophore images were identified for localization by band-pass filtering and applying an intensity threshold to each frame of a super-resolution movie. Candidate positions were used as initial guesses in a two-dimensional elliptical Gaussian fit for high-precision localization. Free fit parameters were x-position, y-position, x-width, y-width, elliptical rotation angle, intensity, background. Single-particle tracking analysis was performed by adapting the MATLAB implementation of the algorithm described in (Crocker and Grier, 1996). Positions were linked to a track if they appeared in consecutive frames within a window of 5 pixels (0.48 μm). In rare cases when multiple localizations fell within the tracking radius, tracks were linked such that the sum of step distances was minimized. We used a memory parameter of 1 frame to allow for transient (1 frame) disappearance of the fluorophore image within a track due to blinking or missed localization.

6. Molecule Counting

We counted the total number of ParC or ParE molecules by recording long movies (61000 frames) – until no further activation was observed. Copy numbers were derived from observed counts of molecules per cell. The experimentally determined

number of photoactivatable molecules likely underestimates the real copy number due to the fact that not all PAmCherry proteins can be activated (Durisic et al., 2014). In this study, using Xenopus oocytes, ~50% of PAmCherry molecules could be activated, with ~85% of the molecules not blinking. We do not have the appropriate correction factor for *E. coli*, but think it likely that we underestimate the copy number by a factor of ~2. All cells in the steady state population were analyzed, then the copy number was normalized to a 2.5μm long cell (the length soon after birth). Additionally, cells were placed on an agarose pad with norfloxacin (5μg/ml) in order to increase the fraction of immobile molecules. To estimate the number of TopoIV heterotetramers we used data from Figure 1C. There are 210 ParE molecules per cell; 32% are immobile and 24% are slowly diffusing. Therefore, 56% of 210 ParE molecules (117) are complexed with ParC, giving ~60 $ParC_2ParE_2$ TopoIV heterotetramers present at any time.

To estimate the number of TopoIV molecules associated with MukBEF clusters we used two calculations derived using different datasets. First, given that 14% of ~210 ParE molecules (~30) were dependent on MukB and immobile (and therefore in TopoIV heterotetramers), we deduced that ~15 TopoIV molecules were MukBEF cluster-associated at any time. Second, we used the 20% of 289 ParC molecules that were close to MukBEF clusters in the radial distribution analysis (Figure 2B; ~58 molecules). Since ~42% of all ParC molecules overall were in TopoIV heterotetramers (compare Figure 1B and 1C), we get a value of ~12 TopoIV molecules associated with MukBEF clusters. In contrast, if we compare Figure 2A top and middle panels, we get 16% of ~289 ParC molecules being immobile and dependent on MukB (~46). Since we deduce above that ~30 TopoIV heterotetramers are immobile and dependent on MukB, then we deduce that 65% of ParC molecules are in MukB-dependent TopoIV heterotetramers. 65% of the ~58 ParC molecules close to clusters gives ~19 TopoIV heterotetamers associated with MukBEF clusters. We conclude therefore that 12-19 TopoIV molecules were MukBEF-cluster associated at any time and in the main text, we use the value of ~15, which is the value from the first method.

7. Measuring the Diffusion of PAmCherry Labeled Proteins.

We determined the mobility of each molecule by calculating an apparent diffusion coefficient from the one-step mean-squared displacement (MSD) of the track using:

Equation 1

$$D^* = \frac{1}{4n\Delta t}\sum_{i=1}^{n}[x(i\Delta t) - x(i\Delta t + \Delta t)]^2 + [y(i\Delta t) - y(i\Delta t + \Delta t)]^2$$

Where $x(t)$ and $y(t)$ are the coordinates of the molecule at time t, the frame time of the camera is Δt, and n is the number of steps in the trajectory. Tracks shorter than $n = 4$ steps long were discarded for this analysis because the higher uncertainty in *D** value.

For a molecule with diffusion coefficient D, the probability distribution of obtaining a D* value, x , is given by:

Equation 2

$$f(x; D, n) = \frac{({}^{n}/_{D})^{n} x^{n-1} e^{-nx/D}}{(n-1)!}$$

Where n is the number of displacement steps in the trajectory. To allow for fitting to the D^* distribution longer tracks were truncated after 5th localization (i.e. $n = 4$), and the $n = 4$ equation was used:

Equation 3

$$f(x; D) = \frac{({}^{4}/_{D})^{4} x^{3} e^{-4x/D}}{6}$$

Least squares fitting to the histogram of *D** values was used. A single species model fits poorly to the data (Figure S1B). We reasoned that at least two species with different mobilities are present: mobile molecules diffusing and transiently binding DNA, and immboile molecules bound to DNA. We therefore introduced a second species:

Equation 4

$$f(x; D_1, D_2, A_1, A_2) = \frac{A_1({}^{4}/_{D_1})^{4} x^{3} e^{-4x/D_1}}{6} + \frac{A_2({}^{4}/_{D_2})^{4} x^{3} e^{-4x/D_2}}{6}$$

Where D_1 and D_2 are the diffusion coefficients of the two different species, and $A_1$and A_2 are the fraction of molecules found in each state, and $A_1 + A_2 = 1$. For fitting to ParE a third species was added in a similar fashion.

We note that Equations 1-3 assume that the *n* displacements averaged to generate the *D** value are independent, which is not strictly true as the same localisation is used to determine the preceeding and following displacement step, and hence any localisation error and motion blurring of this localisation will effect both displacements (Michalet, X. 2010). To verify that Equations 1-3 are adequate approximations for *D** values generated from overlapping displacements we used ParC trajectories with 7 displacements and determined the *D** value from either the first 4 sequential steps (using Equation 1, with *n* = 4) or from nonoverlapping steps using:

Equation 5

$$D^* = \frac{1}{16\Delta t}\sum_{i=1}^{4}[x((2i-1)\Delta t) - x(2i\Delta t)]^2 + [y((2i-1)\Delta t) - y(2i\Delta t)]^2$$

The *D** distribution from sequential steps was essentially identical to the distribution generated with nonoverlapping steps and fitting both with Equation 4 gave identical values (see Fig. S1 J).

To establish the apparent diffusion of the DNA-bound species, we then turned to a well charaterized control protein DNA polymerase 1 (Pol1) which shows clearly distinct *D** populations for molecules specifically bound to DNA and those mobile molecules diffusing through the nucleoid searching the for substrate (Fig. S1C). Inducing DNA methylation damage by incubating cells with MMS increases the fraction of specifically bound molecules (described in (Uphoff et al., 2013)), making it easier to resolve the distribution of bound molecules. Fitting this distribution to two diffusing species allows us to determine the *D* value of specifically bound molecules, as *D** = 0.11 $\mu m^2 s^{-1}$. This apparent motion of bound molecules is mainly due to localisation error, which manifests as a shift to the right in *D** value of σ_{loc}^2 /Δt, hence immobile molecules appear to have a *D** value of ~ 0.1 $\mu m^2 s^{-1}$. Using this D* value

for bound molecules to constrain one D^* species and allowing a second unconstrained D^* species fits well to the data giving two populations of immobile molecules, D_{imm} = 0.11 μm^{2}s^{-1}, and molecules slowly diffusing, D_{slow} = 0.35 μm^{2}s^{-1} (Fig.1B).

ParE D^* histograms, on the other hand, fits poorly to a two species distribution. Instead, we reasoned that third population with faster diffusion exists, representing uncomplexed ParE molecules which do not bind DNA. Consistent with this overexpression of unlabelled ParE resulted in the dramatic increase in fraction of this third population showing that this population represents individual ParE subunits not nomplexed with ParC (Figure S2G and H). Fiting three diffusing species to overexpression data with two population constrained at values obtained for ParC; D_{imm} =0.11μm^{2}s^{-1}, and D_{slow} =0.35 μm^{2}s^{-1}, resulted in a good fit to the data, with a third population at D_{fast} =0.94 μm^{2}s^{-1} (Figure S1H). Subsequently, we fit ParE distribution with three species and constrained all three D values at; D_{imm} =0.11 μm^{2}s^{-1}, D_{slow} =0.35 μm^{2}s^{-1} and D_{fast} =0.94 μm^{2}s^{-1}.

8. Intracellular Spatial Distributions of Classified Molecules

A two species fit to the ParC gave 36 % as immobile and 64 % mobile. We determined a D^* threshold (0.16 μm^{2}s^{-1}) which preserves the ratio of immobile-to-mobile molecules established from fitting to the population to sort each individual ParC track as immobile or mobile based on its mobility (Stracy et al., 2015; Uphoff et al., 2013). In order to minimize the uncertainty when determining the D^* for categorizing individual molecules, only trajectories with at least 4 steps were used, and all steps longer than 4 were included. Based on the overlap between the two fitted distributions truncated at 4 steps, we estimate that this threshold correctly categorized 86 % of molecules. This is likely to be an underestimate of the true categorization accuracy as the variance in the distributions of longer trajectories is smaller thus the overlap is reduced.

Using this threshold we determined the average spatial distribution of immobile and mobile ParC molecules over many cells. To do this, cells were segmented from brightfield images using MicrobeTracker (Sliusarenko et al., 2011). The probability distributions along the cell short-axis were calculated by determining the distance of each localization from the cell midline, with the distances normalized

to 1 and -1 at the cell membrane and 0 at the cell midline. The analytical probability distribution for a uniform distribution within a cylindrical volume is also presented (Sanamrad et al., 2014) is given by:

$$f(\rho) = \frac{2\sqrt{\max(1-\rho^2, 0)}}{\pi}$$

Where ρ is the relative short-axis position. This is the expected distribution from a molecule able to move freely through the complete cell volume, and is shown in Figure S1G for comparison with experimental distributions.

9. Clustering Analysis and Radial Distribution Analysis

The radial distribution functions and clustering analysis were performed in Matlab (Mathworks). The radial distribution function, g(*r*), was calculated for all ParC/E localizations inside segmented cells. First, the pairwise distances of all localizations were calculated on a cell by cell basis. Because of the small size of *E. coli*, as the radius, *r*, of the pairwise distance increases much of the area falls outside the cell. To account for this effect we simulated the randomly distributed points within the same segmented cell outline. For each segmented cell the same number of localizations was simulated as observed experimentally, and their pairwise distances calculated. This was repeated over all cells and the histogram of pairwise distances was divided by the simulated random pairwise distances to give the empirical radial distribution function, g(r), with random distribution having a g(*r*) of one.

A nearest neighbor clustering algorithm was implemented to cluster ParC localizations on a cell by cell basis inside cells. Localizations were counted as a cluster if more than 25 localizations clusters using a nearest neighbor threshold of 200 nm. The number of clusters per cell was recorded. Cells with fewer than 300 localizations were not used for this analysis.

10. Measuring Long-lasting Binding Events

PALM movies to measure long duration binding events were recorded at low continuous 561-nm excitation intensities using long exposure times (500 ms/frame, 750 ms/frame, and 1000 ms/frame) (Uphoff et al., 2013). At these exposure times mobile ParC-PAmCherry molecules are motion blurred over a large fraction of the

cell, whereas immobile ParC-PAmCherry molecules still appear as point sources, producing a diffraction limited spot. Elliptical Gaussian fitting was used as described in section S5. Bound and mobile molecules were distinguished by the width of the elliptical fits, with thresholds short axis-width < 160 nm and long axis-width < 200 nm to identify bound molecules (Figure S4E). The probability of observing a particular on-time is the product of the underlying binding-time probability and the bleaching probability. The bleaching-time distributions were measured independently using MukB-PAmCherry, which binds DNA in 1-3 large clusters per cell with a dwell time of ~50s (Badrinarayanan et al., 2012), with the same acquisition and excitation conditions. On-time and bleaching-time distributions were fitted with single-exponential functions to extract exponential-time constants t_{on} and t_{bleach}, and the binding-time constant was calculated by $t_{bound} = t_{on} \cdot t_{bleach} / (t_{bleach} - t_{on})$. Stochastic photoactivation of ParC-PAmCherry molecules before or during binding events does not influence our measurement, because the observed binding times follow an exponential distribution and are therefore memoryless. All three time regimes (500, 750 and 1000ms) gave similar binding times.

This analysis showed that most binding events are not long (>30 s) processive events. Nevertheless, the calculated binding time of 1.8 s ± 0.4 s assumes a single binding dwell time, so we cannot rule out a mixed population of catalytic events, with a majority of short (>1 s) and a minority of long-lived (>30 s) binding events. We therefore undertook a time-lapse experiment to increase fluorophore lifetime, by using 4 s delays between 1 s exposures. This analysis gave a binding time of 2.6 s ± 0.7 s (Figure S4F) indicating that there may be some longer binding events.

To estimate an upper limit for the fraction of longer events, which we propose would represent processive events lasting >30 s, based on analysis *in vitro* (Crisona et al., 2000; Stone et al., 2003), we undertook simulations that assumed a lower limit of 1 s for short (distributive) and 30 s for long (processive) events. 100,000 exponentially distributed dwell times were simulated with either 1s or 30 s dwells. The dwell times were truncated by the exponentially distributed bleaching times from an experimental MukB control at either 1 s exposures, or for 1 s + 4 s time-lapse. Corrected binding times were calculated for both conditions with varying fractions of 30 s events between 0% and 100 % to establish which fraction best matched the 1.8 s and 2.6 s binding times established from the experimental data. The closest match

was 22% long events. Because 1 s binding time is likely to be a lower limit for the distributive events, this indicates that ~22% is the upper limit for the fraction of long, and therefore presumably processive, catalytic events.

11. Determining the Diffusion Coefficients of Free and Immobile TopoIV

The free diffusion coefficient for TopoIV heterotetramers, D_{free}, was estimated by first establishing the diffusion coefficient for free ParE. The ParE subunit cannot bind DNA (Lee et al., 2013). Using the ParE-PAmCherry with overexpressed unlabeled ParE (which outcompetes ParE-PAmCherry in TopoIV molecules, Figure S1F) gave a clear distribution of molecules with much higher mobility, D_{fast} = 0.94 $\mu m^2 s^{-1}$. We verified that these mobile ParE subunits are not interacting with DNA and therefore occupy the full volume of the cell cytoplasm by plotting their probability density of molecules across the short axis of the cell (Figure S1G), which matched very well to the distribution expected from uniformly distributed molecules within a cylindrical volume of the same dimensions as a bacterial cell (Sanamrad et al., 2014).

The *D* value of mobile ParE therefore reports on the free diffusion coefficient. However, the apparent diffusion determined experimentally through particle tracking does not take into account confinement due to the small size of bacteria, and other effects such as localization error and motion blurring (Stracy et al., 2014; Uphoff et al., 2013; Uphoff et al., 2014). To determine the accurate D_{free} value we simulated Brownian motion confined within a volume corresponding to the average size of cells imaged in experiments, defined as a cylindrical volume of length 2 µm long and 0.9 µm wide with hemispherical endcaps with a radius of 0.9 µm (Uphoff et al., 2013). Each 15 ms frame was split into 100 sub-frames with Gaussian distributed displacements in each sub-frame. Each molecule trajectory was given a random starting time to mimic stochastic photoactivation. The trajectory was then simulated until photobleaching with duration sampled from an exponential distribution with a mean time equal to our experimentally determined photobleaching lifetime (~70 ms). The sub-frame distributions were then averaged to give a position for each frame, and a localization error sampled from a Guassian distribution with σ_{loc} = 40 nm (determined from experiments in fixed cells) was added. The list of simulated localizations, with their corresponding frame number was then analyzed using the same tracking algorithm with the same settings as used for the experimental data.

The outputted tracks could then be analyzed in exactly the same way as experimental data.

Running simulations with different D values between 0 and 5 μm^2s^{-1}, we found that the D_{fast} = 0.94 μm^2s^{-1} (Figure S4B; green line) for ParE was best matched by a simulated D_{free} = 2.4 μm^2s^{-1}. ParE and ParC subunits are similar in size (ParC = 84kDa, ParE = 70kDa). The TopoIV heterotetramer contains 2 ParE and 2 ParC molecules. Given this, we estimated that the TopoIV heterotetramer had ~4 x the volume of ParE, and hence a radius ~1.59 times as large. Because the diffusion coefficient for a spherical particle depends linearly on the radius, we therefore estimate that the D_{free} for TopoIV is 2.4/1.59 = 1.51 μm^2s^{-1}.

The movement of DNA loci is small relative to the movement of diffusing TopoIV molecules (Elmore et al., 2005). The majority of the experimentally observed D = 0.11 μm^2s^{-1} value for immobile molecules (bound to DNA) was due to apparent motion from the localization error (determined from fixed cells). A very small D motion of 0.06 μm^2s^{-1} on top of the localization error best matched the observed D = 0.11 μm^2s^{-1} in live cells (Figure S4B; red line).

12. Simulating Molecules Interconverting between Diffusive States.

We hypothesized that our observed slow diffusion, D_{slow} = 0.35 μm^2s^{-1} for mobile ParC molecules was due to ParC molecules interconverting between the D_{free} and D_{imm} states. Simulations showed that observed apparent D_{slow} = 0.35 μm^2s^{-1} corresponds to accurate D = 0.4 $\mu m^2s^{-1,}$ which is significantly lower than D = 1.51 μm^2s^{-1}, estimated from the size of TopoIV. Having established an estimate of these values we determined the fraction of these molecules spent in each state f_{free} and f_{imm} using:

$$D_{slow} = (1- f_{free})*D_{imm} + f_{free}*D_{free}$$

Solving these equation gave f_{free} = 0.27 and f_{imm} = 0.73. To establish if we could recapitulate our experimental data with these fractions, we simulated molecules, which could rapidly interconvert the two states; D_{free} and D_{bound}, while spending 27% of time freely diffusing and 73% as bound. The duration of this transient binding, t_{imm}, was randomly sampled from an exponential with a mean of 1ms. The simulated

interconverting species (Figure.S4C) shows that the experimentally observed *D* can be recapitulated using two states; D_{free} and D_{bound}. As t_{imm} was (1ms being an upper limit) << observation time, the interconverting species appeared as a single diffusing species with reduced mobility, and could be fitted with an analytical equations for a single diffusing species at $D = 0.35\ \mu m^2 s^{-1}$.

In time lapse experiments with a 15 ms exposures followed by a 35 ms delay, a reduction in the fraction of molecules that remained immobile over the duration of the track was observed which was not seen in Δ*mukB* cells. Using 15ms exposures in Muk+ cells, the fraction of bound TopoIV is significantly higher than in Δ*mukB* cells (36%). However, in time-lapse experiments this bound fraction was reduced to the same level as Δ*mukB* cells (14%), indicating that there is a population of Muk-dependent TopoIV molecules which appear immobile over 15ms exposures, but appear mobile in time-lapse experiments. To determine the duration of the MukB dependent events we simulated molecules interconverting between D_{imm} and D_{free} and varied the mean duration of the binding events. Molecule trajectories were simulated and averaged to generate localizations at either 15 ms intervals or 15 ms intervals with 35ms delays to match normal and time-lapse experiments. The simulated localizations were analyzed with the same tracking and categorizing algorithm as used for experimental data. Plotting the reduction in the fraction of molecules categorized as bound in time-lapse simulations compared to normal simulations showed that binding for 30–70 ms recapitulated experimentally observed decrease (Figure S4D). For binding times significantly smaller than the exposure time of each frame the normal and time-lapse simulations showed little difference. In Δ*mukB* cells no decrease was observed between time-lapse and normal experiments. However, the observed *D* in Δ*mukB* cells was again = $0.35\ \mu m^2 s^{-1}$, suggesting that transient interactions with DNA, estimated to last ~1ms, are MukB-independent.

13. Flow Cytometry

Cells were grown in M9-gly to exponential phase ($A_{600} \approx 0.2$). 200 μl of the cultures were fixed with 3.5 ml of ice-cold 74% ethanol. For the staining procedure, the cells were pelleted and washed twice in 100 μl of cold staining buffer (10 mM Tris pH 7.4, 10 mM $MgCl_2$). The 100 μl samples were mixed with an equal volume of the staining

solution Syto-16 (3 μM). 100,000 events were recorded in a Becton Dickinson FACScalibur machine using FL1-H and results were analysed using FlowJo.

14. Determination of *in vivo* Dissociation Constants

At equilibrium, the dissociation constant between two proteins is expressed as:

$$K_D = \frac{C^a \times C^b}{C^{ab}}$$

Where C^a represents the concentration of protein A, C^b represents the concentration of protein B, and C^{ab} represents the concentration of the complex (Sudhaharan et al., 2009). Since we counted the average number of molecules inside 2.5 μm long cells, and determined the volume of a 2.5 μm long and 800nm wide *E.coli* cell to be ~ 1.1 μm^3 (Kubitschek and Friske, 1986)(Kubitschek and Friske, 1986) we assume 1 molecule/cell corresponds to a concentration of 1nM. Therefore, the concentrations in living cells, of ParC, ParE, and MukB are 289nM, 210nM, and 195nM respectively. To calculate the dissociation constant of TopoIV heterotetramers we assumed that a dimer of ParC will form a heterotetramer with a dimer of ParE.

15. Determination of Search Time Preceding Catalysis.

Since we measured the fraction of catalytically active TopoIV at any given time, $F_{catalysis}$, to be 14% (Figure 3B), and we measured the average dwell of the catalytic cycle, $t_{catalysis}$, to be 1.8 s (Figure 3D) we can calculate the average time for a given TopoIV molecule to locate and bind to its substrate, t_{search}, using $F_{catalysis} = t_{catalysis}/(t_{catalysis} + t_{search})$ (Uphoff et al., 2013). Using our measured values of $F_{catalysis}$ and $t_{catalysis}$ to solve this equation gives a search time of 11s. The combined catalytic dwell time and search time can be used to determine the reaction rate per molecule: $1/(t_{catalysis} + t_{search})$. Using this we found that a single TopoIV has a reaction rates of ~ 4.7 min^{-1}. This high rate of nearly five reactions per minute per molecule explains our observations that the fraction of catalytic events captured by norfloxacin treatment reaches saturation in less than a few minutes after addition of the antibiotic.

16. Determining ParC/E *D** Distributions at Different Points in the Cell Cycle

Cells were segmented from brightfield images using MicrobeTracker, giving a cell outline and a cell midline (Sliusarenko et al., 2011) and the positions of molecule

trajectories were determined relative to the cell midline, with the x-axis defined as the cell short axis and the y-axis defined as the cell long axis. Cells were binned by cell length (which is a reasonable proxy for their stage in the cell cycle), into three categories; short cells (2-3 μm long) having a single centrally located nucleoid, intermediate length cells (3-4 μm long), and longer cells (4-5 μm long) having replicated most of their chromosome which separate into distinct nucleoids prior to cell division (Figure S5F). Localizations within segmented cell boundaries were tracked and *D** values determined. Fitting the distribution of *D** values for ParC/E in small, intermediate, and long cells showed a similar proportion of heterotetramers in cells at these three different stages of the cell cycle (Figure S5G and H).

References

Bachmann, B.J. (1972). Pedigrees of some mutant strains of Escherichia coli K-12. Bacteriological reviews *36*, 525-557.

Badrinarayanan, A., Reyes-Lamothe, R., Uphoff, S., Leake, M.C., and Sherratt, D.J. (2012). In vivo architecture and action of bacterial structural maintenance of chromosome proteins. Science *338*, 528-531.

Crisona, N.J., Strick, T.R., Bensimon, D., Croquette, V., and Cozzarelli, N.R. (2000). Preferential relaxation of positively supercoiled DNA by E. coli topoisomerase IV in single-molecule and ensemble measurements. Genes & development *14*, 2881-2892.

Crocker, J.C., and Grier, D.G. (1996). When Like Charges Attract: The Effects of Geometrical Confinement on Long-Range Colloidal Interactions. Physical review letters *77*, 1897-1900.

Datsenko, K.A., and Wanner, B.L. (2000). One-step inactivation of chromosomal genes in Escherichia coli K-12 using PCR products. Proceedings of the National Academy of Sciences of the United States of America *97*, 6640-6645.

Durisic, N., Laparra-Cuervo, L., Sandoval-Alvarez, A., Borbely, J.S., and Lakadamyali, M. (2014). Single-molecule evaluation of fluorescent protein photoactivation efficiency using an in vivo nanotemplate. Nature methods *11*, 156-162.

Elmore, S., Muller, M., Vischer, N., Odijk, T., and Woldringh, C.L. (2005). Single-particle tracking of oriC-GFP fluorescent spots during chromosome segregation in Escherichia coli. Journal of structural biology *151*, 275-287.

Guzman, L.M., Belin, D., Carson, M.J., and Beckwith, J. (1995). Tight regulation, modulation, and high-level expression by vectors containing the arabinose PBAD promoter. Journal of bacteriology *177*, 4121-4130.

Kubitschek, H.E., and Friske, J.A. (1986). Determination of bacterial cell volume with the Coulter Counter. Journal of bacteriology *168*, 1466-1467.

Landgraf, D., Okumus, B., Chien, P., Baker, T.A., and Paulsson, J. (2012). Segregation of molecules at cell division reveals native protein localization. Nature methods *9*, 480-482.

Lee, I., Dong, K.C., and Berger, J.M. (2013). The role of DNA bending in type IIA topoisomerase function. Nucleic acids research *41*, 5444-5456.

Lesterlin, C., Ball, G., Schermelleh, L., and Sherratt, D.J. (2014). RecA bundles mediate homology pairing between distant sisters during DNA break repair. Nature *506*, 249-253.

Moolman, M.C., Krishnan, S.T., Kerssemakers, J.W., van den Berg, A., Tulinski, P., Depken, M., Reyes-Lamothe, R., Sherratt, D.J., and Dekker, N.H. (2014). Slow unloading leads to DNA-bound beta2-sliding clamp accumulation in live Escherichia coli cells. Nature communications *5*, 5820.

Reyes-Lamothe, R., Sherratt, D.J., and Leake, M.C. (2010). Stoichiometry and architecture of active DNA replication machinery in Escherichia coli. Science *328*, 498-501.

Sanamrad, A., Persson, F., Lundius, E.G., Fange, D., Gynna, A.H., and Elf, J. (2014). Single-particle tracking reveals that free ribosomal subunits are not excluded from the Escherichia coli nucleoid. Proceedings of the National Academy of Sciences of the United States of America *111*, 11413-11418.

Sliusarenko, O., Heinritz, J., Emonet, T., and Jacobs-Wagner, C. (2011). High-throughput, subpixel precision analysis of bacterial morphogenesis and intracellular spatio-temporal dynamics. Molecular microbiology *80*, 612-627.

Stone, M.D., Bryant, Z., Crisona, N.J., Smith, S.B., Vologodskii, A., Bustamante, C., and Cozzarelli, N.R. (2003). Chirality sensing by Escherichia coli topoisomerase IV and the mechanism of type II topoisomerases. Proceedings of the National Academy of Sciences of the United States of America *100*, 8654-8659.

Stracy, M., Lesterlin, C., Garza de Leon, F., Uphoff, S., Zawadzki, P., and Kapanidis, A.N. (2015). Live-cell superresolution microscopy reveals the organization of RNA polymerase in the bacterial nucleoid. Proceedings of the National Academy of Sciences of the United States of America.

Stracy, M., Uphoff, S., Garza de Leon, F., and Kapanidis, A.N. (2014). In vivo single-molecule imaging of bacterial DNA replication, transcription, and repair. FEBS letters *588*, 3585-3594.

Sudhaharan, T., Liu, P., Foo, Y.H., Bu, W., Lim, K.B., Wohland, T., and Ahmed, S. (2009). Determination of in vivo dissociation constant, KD, of Cdc42-effector complexes in live mammalian cells using single wavelength fluorescence cross-correlation spectroscopy. The Journal of biological chemistry *284*, 13602-13609.

Thomason, L.C., Costantino, N., and Court, D.L. (2007). E. coli genome manipulation by P1 transduction. Current protocols in molecular biology / edited by Frederick M. Ausubel ... [et al.] *Chapter 1*, Unit 1 17.

Tokunaga, M., Imamoto, N., and Sakata-Sogawa, K. (2008). Highly inclined thin illumination enables clear single-molecule imaging in cells. Nature methods *5*, 159-161.

Uphoff, S., Reyes-Lamothe, R., Garza de Leon, F., Sherratt, D.J., and Kapanidis, A.N. (2013). Single-molecule DNA repair in live bacteria. Proceedings of the National Academy of Sciences of the United States of America *110*, 8063-8068.

Uphoff, S., Sherratt, D.J., and Kapanidis, A.N. (2014). Visualizing protein-DNA interactions in live bacterial cells using photoactivated single-molecule tracking. Journal of visualized experiments : JoVE.

Wang, S., Moffitt, J.R., Dempsey, G.T., Xie, X.S., and Zhuang, X. (2014). Characterization and development of photoactivatable fluorescent proteins for single-molecule-based superresolution imaging. Proceedings of the National Academy of Sciences of the United States of America *111*, 8452-8457.

Wang, X., Reyes-Lamothe, R., and Sherratt, D.J. (2008). Modulation of Escherichia coli sister chromosome cohesion by topoisomerase IV. Genes & development *22*, 2426-2433.

Supplementary Figures

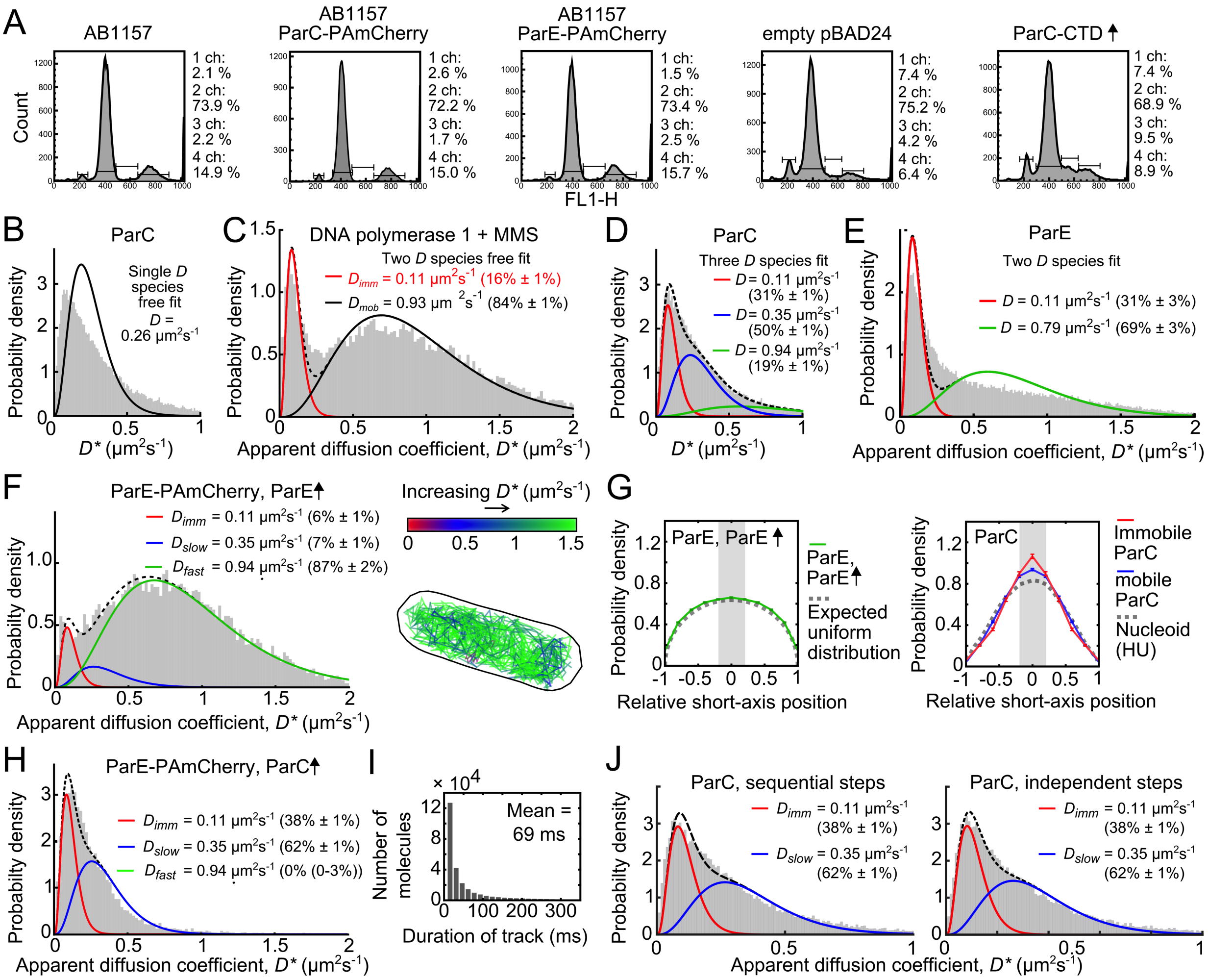

Figure S1. PALM Tracking of ParC/E Molecules in Live *E. coli.* Related to Figure 1. A) Flow cytometry profiles of cells with PAmCherry fusions (derivatives of AB1157) and after ParC-CTD over-expression in strain KK52. Ranges indicate cells with (from the left); one, two, three and four chromosomes. B) The distribution of apparent diffusion coefficients (D^*) of 73020 ParC molecules, fitted with a single species model. C) The distribution of apparent diffusion coefficients, D^*, for DNA polymerase1- PAmCherry grown in minimal media supplemented with MMS (100 mM). The distribution was fitted with a two species free fit, giving a population of specifically bound molecules with D_{imm} = 0.11 μm^{2}s^{-1}. D) The distribution of apparent diffusion coefficients (D^*) of 73020 ParC molecules, fitted with a three species model. Immobile population was constrained at value obtained for immobile molecules. We could not find any biological justification to fit three species model to ParC distribution. The resulting population of D_{slow} = 0.30 μm^{2}s^{-1} and D_{fast} = 0.65 μm^{2}s^{-1} could not be seen in any of the control overexpression experiments. Therefore, a three species fit does not describe true populations of ParC. E) The distribution of

apparent diffusion coefficients (D*) of 64551 ParE molecules fitted with two species model. Immobile population was constrained. The ParE data do not fit well to two species model well. F) The distribution of *D** values of 7101 ParE-PAmCherry molecules in a strain overexpressing unlabeled ParE (left). The distribution was fitted to a three species model with two species constrained at D_{imm} = 0.11 μm^{2}s^{-1} and D_{slow} = 0.35 μm^{2}s^{-1}. The third unconstrained fit shows the majority of molecules are fast moving, with D_{fast} = 0.94 μm^{2}s^{-1}. An example cell with ParE trajectories colored according to their *D** value (right). G) The probability density of ParE molecules across the cell short-axis in a strain overexpressing unlabeled ParE (left; 377 cells). 25% of molecules were located in the central region of the short axis (defined as the central 20% shown highlighted in grey). Dashed grey line shows the expected distribution for molecules uniformly distributed throughout a cylindrical cell volume. 25% of uniformly distributed molecules were expected to be located in the central region, agreeing well with the data. The short-axis distribution of immobile and mobile ParC molecules from Figure 3A is reproduced here for comparison (right). 39 % of immobile ParC molecules and 34% of mobile ParC molecules were located in the central region of the short-axis. The distribution of nucleoid associated protein HU (dashed line) shows the average spatial distribution of the nucleoid. H) The distribution of *D** values of 9508 ParE-PAmCherry molecules in a strain overexpressing unlabeled ParC. The distribution was fitted to a three species model with D values constrained at D_{imm} = 0.11 μm^{2}s^{-1}, D_{slow} = 0.35 μm^{2}s^{-1} and D_{fast} = 0.94 μm^{2}s^{-1}. After ParC overexpression no fast moving molecules were detected. I) Track length distribution for ParC molecules imaged at 15 ms exposure time. J) The *D** distribution from sequential steps was essentially identical to the distribution generated with non-overlapping steps and fitting both with Equation 4 gave identical values. Fitting ranges give 95% confidence intervals.

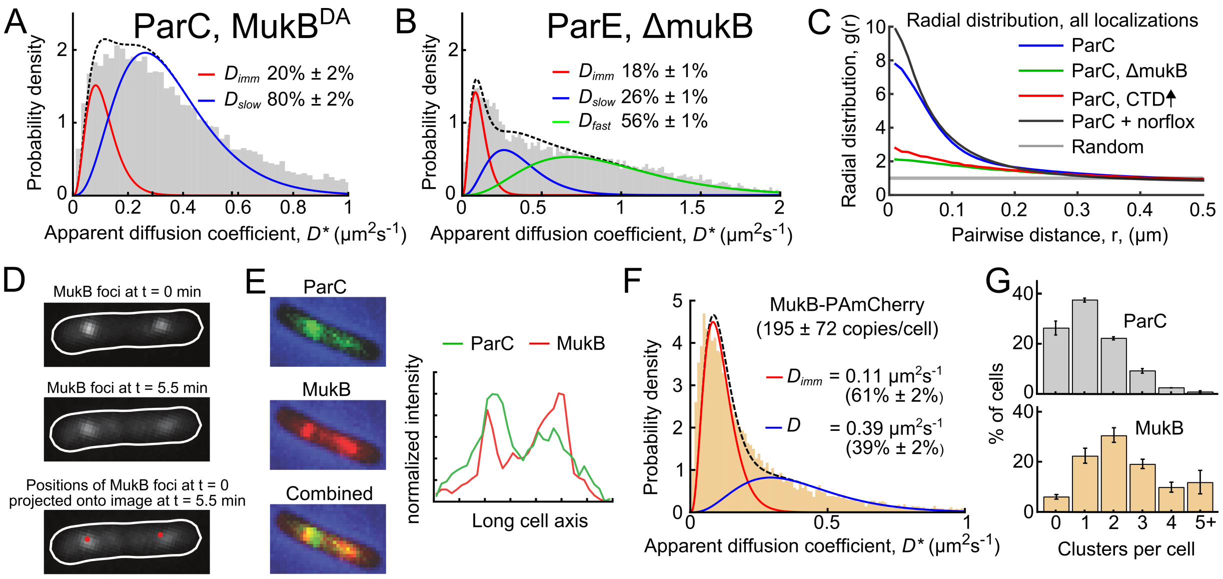

Figure S2. MukB Influences the Diffusion and Organization of TopoIV. Related to Figure 2. A) The distribution of D^* values for 13188 ParC molecules in *mukB*^{DA} cells. Distributions of D* were fitted with two species model with both D values constrained. B) The distribution of D^* values for 15893 ParE molecules in Δ*mukB* cells. Distributions of D* were fitted with three species model with all D values constrained. C) Radial distribution function calculated for all ParC localizations in WT conditions, with ParC-CTD overexpression, MukB deletion, and norfloxacin incubation. This analysis provides an unbiased assessment of cluster formation. D) Location of MukB foci before and after ~5 min PALM acquisition in example cell. E) Example of a cell imaged in epifluorescence with labeled MukB-mCherry foci (red) and ParC-YPet foci (green). The long-axis lines-scan of the fluorescence intensity is shown on the right. Frequently we observed only some of MukB foci were associated with distinct ParC clusters. F) Distribution of D^* values for 9068 MukB-PAmCherry trajectories. The distribution was fitted to a two species model with D_{imm} constrained at 0.11 μm^{2}s^{-1} and a second unconstrained fit. G) The number of clusters per cell formed by MukB defined by the same clustering parameters used for ParC. For comparison the number of ParC clusters per cell (Fig. 2A) is also shown here. MukB forms ~ twice as many clusters as ParC. Error bars indicate standard deviation of three experimental repeats. Fitting ranges give 95% confidence intervals.

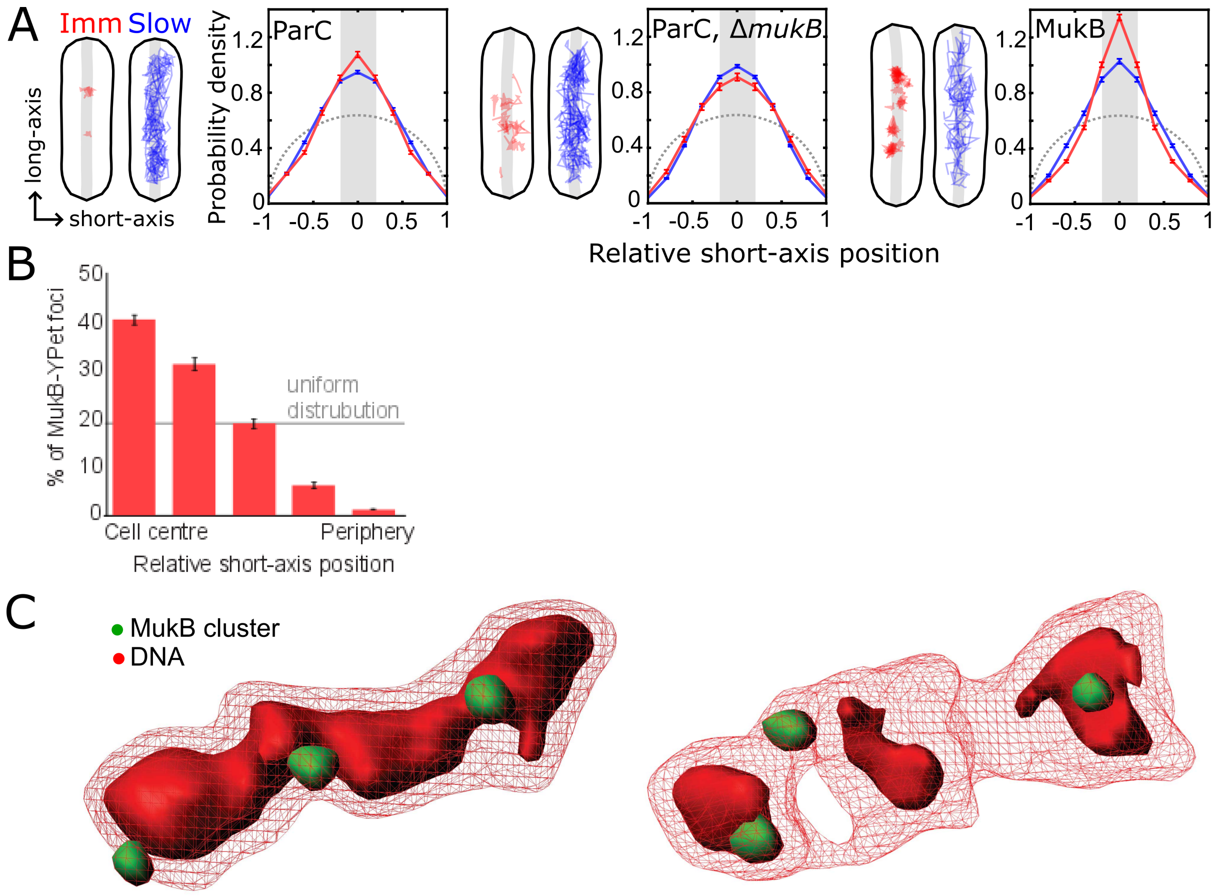

Figure S3. Intracellular Organization of MukB and TopoIV. Related to Figure 2. A) Example cells show intracellular location of ParC molecules sorted by their D* value as immobile (red) and slow (blue). Probability distribution of immobile and slow moving ParC molecules across the short cell axis in wild type (2386 cells) and DmukB (253 cells). Also shown are immobile and slow MukB molecules in wild-type cells (852 cells). Cell width is normalized to 0 at the cell midline and 1 and -1 at the cell membrane. Dotted lines indicate random distribution in a cellular cylinder and grey bars indicate central 20% of cell. Error bars show the square root of the number of observations per histogram bin assuming Poissonian statistics. B) Distributions of MukBEF foci across the short cell axis, in epifluorescence microscopy (1407 cells). Segments of the lateral positions were normalized to cell volume. For a uniform distribution, an equal fraction of foci in each segment is expected. Error bars indicate standard deviation of three experimental repeats. C) 3D-SIM surface rendering of two cells with MukB-mYPet and DAPI stained DNA. Green spots; MukBEF foci. Wireframe shows nucleoid DNA (threshold: excludes signal ≤20% of the maximum

DAPI intensity); red surface shows the DNA regions with the highest density (threshold: ≥ 70% of the maximum DAPI intensity). See Movie 1.

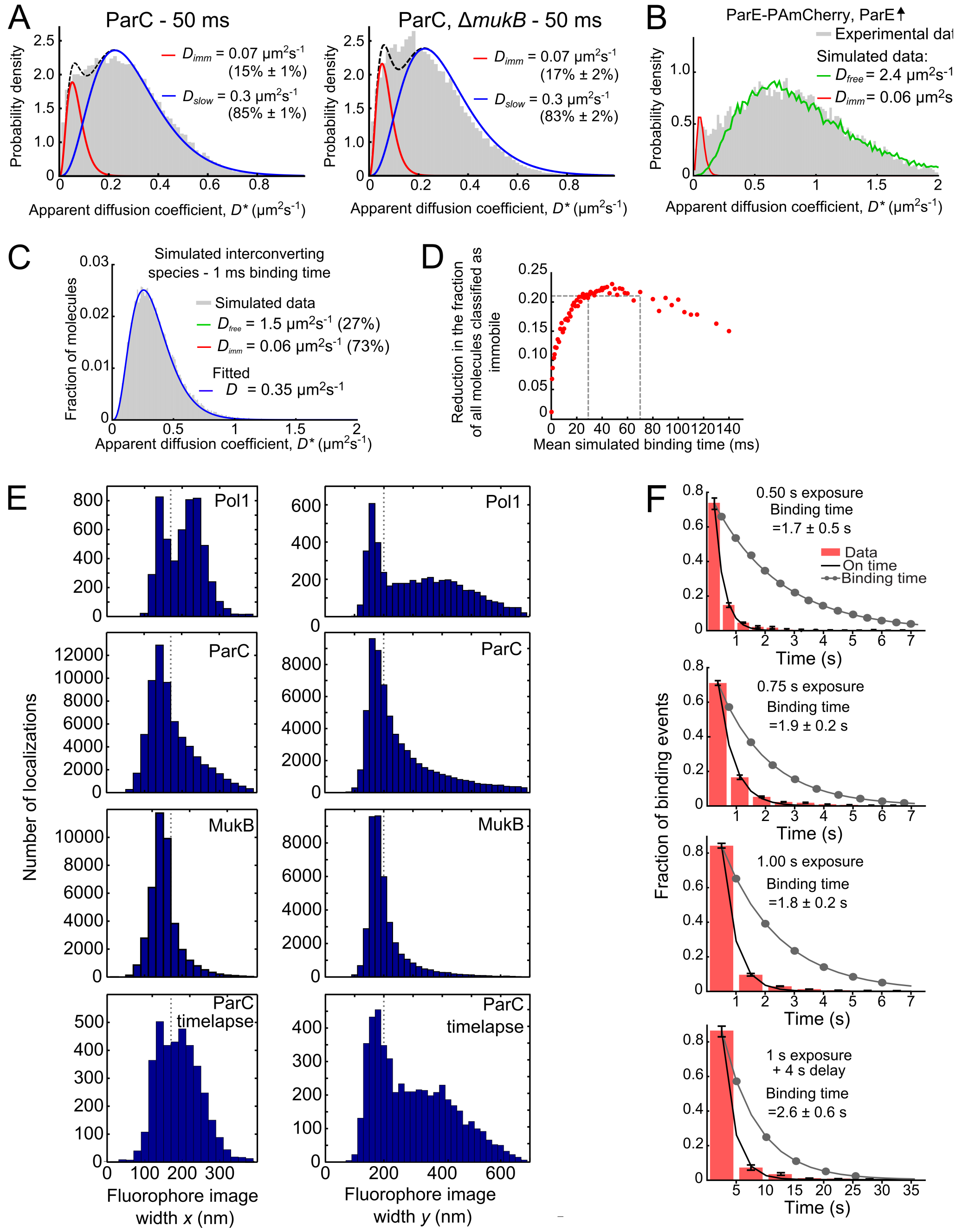

Figure S4. Two Populations of Immobile ParC. Related to Figure 3.

A) Distribution of D* values for 28188 ParC molecules imaged with a time-lapse using a 15 ms exposure followed by a 35 ms delay (left). Distribution of D* values for 26636 ParC molecules in ΔmukB cells imaged with a time-lapse (right). B) Comparison between experimental data for ParE-PAmCherry molecules in a strain

overexpressing unlabeled ParE and the distributions of two simulated diffusing species. The fast moving population agrees well with simulated molecules with Dfree = 2.4 μm2s-1. The immobile population agrees well with simulated molecules with Dimm = 0.06 μm2s-1. C) Simulated molecules interconverting between Dimm and Dfree = 1.5 μm2s-1 (corrected for the relative size of TopoIV compared to ParE). The binding time in the immobile state is set to 1 ms and the ratio between states is set to Dimm (73%) and Dfree (27%). The distribution of D* values from the simulated interconverting species with transient binding fits well to a single species at D = 0.35 μm2s-1, matching our observed Dslow population. D) Transition categorization analysis of simulated interconverting species with varying binding dwell times. Tracks were simulated with 15 ms exposure times and in time-lapse with 15 ms exposures followed by a 35 ms delay, then categorized in the same way as experimental data in Fig. 4B, and the reduction in the total fraction of molecules categorized as immobile between normal and time-lapse simulations was plotted against the binding time. A range of binding times between 30 and 70 ms agree with the observed reduction in immobile ParC in time-lapse experiments (reduced from 35% to 14%). E) Short axis (X axis) and long axis (Y axis) elliptical Gaussian fit widths at 750 ms exposure time for Pol1 + MMS, ParC, MukB and ParC in time-lapse experiment where 1 s exposures were followed by 4 s delays. We used thresholds of < 160 nm short axis-width and < 200 nm long axis-width to identify immobile molecules determined from the Pol1 control where immobile and mobile molecules are clearly resolvable (dashed line) (Uphoff et al, 2013; Fig.S1C). F) On-time distributions for bound ParC at 0.5 s exposure, 0.75 s exposure, 1 s exposure times and 1 s = 4 s time-lapse conditions. Exponential fits (solid lines) and photobleaching-corrected binding time distributions (dashed circled lines). Error bars shows standard deviation of three experimental repeats. Fitting ranges give 95% confidence intervals.

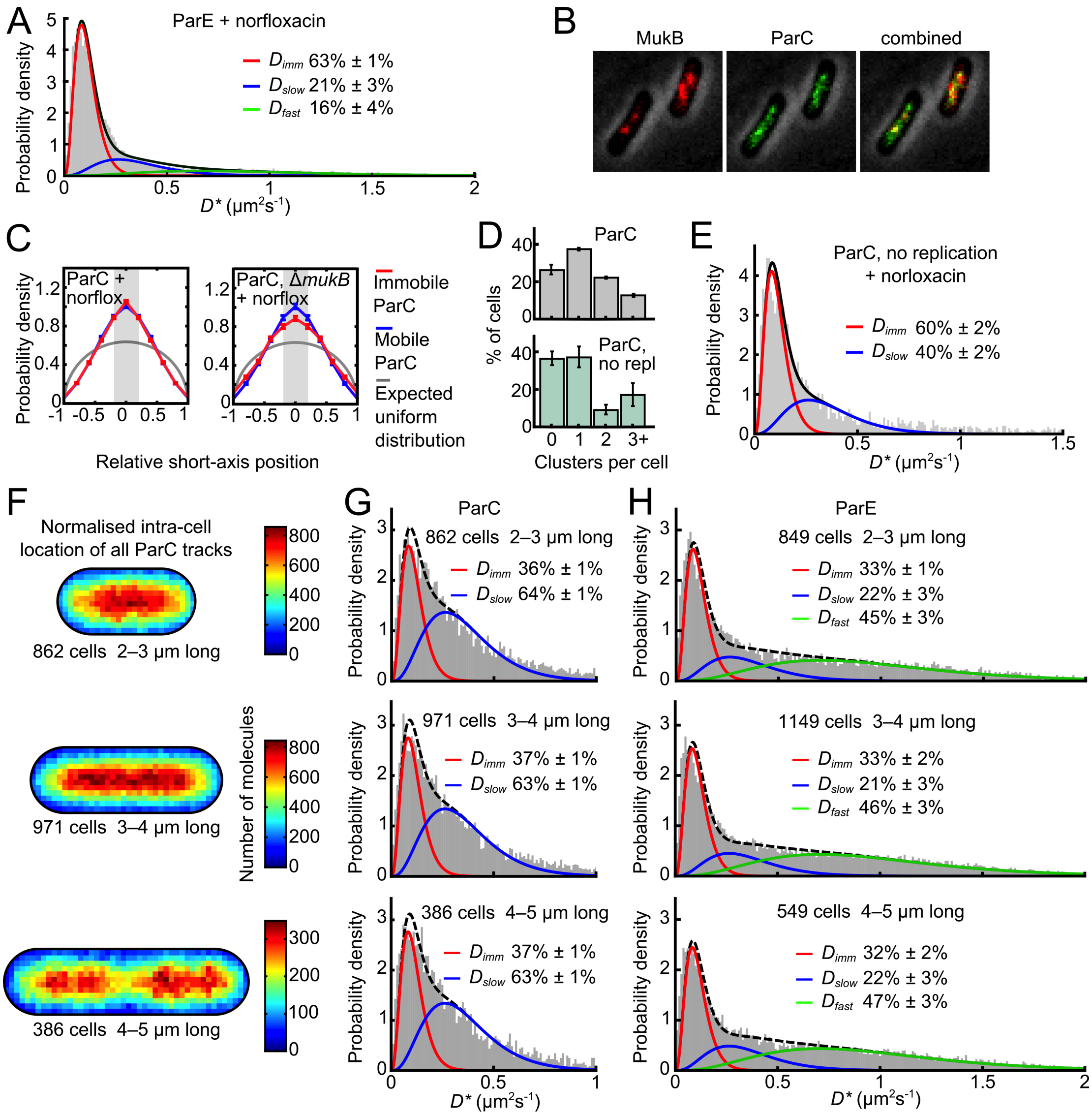

Figure S5. Catalytically Active TopoIV. Related to Figure 4.

A) Distribution of D^* values for 17001 ParE molecules after 10min treatment with norfloxacin. ParE and ParC had a similar fraction of immobile molecules after norfloxacin treatment. B) Example cells treated with norfloxacin. Epifluorescence microscopy shows MukB-mcherry foci are still present and some of ParC-YPet clusters are associated with these MukB foci. C) The probability density of ParC molecules across the cell short-axis in 858 cells treated with norfloxacin (left). Dashed grey line shows the expected distribution for molecules uniformly distributed throughout a cylindrical cell volume. The mobile and immobile populations have similar distributions, both with 36 % of molecules located in the central 20 % of the width (grey shaded area). The distribution of ParC molecules across the cell short-axis in 463 Δ*mukB* cells treated with norfloxacin (right). The mobile population has 37 % of molecules located in the central 20 % of short-axis, whereas the immobile

population shows fewer molecules (33 %) in the central region. D) The number of ParC clusters per cell in cells not undergoing replication (right). We observed a small decrease in number of clusters in cells not undergoing replication, compared to the entire population of cells (right), indicating that ParC foci are not dependent on the presence of replication. Instead, persistence of ParC foci supports our conclusion that their formation is dependent on MukB. Only small cells without replisomes were used in this analysis, therefore a small decrease in the number of ParC foci was expected due to the smaller average size compared to the entire population of cells. E) Distribution of *D** values for 2479 ParC molecules in cells not undergoing replication after 10min treatment with norfloxacin. Distributions of D* were fitted with two species model with both D values constrained. F) Normalized 2D histograms showing the average spatial distribution of ParC localizations from many cells. Binning by cell length from short to long cells shows progression through the cell cycle, with short cells (2-3 μm long) having a single centrally located nucleoid, and longer cells (4-5 μm long) having two clearly separate nucleoids. G) Distribution of ParC *D** values from 862 short cells (top), 971 medium-length cells (middle), and 386 long cells (bottom). Distributions of D* were fitted with two species model with both D values constrained. H) Distribution of ParE *D** values from 849 short cells (top), 1149 medium-length cells (middle), and 549 long cells (bottom). Distributions of D* were fitted with three species model with all D values constrained. Fitting ranges give 95% confidence intervals

Table S1. Bacterial Strains. Related to the Experimental Procedures; section 1. Bacterial Strains and Cell Preparation

Strain	Genotype[a]
AB1157	F^-, λ^-, rac^-, *thi-1, hisG4, Δ(gpt-proA)62, argE3, thr-1, leuB6, kdgK51, rfbD1, araC14, lacY1, galK2, xylA5, mtl-1, tsx-33, supE44(glnV44), rpsL31(strR), qsr'-0, mgl-51* (Bachmann, 1972)
PZ103	*parE:: PAmCherry kan*
KK10	*parC::PAmCherry kan*
PZ147	*parC::PAmCherry kan; mukB::mYPet frt;* $gyrA^{L83}$ *tet*

PZ120	*parE:: PAmCherry kan; mukB:: mYPet frt;* $gyrA^{L83}$ *tet*
PZ124	*parE:: PAmCherry kan; dnaN:: mYPet frt;* $gyrA^{L83}$ *tet*
PZ125	*parC:: PAmCherry kan; dnaN:: mYPet frt;* $gyrA^{L83}$ *tet*
PK13	*parC:: PAmCherry kan; tetR:: mYPet frt; tetO at ori1 gen*
PK16	*parC:: PAmCherry kan; tetR:: mYPet frt; tetO at ter3 gen*
PZ109	*parE:: PAmCherry kan; tetR:: mYPet frt; tetO at ori1 gen*
PZ114	*parE:: PAmCherry kan; tetR:: mYPet frt; tetO at ter3 gen*
PZ128	*parE:: PAmCherry frt; ΔmukB kan; dnaN:: mYPet frt;* $gyrA^{L83}$ *tet*
PZ129	*parC:: PAmCherry frt; ΔmukB kan; dnaN:: mYPet frt;* $gyrA^{L83}$ *tet*
Ab238	*MukB:: PAmCherry kan (Badrinarayanan et al., 2012)*
PZ122	*parC::yPet kan; mukB::mcherry frt;* $GyrA^{L83}$ *tc*
KG52	*lacO240::hyg* at *ori1; tetO240::gen* at *ter3; plac-lacI-mCherry* at *leuB, plac-lacI-tetR-mCerulean* at *galK, mYPet-DnaN kan*
AB1157 MukB-mYPet	MukB::mYPet *kan*

[a] All strains were in the AB1157 background and were constructed in this study unless otherwise stated.
[b] Abbreviations: *kan*, kanamycin resistance gene; *tet*, tetracycline resistance gene; *gen*, gentamicin resistance gene; hyg, hygromycin resistance gene; *frt*, FLP site-specific recombination site.

Table S2. Oligonucleotides and Plasmids. Related to the Experimental Procedures; section 1. Bacterial Strains and Cell Preparation

Name	Description	Reference
ParCpam cherryfor	GTGTTGAGATCGACTCTCCTCGCCGTGCCAGCA GCGGTGATAGCGAAGAG**TC**GGCTGGCTCCGCTG	This study
ParCpam cherryrev	TTTCATCCGGCGTTCCTTGCAAGCGGGAGGAAAC AGCGCCCTCCCCGGCATATATGAATATCCTCCTT AGTTC	This study
ParEpam cherryfor	ATCGCCGCAACTGGTTGCAAGAGAAAGGCGACA TGGCGGAGATTGAGGTT TCG GCT GGC TCC GCT GCT GGT TC	This study
ParEpam	TAATCCTGCCTTGTTTGCCCGGCCATCCTGACCG	This study

cherryrev	GGCAATGTTCTTTCCT GAGGATCCCATATGAATATCCTCC	
pZ68	ParC-CTD (amino acid residues 497-806) cloned into pBAD24 NcoI and XbaI cloning sites	This study
pZ65	Full length ParE cloned into pBAD24 using NcoI and XbaI cloning sites	This study

Table S3. Behavior of Fluorescent Fusions and strain overexpressing ParC-CTD (KG52). Related to the Experimental Procedures; section 1. Bacterial Strains and Cell Preparation

	AB1157	ParC-PAmCherry (KK10)	ParE-PAmCherry (PZ103)	KG52 pBAD24	ParC-CTD Overexpression pZ68
Generation time	86 ± 3 min	85 ± 5 min	81 ± 11 min	95 ± 1.3 min	101 ± 3.9 min
Cell length	3.1 ± 0.5 μm	3.3 ± 0.7 μm	3.1 ± 0.6 μm	3.7 ± 0.8 μm	3.7 ± 0.8 μm
Anucleate cells	not determined	0/86	1/77	0/80	1/68

Table S4. Exponential time constants for PAmCherry photobleaching t_{bleach}, measured ParC on-times t_{on}, and corrected ParC binding times t_{bound}. (±SD). Related to Figure 3 and Figure S4.

	500 ms	750 ms	1000 ms
$t_{bleach\ [S]}$	0.423	0.647	0.672
$t_{on\ [S]}$	0.328 ± 0.02	0.503 ± 0.03	0.484 ± 0.02
$t_{bound\ [S]}$	1.68 ± 0.52	1.94 ± 0.22	1.83 ± 0.16

Movie 1. Visualization of 3D structured illumination images showing the organization of MukB foci inside living cells. Related to Figure 2 and Figure S3. MukB--mYPet fluorescence is shown in green, and DAPI stained DNA is shown in red.
